# Supplementary material for: Validation of CHERG’S Verbal Autopsy-Social Autopsy (VASA) tool for ascertaining determinants and causes of under-five child mortality in Pakistan
Source: PLoS One. 2023 Dec 18;18(12):e0278149. doi: 10.1371/journal.pone.0278149 (PMC10727362; doi:10.1371/journal.pone.0278149)
Supplement: S2 File — (PDF) [file pone.0278149.s002.pdf]

فقط ایک جواب

ایک سے زیادہ جوابات

بچوں کی اموات پر تحقیق کا سوالنامہ

فارم نمبر ☐☐☐☐-☐☐☐☐-☐☐

کچی آبادی نمبر | گھر نمبر | بچہ نمبر

VERBAL/SOCIAL AUTOPSY GENERAL INFORMATION (FOR SBs, NN & CHILD DEATHS 0—59 MONTHS OLD)

Section 1: Background about the died: سیکشن-1: مرحوم بچہ کی معلومات

اس سیکشن کو انٹرویو سے پہلے پر کریں۔

G1.1	گھر کا پتہ	مکان نمبر ____<br>محلہ / گلی / روڈ ____<br>ڈسٹرکٹ ____<br>ٹاؤن ____<br>یونین کونسل ____<br>علاقہ ____	موبائل نمبر ☐☐☐☐☐☐☐☐☐☐☐☐☐☐<br>گھر کا فون نمبر ☐☐☐☐☐☐☐☐☐☐☐☐☐☐<br>(کوئی ایک)
	گھر کے رستے کی نشاندہی کریں / چیدہ چیدہ نشانیاں بیان کریں		
	گھر کے محل وقوع کا نقشہ بنایئے۔		
G1.2	مرحوم بچہ کا مکمل نام	☐☐☐☐☐☐☐☐☐☐☐☐☐☐☐☐☐☐☐☐<br>☐☐☐☐☐☐☐☐☐☐☐☐☐☐☐☐☐☐☐☐	
G1.3	مرحوم بچہ کی جنس<br>فقط ایک جواب	☐1- بچہ<br>☐2- بچی	
G1.4	مرحوم بچہ کی تاریخ پیدائش		__ __/__ __/__ __ __ __<br>D D M M Y Y Y Y<br>(معلوم نہیں = 99/99/9999)
G1.5	مرحوم بچہ کی تاریخ وفات		__ __/__ __/__ __ __ __<br>D D M M Y Y Y Y<br>(معلوم نہیں = 99/99/9999)
G1.6	مرحوم بچہ کی آخری معلوم عمر		— — دن: 01 or more → G1.7<br>(معلوم نہیں = 99)<br>___ ___ مہینے → G1.7<br>(معلوم نہیں = 99)<br>___ ___ سال → G1.7<br>(معلوم نہیں = 99)
G1.6.1	کیا یہ بچہ مرا ہوا پیدا ہوا تھا یا بعد میں وفات پائی؟<br>فقط ایک جواب	☐1- مرا ہوا پیدا ہوا تھا<br>☐2- پیدائش کے ایک مہینے کے اندر وفات پائی<br>☐99- ریکارڈ سے معلوم نہیں	

بچوں کی اموات پر تحقیق کا سوالنامہ

فارم نمبر ☐☐☐☐-☐☐☐-☐☐

کچی آبادی نمبر | گھر نمبر | بچہ نمبر

G1.7	مرحوم بچہ کی والدہ کا نام	☐☐☐☐☐☐☐☐☐☐☐☐☐☐☐☐☐☐☐☐☐☐☐ ☐☐☐☐☐☐☐☐☐☐☐☐☐☐☐☐☐☐☐☐☐☐☐	
G1.8	مرحوم بچہ کے والد کا نام	☐☐☐☐☐☐☐☐☐☐☐☐☐☐☐☐☐☐☐☐☐☐☐ ☐☐☐☐☐☐☐☐☐☐☐☐☐☐☐☐☐☐☐☐☐☐☐	
**Section 2: Background about the interview**		انٹرویو کی بنیادی معلومات: اس سیکشن کو انٹرویو سے پہلے پر کریں۔	
G2.1	یہ انٹرویو کس زبان میں ہوا؟	______________________	
G2.2	انٹرویو کرنے والے کا نام	☐☐☐☐☐☐☐☐☐☐☐☐☐☐☐☐☐☐☐☐☐☐☐☐ ☐☐☐☐☐☐☐☐☐☐☐☐☐☐☐☐☐☐☐☐☐☐☐☐	
G2.3	انٹرویو کرنے کی تمام کوششوں کی تاریخ اور نتائج لکھئے	تاریخ	نتیجہ
G2.3.1	انٹرویو کی پہلی کوشش	__ __/__ __/__ __ __ __ D D M M Y Y Y Y	نتیجہ ☐
G2.3.2	انٹرویو کی دوسری کوشش	__ __/__ __/__ __ __ __ D D M M Y Y Y Y	نتیجہ ☐
G2.3.3	انٹرویو کی تیسری کوشش	__ __/__ __/__ __ __ __ D D M M Y Y Y Y	نتیجہ ☐
G2.4	انٹرویو کرنے کی تاریخ	__ __/__ __/__ __ __ __ D D M M Y Y Y Y	
G2.5	انٹرویو شروع کرنے کا وقت	__ __/__ __ H R M M	
G2.6	انٹرویو کس تاریخ کو ختم ہوا	__ __/__ __/__ __ __ __ D D M M Y Y Y Y	حتمی نتیجہ: ☐
G2.7	انٹرویو کس وقت ختم ہوا	__ __/__ __ H R M M	
	5۔ انٹرویو سے انکار ہو گیا<br>6۔ اس پتہ پر کوئی جوابدھندہ نھیں رہتا<br>7۔ اس پتہ پر کوئی گھر نہیں<br>8۔ انٹرویو جاری ہے		انٹرویو کے نتائج کے کوڈز:<br>1۔ انٹرویو مکمل ھو گیا<br>2۔ انٹرویو مکمل نہیں ہوا<br>3۔ انٹرویو ملتوی ہو گیا<br>4۔ جوابدہندہ نہ مل سکا
G2.8	سپروائیزر کے فارم چیک کرنے کی تاریخ	__ __/__ __/__ __ __ __ D D M M Y Y Y Y	
G2.9	کس تاریخ کو یہ فارم کمپیوٹر میں اندراج کیا گیا۔	__ __/__ __/__ __ __ __ D D M M Y Y Y Y	

بچوں کی اموات پر تحقیق کا سوالنامہ

فارم نمبر

کچی آبادی نمبر	گھر نمبر	بچہ نمبر
☐☐☐☐	☐☐☐☐	☐☐

انٹرویو شروع کیا جاتا ہے

سب سے پہلے، آپ اپنا تعارف کرائیں اور آنے کا مقصد بتائیں۔ مرحوم بچہ کی والدہ یا اس شخص سے جو بچہ کی بیماری کے وقت بچہ کے ساتھ موجود تھا سے بات کرنے کی امید ظاہر کریں۔ اگر اس وقت انٹرویو ممکن نہیں تو کوئی اور وقت طہ کریں۔

"میرا نام ------ ہے۔ میں بچوں کی اموات پر تحقیق کرنے والے پروجیکٹ کی طرف سے ایک انٹرویو کرنے والا ہوں۔ مجھے معلوم ہوا ہے کہ آپ کے گھر میں ایک بچہ کی وفات ہوئی ہے۔ مجھے اس واقعے پر بہت افسوس ہے۔ برائے مہربانی میرا افسوس قبول کریں۔ ہم بچوں کی صحت کی بہتری کے لئے، پچھلے ایک سال میں واقع ہونے والی اموات کے بارے میں معلومات جمع کررہے ہیں۔ لہاظہ میں مرحوم بچہ کی والدہ یا قریبی شخص سے کچھ سوالات پوچھنا چاہتا ہوں۔ ان سوالات کا تعلق موت سے پہلے واقع ہونے والی بیماری اور متعلقہ واقعات کے بارے میں ہوگا۔"

Section 3: Consent: سیکشن 3: اجازت نامہ

اجازت نامہ جوابدہندہ کو پڑھ کر سنائیں۔ اس سے پوچھیں کہ اس کو کوئی سوال پوچھنا ہے۔ تمام سوالات کے جواب دینے کے بعد اس سے اس کی اجازت دریافت کریں کہ کیا وہ اس تحقیق میں شامل ہونا چاہتا ہے؟

G3.1	کیا جوابدہندہ نے تحقیق میں شامل ہونے کی اجازت دی؟<br>فقط ایک جواب	☐ 1۔ جی ہاں<br>☐ 2۔ جی نھیں	☐ 2 → جوابدہندہ کا شکریہ ادا کریں اور انٹرویو ختم کریں

Section 4: Information about the respondent: سیکشن 4: جوابدہندہ کے بارے میں معلومات

اب میں آپ سے کچھ سوالات آپ کے بارے میں پوچھنا چاہتا ہوں۔

G4.1	آپ کا کیا نام ہے؟	☐☐☐☐☐☐☐☐☐☐☐☐☐☐☐☐☐☐☐☐☐☐☐☐☐<br>☐☐☐☐☐☐☐☐☐☐☐☐☐☐☐☐☐☐☐☐☐☐☐☐☐	
G4.2	آپ کی جنس کیا ہے<br>فقط ایک جواب	☐ 1- مرد<br>☐ 2- عورت	☐
G4.3	مرحوم بچہ سے آپ کا کیا تعلق ہے؟<br>فقط ایک جواب	1. Mother---------------- والدہ -1<br>2. Father---------------- والد -2<br>3. Grandmother---------- دادی / نانی -3<br>4. Grandfather----------- دادا / نانا -4<br>5. Aunt------------- خالہ / چچی / پھپھی / تائی -5<br>6. Uncle------------ چچا / ماموں / تایا / پھپھا -6<br>7. Brother---------------- بھائی -7<br>8. Sister------------------ بہن -8<br>9. Birth attendant *(specify type)* ——— دائی -9<br>10. Other male *(specify)* ——— دیگر مرد -10<br>11. Other female *(specify)* ---- دیگر خاتون -11	☐<br>☐<br>☐<br>☐<br>☐<br>☐<br>☐<br>☐<br>☐ ________<br>☐ ________<br>☐ ________
G4.4	آپ کی عمر کتنی ہے؟۔		__ __ سال<br>*(DK = 99)*
G4.5	آپ اسکول یا مدرسے میں کتنے سال کی تعلیم حاصل کرچکے / چکی ہیں؟		__ __ سال<br>*(<1 = 00; DK = 99)*<br>G4.6 سوال پر جائیں، اگر 6 سال سے زیادہ ہے۔
G4.5.1	برائے مہربانی ان جملوں کو پڑھ کر بتلیے۔<br>☐ 1۔ بالکل نہیں پڑھ سکتا<br>☐ 2۔ تھوڑا بہت پڑھ سکتا ہے<br>☐ 3۔ پورا پڑھ سکتا ہے<br>☐ 4۔ کارڈ موجود نہیں<br>فقط ایک جواب	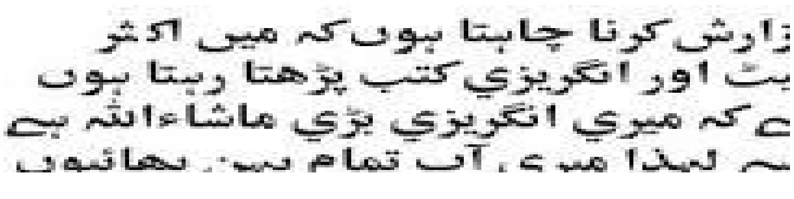<br>زارش کرنا چاہتا ہوں کہ میں اکثر<br>یث اور انگریزی کتب پڑھتا رہتا ہوں<br>ے کہ میری انگریزی بڑی ماشاءاللہ ہے<br>[illegible]	

اب میں آپ سے آپ کے خاندان کے بارے میں کچھ سوالات پوچھوں گا۔ یاد رہے کہ تمام معلومات انتہائی خفیہ رکھی جائیں گی۔

G4.6	اس گھر میں کتنے افراد رہتے ہیں؟	__ __ افراد *(DK = 99)*
G4.7	اس گھر میں کتنے کمرے موجود ہیں؟	__ __ کمرے *(DK = 99)*

بچوں کی اموات پر تحقیق کا سوالنامہ

فارم نمبر: | | | | | - | | | | - | | |

بچہ نمبر — گھر نمبر — کچی آبادی نمبر

G4.8	کیا اس گھر میں کھانا پکانے کے لئے کوئی خاص جگہ موجود ہے؟<br>فقط ایک جواب	☐1۔جی ہاں<br>☐2۔جی نھیں<br>☐99۔معلوم نھیں	
	کیا اس گھر میں مندرجہ زیل اشیاء موجود ہیں؟	☐1۔بجلی<br>☐2۔ریڈیو<br>☐3۔ٹی وی<br>☐4۔فرج<br>☐5۔گھر کا فون<br>☐6۔موبائل فون<br>☐7۔کمپیوٹر<br>☐8۔سائیکل<br>☐9کار یا ٹرک<br>☐10۔گھر میں پانی کے زخیرے کا ٹینک<br>☐11۔پانی کا کنواں<br>☐12۔پانی مہیا کرنے والا دکاندار<br>☐13۔ندی یا تالاب	
G4.10	اس گھر میں کس طرح کا بیت الخلاء موجود ہے؟<br>فقط ایک جواب	☐1۔فلش ٹائلٹ<br>☐2۔گڑھے والا ٹائیلٹ<br>☐3۔جھاڑیوں / میدان میں<br>☐4۔دیگر (وصاحت کریں)<br>☐99۔معلوم نہیں	☐<br>__________
G4.11	اس گھر میں کھانا پکانے کے لئے ایندھن کے طور پر کس چیز کا استعمال کیا جاتا ہے؟<br>فقط ایک جواب	☐1۔کوئلہ<br>☐2۔لکڑی<br>☐3۔مٹی کا تیل<br>☐4۔بجلی<br>☐5۔گیس<br>☐6۔گوبر<br>☐7۔دیگر (وصاحت کریں)<br>☐99۔معلوم نہیں	☐<br>__________
G4.12	اس گھر کا فرش کس چیز کا بنا ہے؟<br>فقط ایک جواب	☐1۔قدرتی مٹی<br>☐2۔سیمنٹ<br>☐3۔لکڑی<br>☐4۔ٹائلز<br>☐5۔دیگر (وصاحت کریں)<br>☐99۔معلوم نہیں	☐<br>__________

فارم نمبر ☐☐☐☐-☐☐☐-☐☐

کچی آبادی نمبر | گھر نمبر | بچہ نمبر

بچوں کی اموات پر تحقیق کا سوالنامہ

Section 5: Information about others at the interview

سیکشن 5: انٹرویو کے وقت دیگر موجود افراد کی معلومات:

G5.7	کیا اس انٹرویو کے وقت کوئی اور افراد موجود ہیں؟<br>فقط ایک جواب	☐1- جی ہاں<br>☐2- جی نہیں ⟵ **Go to G5.9**	
G5.8	جواب دہندہ کے علاوہ اور کتنے لوگ انٹرویو کے وقت موجود ہیں؟		دیگر افراد ___ ___<br>*(DK = 99)*
G5.9	جو لوگ انٹرویو کے وقت موجود ہیں، نیچے دیے گئے ان کے نام کے آگے ڈبے میں "ٹک" کا نشان لگائیے۔		

	انٹرویو کے دوران موجود شخص کا مرحوم بچہ کے ساتھ رشتہ	انٹرویو کے دوران موجود شخص پر "ٹک" لگائیں۔	مردہ پیدائش یا پہلے مہینے کی اموات میں		پہلے مہینے یا بڑے بچوں کی اموات میں	
			کیا یہ شخص حمل کے 9 مہینے کے دوران ماں کے ساتھ موجود تھا/تھی؟	کیا یہ شخص بچہ کی پیدائش کے وقت ماں کے ساتھ موجود تھا/تھی؟	کیا یہ شخص بچہ کی بیماری کے وقت بچہ کے ساتھ موجود تھا/تھی؟	کیا یہ شخص بچہ کی وفات کے وقت بچہ کے ساتھ موجود تھا/تھی؟
.1	Mother والدہ	☐			☐1- جی ہاں<br>☐2- جی نہیں	☐1- جی ہاں<br>☐2- جی نہیں
.2	Father والد	☐	☐1- جی ہاں<br>☐2- جی نہیں	☐1- جی ہاں<br>☐2- جی نہیں	☐1- جی ہاں<br>☐2- جی نہیں	☐1- جی ہاں<br>☐2- جی نہیں
.3	Grandmother دادی/نانی	☐	☐1- جی ہاں<br>☐2- جی نہیں	☐1- جی ہاں<br>☐2- جی نہیں	☐1- جی ہاں<br>☐2- جی نہیں	☐1- جی ہاں<br>☐2- جی نہیں
.4	Grandfather دادا/نانا	☐	☐1- جی ہاں<br>☐2- جی نہیں	☐1- جی ہاں<br>☐2- جی نہیں	☐1- جی ہاں<br>☐2- جی نہیں	☐1- جی ہاں<br>☐2- جی نہیں
.5	Aunt خالہ/ پھپھی/ چاچی	☐	☐1- جی ہاں<br>☐2- جی نہیں	☐1- جی ہاں<br>☐2- جی نہیں	☐1- جی ہاں<br>☐2- جی نہیں	☐1- جی ہاں<br>☐2- جی نہیں
.6	Uncle ماموں/چاچا/ تایا	☐	☐1- جی ہاں<br>☐2- جی نہیں	☐1- جی ہاں<br>☐2- جی نہیں	☐1- جی ہاں<br>☐2- جی نہیں	☐1- جی ہاں<br>☐2- جی نہیں
.7	Brother بھائی	☐	☐1- جی ہاں<br>☐2- جی نہیں	☐1- جی ہاں<br>☐2- جی نہیں	☐1- جی ہاں<br>☐2- جی نہیں	☐1- جی ہاں<br>☐2- جی نہیں
.8	Sister بہن	☐	☐1- جی ہاں<br>☐2- جی نہیں	☐1- جی ہاں<br>☐2- جی نہیں	☐1- جی ہاں<br>☐2- جی نہیں	☐1- جی ہاں<br>☐2- جی نہیں
.9	Traditional birth attendant دائی	☐	☐1- جی ہاں<br>☐2- جی نہیں	☐1- جی ہاں<br>☐2- جی نہیں	☐1- جی ہاں<br>☐2- جی نہیں	☐1- جی ہاں<br>☐2- جی نہیں
.10	Other male (specify: دیگر مرد (وصاحت کریں) ____________)	☐	☐1- جی ہاں<br>☐2- جی نہیں	☐1- جی ہاں<br>☐2- جی نہیں	☐1- جی ہاں<br>☐2- جی نہیں	☐1- جی ہاں<br>☐2- جی نہیں
.11	Other female (specify دیگر خاتون (وصاحت کریں) ____________)	☐	☐1- جی ہاں<br>☐2- جی نہیں	☐1- جی ہاں<br>☐2- جی نہیں	☐1- جی ہاں<br>☐2- جی نہیں	☐1- جی ہاں<br>☐2- جی نہیں

بچوں کی اموات پر تحقیق کا سوالنامہ

فارم نمبر | کچی آبادی نمبر | گھر نمبر | بچہ نمبر

VA Section 1: Background (FOR STILLBIRTHS, NEONATAL & CHILD DEATHS 0—59 MONTHSOLD)<br>مردہ پیدا ہونے والا بچہ، بارہ مہینے سے پہلے مرنے والا بچہ اور 0-59 مہینے سے پہلے مرنے والے بچوں کے بارے میں بنیادی معلومات کا سیکشن				
V1.1	کیا یہ مرحوم بچہ اکیلا پیدا ہوا تھا یا جڑواں پیدا ہوا تھا؟<br>فقط ایک جواب<br>*[If two or more children are born at the same time, it is counted as a multiple birth, even if one or more of the babies are born dead.]*	☐1. اکیلا پعدا ہوا تھا → Go to V1.3.<br>☐2. جڑواں پیدا ہوا تھا → Go to V1.2<br>☐99. معلوم نہیں → Go to V1.3.		
V1.2	مرحوم بچے کا ترتیب کے حساب سے کونسا نمبر تھا؟<br>فقط ایک جواب	1. پہلا 2. دوسرا<br>3. تیسرا یا زیادہ 99. معلوم نہیں	☐☐	
V1.3	کیا والدہ ابھی زندہ ہے؟<br>فقط ایک جواب	☐1. جی ہاں → Go to V1.6.<br>☐2. جی نہیں → Go to V1.4.		
V1.4	کیا والدہ کی وفات بچے کی پیدائش کے دوران ہوئی یا بعد میں؟<br>فقط ایک جواب	☐1. دوران → Go to V1.6.<br>☐2. بعد میں → Go to V1.5.<br>☐99. معلوم نہیں → Go to V1.6.		
V1.5	بچے کو پیدا کرنے کے کتنے عرصے بعد والدہ کی وفات ہوئی؟<br>*[if less than 24 hours, record "00" days, If less than 28 days, record days, if 28 days or more record months]*		دن ___ ___<br>*(DK = 99)*<br>مہینے ___ ___<br>*(DK = 99)*	
V1.6	بچہ کس جگہ پیدا ہوا؟<br>فقط ایک جواب	☐1. اسپتال<br>☐2. صحت کا کوىء اور مرکز<br>☐3. مرکز جاتے ہوئے، رستے میں<br>☐4. گھر پر<br>☐ 5. دیگر(وضاحت کریں)<br>☐99. معلوم نہیں	☐<br>-<br>______	
V1.7	پیدائش کے وقت مرحوم بچے کا جسم کیسا تھا؟<br>فقط ایک جواب	☐ 1- بہت چھوٹا Very small<br>☐ 2- چھوٹا Smaller than usual<br>☐ 3- درمیانا About average<br>☐ 4- بڑا Larger than usual<br>☐99. معلوم نہیں Don't know		
V1.8	پیدائش کے وقت بچے کا کتنا وزن تھا؟		گرام ___ ___ ___ ___<br>*(DK = 9999)*	
V1.9	مرحوم بچے کی کیا جنس تھی؟<br>فقط ایک جواب	☐1. بچہ<br>☐2. بچی<br>☐99. معلوم نہیں		
V1.10	بچے کی تاریخ پیدائش کیا ہے؟<br>*Compare the delivery date just stated by the respondent to the birth date from the prior record (G1.4). Discuss any inconsistency with the respondent to confirm or correct the stated delivery date. You cannot change the prior record's date.*		___ ___/___ ___/___ ___ ___ ___<br>D D M M Y Y Y Y<br>*(DK = 99/99/9999)*	
V1.11	کیا بچہ زندہ پیدا ہوا تھا یا مردہ؟<br>فقط ایک جواب	. زندہ → select -B in V1.15<br>. مردہ → select -A in V1.15<br>99. معلوم نہیں	☐☐	
V1.12	کیا بچہ کبھی رویا/روئی؟<br>فقط ایک جواب	☐1. جی ہاں → select-B in V1.15<br>☐2. جی نہیں → select -A in V1.15<br>☐99. معلوم نہیں		

V1.13	کیا بچے نے کبھی حرکت کری<br>فقط ایک جواب	☐1. جی ھاں ← select -B in V1.15<br>☐2. جی نہیں ← select -A in V1.15<br>☐99. معلوم نہیں	
V1.14	کیا بچہ نے کبھی سانس لی؟<br>فقط ایک جواب	☐1. جی ھاں ← select -B in V1.15<br>☐2. جی نہیں ← select -A in V1.15<br>☐99. معلوم نہیں	
V1.15	فقط ایک جواب	A. ☐Stillbirth ~~مرا ہوا~~ پیدا ہوا → V1.16.<br>B. ☐Live birth زندہ پیدا ہوا → V1.20.	
	**Stillbirths -** مرا ھوا پیدا بچہ		
V1.16	کیا پیدائش کے وقت مرحوم بچہ کے جسم پر چوٹ یا نیل کا کوئی نشان موجود تھا؟<br>فقط ایک جواب	☐1. جی ھاں<br>☐2. جی نہیں<br>☐99. معلوم نہیں	
V1.17	کیا پیدائش کے وقت مرحوم بچہ کے جسم کی جلد بہت نرم یا گلی ہوئی تھی؟<br>فقط ایک جواب	☐1. جی ھاں<br>☐2. جی نہیں<br>☐99. معلوم نہیں	
V1.18	کیا پیدائش کے وقت بچہ کے جسمانی اعصاء میں کوئی نقص تھا؟<br>فقط ایک جواب	☐1. جی ھاں ← Go to V1.19.<br>☐2. جی نہیں ← Go to S.3.1.<br>☐99. معلوم نہیں ← Go to S.3.1.	
V1.19	اگر "ہاں"، تو وہ کونسے نقص تھے؟<br>ایک سے زیادہ جوابات	☐1۔ سر بہت چھوٹا تھا؟<br>☐2۔ سر بہت بڑا تھا؟<br>☐3۔ پیٹھ یا ریڑھ کی ہڑی میں کوئی نقص تھا<br>☐4۔ کوئی اور نقص (وصاحت کریں)	ہاں / نہیں<br>----------------
	ہدایات: یہاں پر رک جائیں ---۔ اگر ماں کی معلومات حاصل کرنی ہے تو سوال نمبر 3.1-S پر جائیں۔		
	**Live births -** زندہ پیدا ہوا بچہ		
V1.20	جب بچہ بیمار ہوا تو اس وقت بچے کی عمر کتنی تھی؟		___ ___ دن (DK = 99)<br>___ ___ مہینے (DK = 99)<br>___ ___ سال (DK = 99)
V1.21	یہ بیماری کتنے عرصے چلی؟		___ ___ دن (DK = 99)<br>___ ___ مہینے (DK = 99)
V1.22	بچے کی وفات کس جگہ ہوئی؟<br>فقط ایک جواب	☐1۔ اسپتال<br>☐2۔ صحت کا دوسرا مرکز<br>☐3۔ مرکز جاتے ہوئے، رستے میں<br>☐4۔ گھر میں<br>☐5۔ دیگر (وصاحت کریں).<br>☐99۔۔ معلوم نہیں	____________________
V1.24	بچے کی تاریخ وفات کیا ہے؟<br>*Compare the date of death just stated by the respondent to the date of deathfrom the prior record (G1.5). Discuss any inconsistency with the respondent toconfirm or correct the stated date. You cannot change the prior record'sdate.*		__ __/__ __/__ __ __ __<br>D D M M Y Y Y Y<br>(DK = 99/99/9999)

فارم نمبر [ ][ ][ ][ ]-[ ][ ][ ]-[ ][ ]

کچی آبادی نمبر — گھر نمبر — بچہ نمبر

بچوں کی اموات پر تحقیق کا سوالنامہ

FOR OFFICIAL PURPOSES ONLY

V1.25	**AGE AT DEATH-بچے کی عمر، وفات کے وقت**<br>**Record only the calculated age <u>OR</u> the stated age. First try to calculate the age. If this is not possible, then ask the respondent for the child's age at death.**<br>بچے کی عمر حساب لگا کر یا والدین سے پوچھ کر ہی نیچے درج کریں۔ پہلے کوشش کر کے حساب لگا --- اگر یہ ممکن نہیں تو جوابدہندہ سے بچے کی وفات کے وقت اس کی عمر پوچھیں۔	
	**بچے کی عمر کا حساب**<br>*Record the delivery date from V1.10:*___ ___/___ ___/___ ___ ___ ___<br>D D M M Y Y Y Y<br>*(Don't Know = 99/99/9999)*<br>*Record the date of death from V1.24:*___ ___/___ ___/___ ___ ___ ___<br>D D M M Y Y Y Y<br>*(Don't Know = 99/99/9999)*<br>*Now, if possible, calculate the age at death (V1.24 – V1.10). If only the month and year are known, you may still be able to calculate the approximate age in months or years. Discuss the calculated age with the respondent:* I have calculated that the child was (about) <CALCULATED AGE> at death. Is this correct?<br>*If the respondent does not agree with the calculated age, then again discuss the delivery date and date of death to make sure that these are correct. If the calculated age at death cannot be resolved, then go below to the "STATED AGE" box.*<br>*Once the age at death is calculated, check V1.20 and V1.21 to make sure that the age at illness onset and the illness duration are consistent with the age at death. For example, the age at onset + duration cannot be greater than the age at death.*<br>*[Record days if less than 28 days—if less than 24 hours, record "00" days; Record months if 28 days-11 months; Record years if 1 year or older.]*	___ ___ Days ***(if < 28 days)***<br>*(DK = 99)*<br>___ ___ Months ***(if 1-11 months)***<br>*(DK = 99)*<br>___ ___ Years ***(if 1 year or older)***<br>*(DK = 99)*
	(صرف اس وقت پوچھیں، جب آپ حساب نہیں لگا پائیں)۔ **<u>جوابدہندہ کی طرف سے بتائی گئی عمر۔</u>**<br>**بچے کی وفات کے وقت، بچے کی عمر کتنی تھی؟**	___ ___ Days ***(if < 28 days)***<br>*(DK = 99)*<br>___ ___ Months ***(if 1-11 months)***<br>*(DK = 99)*<br>___ ___ Years ***(if 1 year or older)***<br>*(DK = 99)*
V1.26	**کیا مرحوم بچہ کی وفات؟**<br>☐ **1**۔ پہلے مہینے میں ہوئ؟<br>☐ **2**۔ ایک مہینے اور 5 سال کے دوران ہوئ → **Go to S5b.1**<br>**فقط ایک جواب**	

فارم نمبر | ☐☐☐☐-☐☐☐-☐☐

کچی آبادی نمبر | گھر نمبر | بچہ نمبر

بچوں کی اموات پر تحقیق کا سوالنامہ

SA Module 3 and VA Section 2: Maternal history (FOR STILLBIRTHS AND NN DEATHS < 28 DAYSOLD)

والدہ کی حمل اور زچگی کی کہانی:

اب میں آپ (والدہ) سے اس مرحوم بچے کی پیدائش سے پہلے کے حمل اور زچگی کے دوران کے واقعات اور آپ کی صحت کے بارے میں کچھ سوالات کرنا چاہتا ہوں۔

S3.1	کیا حمل سے پہلے ماں کو ان بیماریوں میں سے سے کوئی بیماری تھی؟ کیا ان بیماریوں کا علاج کرایا؟<br>ایک سے زیادہ جوابات	1۔ بلڈ پریشر کی تکلیف<br>2۔ دل کی تکالیف<br>3۔ ذیابیطس<br>4۔ جھٹکا یا مرگھی کا دورہ<br>5۔ دیگر (وضاحت کریں).	جی ہاں (نشان لگائیں): 1. ☐ 2 / 1. ☐ / 1. ☐ / 1. ☐ / 1. ☐<br>علاج کرایا؟: 1. ☐ 2. ☐ 9. ☐ / 1. ☐ 2. ☐ 9. ☐ / 1. ☐ 2. ☐ 9. ☐ / 1. ☐ 2. ☐ 9. ☐ / 1. ☐ 2. ☐ 9. ☐<br>________________
S3.2	کیا حمل کے دوران والدہ نے قبل از پیدائش حمل کی دیکھ بھال کے لیے کسی سے مدد مانگی حاصل کی؟	☐1۔ جی ہاں → Go to S.3.2.1.<br>☐2۔ جی نہیں → Go to S.3.3.<br>☐99۔ معلوم نہیں → Go to S.3.3.	
S3.2.1	آپ نے کس سے مدد حاصل کی؟<br>ایک سے زیادہ جوابات	☐1۔ ڈاکٹر<br>☐2۔ دائی<br>☐3۔ پڑوسی/ رشتہ دار/دوست. → Go to S.3.3.<br>☐ 4۔ دیگر (وضاحت کریں) → Go to S.3.3.<br>(________________)<br>☐99۔ معلوم نہیں → Go to S.3.3.	→ سوال SQ3.3 پر منتقل
S3.2.2	اس حمل کے دوران آپ نے کتنی مرتبہ اپنا چیک اپ کرایا؟		دفعا ___ ___<br>*(DK = 99)*
S3.2.3	اس حمل کے دوران آپ نے آخری مرتبہ اپنا چیک اپ حمل کے کونسے مہینے میں کرایا؟		مہینے ___ ___<br>*(DK = 99)*
S3.2.4	اس حمل کے دوران معالج نے کس طرح آپ کی مدد کی؟<br>ایک سے زیادہ جوابات	1۔ کیا معالج نے ماں کا بلڈ پریشر چیک کیا؟<br>2۔ کیا معالج نے ماں کا پیشاب کا ٹیسٹ کیا؟<br>3۔۔ کیا معالج نے ماں کا خون کا ٹیسٹ کیا؟<br>4۔ کیا معالج نے ماں کو طاقت ور غذائی اجناس استعمال کرنے کی ہدایت کری؟<br>5۔ کیا معالج نے حمل کے دوران ظاہر ہونے والی خطرناک علامات کے بارے میں ماں کو آگاہ کیا؟<br>6۔ کیا معالج نے ماں کو آگاہ کیا کہ ان خطرناک علامات کے ظاہر ہونے کی	☐<br>☐<br>☐<br>☐<br>☐<br>☐
S3.3	آپکے خیال میں، حمل کے دوران، ان میں سے کن علامات ظاہر ہونے کی صورت میں معالج سے فوری مدد کی ضرورت ہونی چاہیئے؟<br>ایک سے زیادہ جوابات	1۔ [illegible] صورت میں کہاں رابطہ کرنا چاہی<br>2. جھٹکا یا مرگھی کا دورہ<br>3. سر میں شدید درد اور نظر کی دہندھلا ہونا.<br>4. بخار اور کمزوری ہونا<br>5. پیٹ میں شدید درد ہونا<br>6. سانس میں دشواری ہونا.<br>7. ہر 20 منٹ میں حمل کے پیٹ کا درد ہونا.<br>8. پانی کی تھیلی کا پھٹنا<br>9. خون / چپچپا مواد کا آنا<br>10. ان میں سے کوئ علامت نہیں ہے	☐<br>☐<br>☐<br>☐<br>☐<br>☐<br>☐<br>☐<br>☐<br>☐
S3.4	کیا اس حمل سے پہلے ماں نے بچے میں جھٹکے سے بچاؤ کے لیے اپنے بازو پر کوئی حفاظتی ٹیکہ لگوایا۔<br>فقط ایک جواب	☐1۔ جی ہاں → Go to S.3.4.1.<br>☐2۔ جی نہیں → Go to S.3.5.<br>☐99۔ معلوم نہیں → Go to S.3.5.	

بچوں کی اموات پر تحقیق کا سوالنامہ

فارم نمبر [ ][ ][ ][ ]-[ ][ ][ ]-[ ][ ]

کچی آبادی نمبر | گھر نمبر | بچہ نمبر

S3.4.1	اس حمل کے دوران، ماں نے جھٹکے سے بچاؤ کا ٹیکا کتنی دفعا لگوایا؟		___ دفعا<br>(DK = 9)
S3.5	کیا ماں نے اس حمل سے پہلے کسی بھی وقت، جھٹکا (ٹیٹنس) سے خود کے یا بچے کے بچاؤ کے لئے کوئی ٹیکا لگایا؟<br>**فقط ایک جواب**	☐1. جی ہاں ←<br>☐2. جی نہیں ← **Go to S.3.5.1.**<br>☐99. معلوم نہیں ← **Go to S.3.6.**	
S3.5.1	اس حمل سے پہلے ماں نے کتنی دفعہ جھٹکے سے بچاؤ کا ٹیکا لگایا؟<br>[اگر 7، یا 7 سے زیادہ، تو صرف 7 لکھیں]		___ دفعا<br>(DK = 9)
S3.6	کیا اس حمل کے دوران ماں مچھر دانی کے نیچے سوئی؟<br>(فقط ایک جواب)	☐1. جی ہاں (اکثر)<br>☐ 2. جی ہاں (کبھی کبھار)<br>☐3. جی نہیں<br>☐99. معلوم نہیں	
S3.7	اس حمل کے دوران کیا ماں نے ملیریا سے بچاؤ کی ادویات استعمال کریں؟<br>(فقط ایک جواب)	☐1. جی ہاں ← **Go to S.3.7.1.**<br>☐2. جی نہیں ← **Go to V.2.1.**<br>☐99. معلوم نہیں	
S3.7.1	اس حمل کے دوران ماں نے کتنی دفعہ ان ادویات کا استعمال کیا؟		___ ___ دفعا<br>(DK = 99)
V2.1	اب میں حمل کے دوران آنے والی مشکلات کے بارے میں آپ سے کچھ سوالات کرنا چاھتا ھوں۔<br>کیا حمل کے آخری 3 مہینے میں ماں کو ان حمل کی پیچیدگیوں میں سے کسی بھی پیچیدگی کا سامنا کرنا پڑا؟<br>**(ایک سے زیادہ جوابات)**	☐1۔ جھٹکا/ مرگی کا دورہ<br>☐2۔ بلڈ پریشر کا بڑھنا<br>☐3۔ خون کی کمی<br>☐4۔ شوگر کی بیماری<br>☐5۔ سر میں شدید درد<br>☐6۔ نظر کا دھندلا ہونا<br>☐7۔ بستر سے اٹھنے میں کمزوری محسوس کرنا<br>☐8۔ پیٹ میں شدید درد<br>☐9۔ تیز سانس یا سانس لینے میں دشواری<br>☐10۔ چہرے کی سوجن<br>☐11۔ پیداءش سے پہلے رحم سے خون آنا<br>☐12۔ پیداءش سے پہلے رحم سے بہت خون آنا<br>☐13۔ بخار<br>☐14۔ رحم سے بدبودار پانی کا اخراج<br>☐15۔ بچہ کا سر پہلے باہر آیا۔<br>☐16۔ بچے کی نال پہلے باہر آئی<br>☐17۔ بچے کی نال بچے کی گردن پر لپٹ گی<br>☐18۔ دیگر پیچیدگیاں (وضاحت کریں)<br>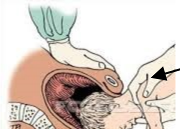<br>............	___
V2.2*	کیا بچے کی پیدائش کے فوراً بعد ماں کی صحت کو ان میں سے کوئی پیچیدگیاں ہوءیں؟<br>**(ایک سے زیادہ جوابات)**	☐1۔ جھٹکا/ مرگی کا دورہ<br>☐2۔ رحم سے بہت زیادہ خون آنا<br>☐3۔ بخار<br>☐4۔ رحم سے بدبودار رطوبت کا اخراج<br>☐5۔ پیٹ میں درد	**Definitions**<br>Vagina رحم<br>Discharge مادہ/ پانی<br>Pregnancy حمل<br>Labor زچگی
V2.2	یہ حمل کتنے مہینے چلا؟		___ ___ مہینے<br>DK = 99)<br>**(Any value other than 99 → V2.**

بچوں کی اموات پر تحقیق کا سوالنامہ

فارم نمبر |_|_|_|_|-|_|_|_|-|_|_|

کچی آبادی نمبر   گھر نمبر   بچہ نمبر

V2.3	کیا بچے کی پیدائش اپنے وقت پر ہوئی، وقت سے پہلے ہوئی یا وقت کے بعد ہوئی؟<br>فقط ایک جواب	□1- وقت سے پہلے ہوئی<br>□2- وقت پر ہوئی<br>□3- وقت کے بعد ہوئی<br>□99- معلوم نہیں	
V2.4	کیا بچہ پیدا ہونے سے کچھ دن پہلے حرکت کر رہا تھا؟<br>فقط ایک جواب	□1- جی ہاں<br>□2- جی نہیں<br>□99- معلوم نہیں	
V2.5	ماں کو بچے کی حرکت کا اندازہ کب ہوا؟		پیدائش سے ــــ ــــ گھنٹے قبل<br>*(DK = 99)*<br>پیدائش سے ــــ ــــ دن قبل<br>*(DK = 99)*
V2.6	کیا پانی کی تھیلی، لیبر سے پہلے یا لیبر کے دوران پھٹی؟<br>فقط ایک جواب	1- پہلے → **Go to V2.7.**<br>2- دوران → **Go to V2.8.**<br>99- معلوم نہیں → **Go to V2.8.**	<u>لیبر (زچگی) کی تعریف:</u><br>لیبر شروع ہوتا ہے جب پیٹ میں درد یا کھنچاؤ ہر 20 منٹ کے اندر ہو۔
V2.7	پانی کی تھیلی، لیبر سے کتنے عرصے پہلے پھٹی؟		ــــ ــــ گھنٹے<br>*(DK = 99)*
V2.8	پانی کا کیا رنگ تھا؟<br>فقط ایک جواب	□ 1- ہرا یا کتھئی (براؤن)<br>□ 2- کوئی رنگ نہیں (نارمل)<br>□3- دیگر (وضاحت کریں) ______________<br>□99- معلوم نہیں	
V2.9	کیا پانی بدبودار تھا؟<br>فقط ایک جواب	□1- جی ہاں<br>□2- جی نہیں<br>□99- معلوم نہیں	
V2.10	لیبر اور ڈیلیوری (زچگی) کتنے عرصے جاری رہی؟<br>*[Record "00" if less than 1 hour.]*		ــــ ــــ گھنٹے<br>*(DK = 99)*<br>(1 گھنٹے سے کم کی صورت میں 00 لکھیں)
S3.8	بچے کی پیدائش کہاں واقع ہوئی؟<br>فقط ایک جواب	1. اسپتال□<br>**2. اسپتال کے علاوہ کوئی دوسرا صحت کا مرکز□**<br>**3. صحت کے مرکز جاتے ہوئے، (رستے میں)□**<br>4. **گھر میں**□<br>*5.* دیگر (وضاحت کریں)______________ □<br>99. معلوم نہیں□	1–3: **Go to S.3.9, THEN S3.10(B)**<br>4–99: **Go to S.3.9, THEN S3.10(A)**
S3.9	یہ کس کا فیصلہ تھا کہ اس بچہ کی پیدائش اس جگہ پر ہو؟<br>فقط ایک جواب	1- بچہ کی ماں کا 2- بچہ کے والد کا<br>3- بچہ کی نانی کا 4- بچہ کی دادی کا<br>5- بچہ کے دادا کا 6- دیگر (وضاحت کریں)<br>99- معلوم نہیں	□□
[redacted]	(A) کیا ماں کو کوئی ایسی مشکلات تھیں جن کی وجہ سے وہ بچہ کی پیدائش کروانے صحت کے مرکز نہ جا سکی؟<br>(B) کیا ماں کو بچہ کی پیدائش کروانے صحت کے مرکز جانے کے لیے کسی مشکلات پر قابو پانے کی ضرورت پڑی؟<br>فقط ایک جواب	□1- جی ہاں<br>□2- جی نہیں<br>□99- معلوم نہیں	1: **S.3.10.1**<br>2, 99: **S.3.11**

فارم نمبر									
کچی آبادی نمبر				گھر نمبر			بچہ نمبر		

بچوں کی اموات پر تحقیق کا سوالنامہ

S3.10.1	صحت کے مرکز جانے کے لیئے ماں کو کونسی مشکلات کا سامنا کرنا پڑا؟ ایک سے زیادہ جوابات)	9. ماں سمجھتی تھی کہ اس بیماری کا روایتی علاج ہے۔ □<br>10. ماں اتنی بیمار تھی کہ وہ سفر کے قابل نہیں تھی۔ □<br>11. ماں یہ سمجھتی تھی کہ چاہے وہ علاج کرائے یا نہیں، بچے نے لازمی انتقال ہی کرنا ہے۔ □<br>12. کافی رات ہو چکی تھی۔ □<br>13. مرکز میں موجود مرد حضرات سے کا سامنا کرنے سے کتراتی تھی۔ □<br>14. دیگر (وضاحت کریں) --------- ---------□<br>99. معلوم نہیں۔□	1. ماں نہیں سمجھ سکی کہ وہ اتنی بیمار ہے کہ اسے صحت کے مرکز جانا چاہیئے۔□<br>2. اس کو مرکز لے جانے لے لیئے گھر پر کوئ نہیں موجود تھا۔□<br>3. ماں کے پاس اتنا وقت نہیں تھا کہ وہ مرکز جا سکے۔□<br>4. کسی اور نے اس بات کا تعین کرنا تھا کہ آیا ماں کو مرکز جانا چاہیئے یا نہیں۔ □<br>5. گھر سے مرکز کا فاصلہ بہت زیادہ تھا۔□<br>6. کوئ سواری موجود نہیں تھی۔ □<br>7. ماں کے پاس پیسے نہیں تھے□<br>8. صحت کے مرکز میں موجود سہولیات سے مطمئن نہیں تھی۔ □
S3.11	صحت کے مرکز میں کس نے بچے کی ڈیلیوری کری؟ فقط ایک جواب	□ 6. دیگر (وضاحت کریں) ________<br>□99- معلوم نہیں	□1- ڈاکٹر<br>□2- نرس<br>□3- رشتہ دار، پڑوسی یا دوست<br>□4- خود<br>□5- دائ
S3.2	زچگی کے کتنے وقت بعد دائ/ڈاکٹر ماں کے پاس آئ؟		دن ___ ___ (DK = 99)<br>گھنٹے ___ ___ (DK = 99)
S3.13	کیا دائ/ڈاکٹر نے تصویروں کے زریعے ماں کو زچگی کی صورتحال سے آگاہ کیا؟	□1- جی ہاں<br>□2- جی نہیں<br>□99- معلوم نہیں	□
S3.14	کیا ڈیلیوری کرنے والے یا والی فرد نے ڈیلیوری سے پہلے صابن سے ہاتھ دھوئے یا دستانے پہنے؟ فقط ایک جواب	□ 1- ہاں، صابن سے ہاتھ دھوئے<br>□2- ہاں، دستانے پہنے<br>□3- نہیں 99- معلوم نہیں	
S3.15	بچہ کی پیدائش کے وقت، ماں کو کس سطح پر لٹایا گیا؟ فقط ایک جواب	□5- مٹی کے کچے فرش پر<br>□6- دیگر (وضاحت کریں)________<br>□99- معلوم نہیں	□1- زچگی کا بستر<br>□2- دھلا ہوا پکا فرش<br>□3- بغیر دھلا ہوا، پکا فرش<br>□ 4- پکا فرش مکنٹوش/کور
V2.17	یہ ڈیلیوری کس طرح کی تھی؟ فقط ایک جواب	1- بغیر اپریشن کے، لیکن قینچی کے زریعے بچے کو باہر نکالا گیا۔ □<br>2- بغیر اپریشن کے، قینچی کے بغیر□<br>3- بغیر اپریشن کے،(معلوم نہیں)<br>4- اپریشن کے زریعے□<br>99- معلوم نہیں□	
V2.18	زچگی کے دوران (مگر ڈیلیوری سے پہلے) کیا ماں نے کسی قسم کا انجکشن لگوایا تھا؟ فقط ایک جواب	□1- جی ہاں<br>□2- جی نہیں<br>□99- معلوم نہیں	

فارم نمبر	☐☐☐☐-☐☐☐-☐☐	بچوں کی اموات پر تحقیق کا سوالنامہ
	کچی آبادی نمبر گھر نمبر بچہ نمبر	

<table>
<tr><td colspan="5"><u>Module 4:</u> ماں کی (حمل کے دوران) پیچیدگیوں کے لیئے حاصل کی گئ مدد کی معلومات
Read: Now, I would like to ask you some questions about (your / the mother’s) care-seeking during the pregnancy with <NAME>.</td></tr>
<tr><td>S4.1</td><td>کیاماں کو حمل کے آخری 3 مہینون میں یا ڈیلیوری کے وقت آگے دی ئے گ ئے علامات کا سامنا کرنا پڑا؟

اگر "ہاں"، تو ان کی تفصیلات، آخری 2 کالم میں بیان کریں۔

(ایک سے زیادہ جوابات

1 2 3</td><td>1. مرگھی یا جھٹکا
2. بلڈ پریشر کا بڑھ جانا
3. خون کی شدید کمی
4. شوگر کا مرض
5. سر میں شدید درد
6. نظر کا دہندہلا ہونا
7. شدید کمزوری
8. پیٹ میں شدید درد
9. سانس میں تکلیف
10. چہرے کا سوجھنا
11. زچگی سے پہلے رحم سے خون آنا
12. زچگی یا ڈیلیوری کے دوران رحم سے شدید خون انا
13. بخار
14. رحم سے بدبودار رطوبت کا خارج ہونا
15. 9 مہینے سے پہلے بچے کی پیدائش
16. زچگی سے 6 گھنٹے پہلے پانی کا خارج ہونا
17. 12 گھنٹے یا زیادہ کی زچگی
18. دیگر (وضاحت کریں)
19. حمل کے آخری 3 مہینے میں کوئ علامات نہیں تھیں
20. زچگی سے پہلے کوئ علامات نہیں تہیں</td><td>حمل کے آخری 3 مہینے مین
☐
☐
☐
☐
☐
☐
☐
☐
☐
☐
☐
☐
☐
☐
☐
☐
☐
☐
....
→</td><td>زچگی سے پہلے / دوران / DK
1. ☐ 2. ☐ 9. ☐
1. ☐ 2. ☐ 9. ☐
1. ☐ 2. ☐ 9. ☐
1. ☐ 2. ☐ 9. ☐
1. ☐ 2. ☐ 9. ☐
1. ☐ 2. ☐ 9. ☐
1. ☐ 2. ☐ 9. ☐
1. ☐ 2. ☐ 9. ☐
1. ☐ 2. ☐ 9. ☐
1. ☐ 2. ☐ 9. ☐
1. ☐ 2. ☐ 9. ☐
1. ☐ 2. ☐ 9. ☐
1. ☐ 2. ☐ 9. ☐
1. ☐ 2. ☐ 9. ☐
1. ☐ 2. ☐ 9. ☐
1. ☐ 2. ☐ 9. ☐
1. ☐ 2. ☐ 9. ☐
1. ☐ 2. ☐ 9. ☐
☐ → S4.11</td></tr>
<tr><td>S4.2</td><td>کیاماں نے زچگی سے پہلے ظاہر ہونے والی علامات کے لی ئے کسی فرد یا مرکز سے مدد حاصل کی؟

فقط ایک جواب</td><td>☐1. جی ہاں → Go to S.4.2.1
☐2. جی نہیں → Go to S.4.4 (A)
☐99. معلوم نہیں → Go to S.4.4 (A)</td><td colspan="2">☐</td></tr>
<tr><td>S4.2.1</td><td>ماں نے کہاں سے مدد حاصل کی؟</td><td>1. اسپتال
2. گورنمنٹ کلینک
3. پرائیویٹ کلینک
4. علاقے کی نرس یا دائ
5. عطائ ڈاکٹر
6. رشتہ دار، پڑوسی یا دوست
7. دیگر (وضاحت کریں)

99. معلوم نہیں</td><td colspan="2">1. ☐
2. ☐ ┐
3. ☐ ├ → First complete S.4.3.., then Go to S.4.4 (B)
4. ☐ ┘
5. ☐ ┐
6. ☐ ├ → Go to S.4.4 (A)
7. ☐ ┘

99. ☐</td></tr>
<tr><td>S4.3</td><td>وہ کونسی علامات تہیں، جن کے لی ئے، ماں نے زچگی سے پہلے کسی فرد یا صحت کے مرکز سے مدد حاصل کی؟
(ایک سے زیادہ جوابات)</td><td>1. جھٹکا یا مرگھی☐
2. بلڈ پریشر کا بڑھنا ☐
3. خون کی کمی☐
4. شوگر کا مرض ☐
5. سر میں شدید درد ☐
6. نظر کا دہندہلا ہونا☐
7. بہت زیادہ کمزوری محسوس کرنا☐
8. پیٹ میں شدید درد ☐
9. سانس میں تکلیف☐</td><td colspan="2">10. چہرے کا سوجھنا ☐
11. زچگی سے پہلے رحم سے خون آنا ☐
12. بخار.☐
13. رحم سے بدبودار رطوبت کا خارج ہونا ☐
14. زچگی سے 6 گھنٹے پہلے پانی کا خارج ہونا☐
15. دیگر (سوال S4.1 میں دی گئ علامات)
(وضاحت کریں)_______________</td></tr>
</table>

S4.4	A. کیاماں کو کوئ ایسی مشکلات تھیں جن کی وجہ سے وہ اوپر دی گئی علامات کا معائنہ کرانے صحت کے مرکز نہ جا سکی؟<br>B. کیاماں کو اوپر دی گئی علامات کا معائنہ کرانے صحت کے مرکز جانے کے لی ئے کسی مشکلات پر قابو پانے کی ضرورت پڑی؟<br>(فقط ایک جواب)	☐1. جی ہاں<br>☐2. جی نہیں<br>☐99. معلوم نہیں	
S4.4.1	صحت کے مرکز جانے کے لی ئے ماں کو کونسی مشکلات کا سامنا کرنا پڑا؟<br>(ایک سے زیادہ جوابات)	9. ماں سمجھتی تھی کہ اس بیماری کا حل صرف روایتی علاج ہے۔ ☐<br>10. ماں اتنی بیمار تھی کہ وہ سفر کے قابل نہیں تھی۔ ☐<br>11. ماں یہ سمجھتی تھی کہ چاہے وہ علاج کرائے یا نہیں، بچے نے لازمی انتقال ہی کرنا ہے۔ ☐<br>12 . مرکز میں موجود مرد حضرات کا سامنا کرنے سے کتراتی تھی۔ ☐<br>☐13. دیگر (وضاحت کریں) --------- ---------<br>☐99. معلوم نہیں۔	1. ماں نہیں سمجھ سکی کہ وہ اتنی بیمار ہے کہ اسے صحت کے مرکز جانا چاہیئے۔☐<br>2. اس کو مرکز لے جانے لے لیئے گھر پر کوئ نہیں موجود تھا۔☐<br>3. ماں کے پاس اتنا وقت نہیں تھا کہ وہ مرکز جا سکے۔☐<br>4. کسی اور نے اس بات کا تعین کرنا تھا کہ آیا ماں کو مرکز جانا چاہیئے یا نہیں۔ ☐<br>5. گھر سے مرکز کا فاصلہ بہت زیادہ تھا۔☐<br>6. کوئ سواری موجود نہیں تھی۔ ☐<br>7. ماں کے پاس پیسے نہیں تھے☐<br>8. صحت کے مرکز میں موجود سہولیات سے مطمئن نہیں تھی۔ ☐
S4.5	کیا صحت مہیا کرنے والے فرد یا مرکز نے ماں کو دوسرے کسی فرد یا مرکز میں بھیجا؟<br>فقط ایک جواب	☐1. جی ہاں → Go to S.4.5.1<br>☐2. جی نہیں → Go to S.4.6<br>☐99. معلوم نہیں → Go to S.4.6	
S4.5.1	کیا بھیجے گۓ دوسرے مرکز میں ماں گئی؟<br>فقط ایک جواب	☐1. جی ہاں<br>☐2. جی نہیں<br>☐99. معلوم نہیں	
S4.6	زچگی سے پہلے ظاہر ہونے والی علامات کے معاینے کے لی ئے ماں کل کتنے (صحت مہیا کرنے والے) افراد یا مراکز کے پاس معاینے کے لیے گئی؟		صحت مہیا کرنے والے افراد یا —— ——<br>مراکز *(DK = 99)*
S4.7	کیاماں ان علامات کے علاج کے لیے اسپتال میں داخل ہوئ؟<br>فقط ایک جواب	☐1. جی ہاں<br>☐2. جی نہیں<br>☐99. معلوم نہیں	
S4.8	بتای ئے کہ ان مرکز یا مراکز نے ماں کو اوپر بتائی گئی علامات کے لی ئے گھر پر علاج کی لی ئے کیا ہدایات دیں؟<br>(ایک سے زیادہ جوابات)	☐6. کچھ عرصے بعد دوبارہ چیک اپ کرانے کو کہا<br>☐7. اگر صحت زیادہ خراب ہو تو دوبارہ آنے کو کہا<br>☐ 8. دیگر (وضاحت کریں)<br>☐9. کوئ ہدایت نہیں دی<br>99. معلوم نہیں☐	☐1. منہ سے اینٹی بایوٹک کھانے کو کہا<br>☐2. منۃ سے سے ملیریا کی دوا کھانے کو کہا<br>☐3. منۃ سے بلڈ پریشر کی دوا کھانے کو کہا<br>☐4. منۃ سے کوئ اور دوا کھانے کو کہا<br>☐5. گھر پر آرام کرنے کو کہا
S4.9	کیاماں ان ہدایات پر عمل کرنے کے قابل تھی؟<br>فقط ایک جواب	1- جی ہاں ☐ → S4.10(B)<br>2- جی نہیں☐ → S4.10(A)<br>99. معلوم نہیں☐	

بچوں کی اموات پر تحقیق کا سوالنامہ

فارم نمبر ☐☐☐☐-☐☐☐☐-☐☐

کچی آبادی نمبر گھر نمبر بچہ نمبر

S4.10	A. کیا ماں کو کوئی ایسی مشکلات تھیں جن کی وجہ سے وہ ان ہدایات پر عمل نہ کر سکی؟<br>B. کیا ماں کو ان ہدایات پر عمل کرنے کے لیٔے کسی مشکلات پر قابو پانے کی ضرورت پڑی؟<br>**فقط ایک جواب**	1- جی ہاں☐ → S4.10.1<br>2- جی نہیں☐ → S4.11<br>99- معلوم نہیں☐	
S4.10.1	وہ مشکلات کیا تھیں؟<br>**فقط ایک جواب**	☐6- دی گئ ہدایات کی کوئ ضرورت نہیں تھی<br>7- پیدا ہونے والے بچے کو ان ہدایات سے ☐ ہو سکتا ہے<br>8- ماں یہ سمجھتی تھی کہ چاہیے وہ ان ہدایات پر عمل کرے یا نہیں، بچے کی موت لازمی ہے<br>9- دیگر ________ 99- معلوم نہیں	☐1- ہدایات سمجھ میں نہ آسکیں<br>2- ماں کے پاس ان ہدایات پر عمل کرنے کا وقت نہیں تھا☐<br>☐3- کسی دوسرے شخص سے اجازت کی ضرورت تھی<br>4- ان ہدایات پر عمل کرنے کیلیئے کافی رقم کی ضرورت تھی- ☐<br>5- اس مسئلہ کا حل صرف روایتی علاج میں تھا☐
_2: Refer to S4.1: If no labor or delivery symptoms → Inst_8 اب ہم بات کرتے ہیں زچگی (لیبر) اور ڈیلیوری کی علامات پر			
S4.11	سوال نمبر .S.4.1 میں دی گئی علامات کو دوبارہ پڑھیں اور اگلے سوال پر جائیں۔		
S4.12	جب یہ علامات ظاہر ہوئیں، اس وقت ماں کہاں تھی؟<br>**فقط ایک جواب**	☐1- گھر پر<br>☐2- صحت کے مرکز جاتے ہوئے، راستے پر<br>☐3- صحت کے مرکز میں<br>☐ 4- دیگر (وضاحت کریں)<br>☐99- معلوم نہیں	________
S4.13	کیا ماں نے زچگی (لیبر) یا ڈیلیوری کی علامات کے علاج کے لیٔے کسی سے مدد حاصل کی؟ یا کیا ماں کو کوئی مدد حاصل ہوئی؟<br>**فقط ایک جواب**	1- جی ہاں☐ → S4.13.1<br>2- جی نہیں☐ → S4.17<br>99- معلوم نہیں☐ → S4.17	
S4.13.1	ان علامات سے نمٹنے کے لئے، ماں نے ان میں سے کونسا عمل سب سے پہلے اختیار کیا؟<br>**فقط ایک جواب**	1. گھر پر علاج → S4 15(A)<br>☐2- اسپتال سے مدد حاصل کی<br>☐3- گورنمنٹ کلینک سے مدد حاصل کی<br>☐4- پرایوٹ کلینک سے مدد حاصل کی<br>☐5- علاقے کی نرس یا مڈوائف سے مدد حاصل کی<br>(2–5 → S4.15(B))<br>☐6- دوا بیچنے والے سے مدد حاصل کی<br>☐7- روحانی معالج سے مدد حاصل کی<br>☐ 8- دیگر (وضاحت کریں)<br>(6–8 → S4.15(A))<br>☐99- معلوم نہیں → S4.16	→ S4.15(B)<br>→ S4.15(A)<br>________
S4.14	کس نے اس بات کا فیصلہ کیا کہ یہ عمل، اس وقت کے حساب سے مناسب تھا؟<br>**فقط ایک جواب**<br>[redacted]	☐1- بچہ کی ماں نے<br>☐2- بچہ کے والد نے<br>☐3- بچہ کی دادی نے<br>☐4- بچہ کے دادا نے<br>☐5- بچہ کی نانی نے<br>☐ 6- دیگر (وضاحت کریں)<br>☐99- معلوم نہیں	________

S4.15	(A) کیا ماں کو کوئ پریشانیاں تھیں جن کی وجہ سے وہ اس وقت صحت کے مرکز نہیں جا سکی؟ (B)<br>(C) کیا ماں کوصحت کے مرکز جانے کے لیئے، کسی مشکلات پر قابو پانے کی ضرورت پڑی؟<br>**فقط ایک جواب**	□**1. جی ھاں**<br>□**2. جی نہیں**<br>□**99. معلوم نہیں**	
S4.15. 1	وہ مشکلات کیا تھیں؟<br>**ایک سے زیادہ جوابات**	**9. ماں سمجھتی تھی کہ اس بیماری کا حل صرف روایتی علاج ہے۔** □<br>**10. ماں اتنی بیمار تھی کہ وہ سفر کے قابل نہیں تھی۔** □<br>**11. ماں یہ سمجھتی تھی کہ چاہے کہ وہ علاج کرائے یا نہیں، بچے نے لازمی انتقال ہی کرنا ہے۔** □<br>**12. کافی رات ہو چکی تھی۔** □<br>**□13. مرکز میں موجود مرد حضرات کا سامنا کرنے سے کتراتی تھی۔**<br>□**14. دیگر (وضاحت کریں)** --------- ---------<br>□**99. معلوم نہیں۔**	**1. ماں نہیں سمجھ سکی کہ وہ اتنی بیمار ہے کہ اسے صحت کے مرکز جانا چاہیئے۔**□<br>**2. اس کو مرکز لے جانے لے لیئے گھر پر کوئ نہیں موجود تھا۔**□<br>**3. ماں کے پاس اتنا وقت نہیں تھا کہ وہ مرکز جا سکے۔**□<br>**4. کسی اور نے اس بات کا تعین کرنا تھا کہ آیا ماں کو مرکز جانا چاہیئے یا نہیں۔** □<br>**5. گھر سے مرکز کا فاصلہ بہت زیادہ تھا۔**□<br>**6. کوئ سواری موجود نہیں تھی۔** □<br>**7. ماں کے پاس پیسے نہیں تھے**□<br>**8. صحت کے مرکز میں موجود سہولیات سے مطمئن نہیں تھی۔** □
S4.16	**کیا ماں نے زچگی (لیبر) یا ڈیلیوری کی علامات کے علاج کے لیئے کسی سے مدد حاصل کی؟**<br>**فقط ایک جواب**	1- جی ہاں□<br>2- جی نہیں□<br>99- معلوم نہیں □	**S4.16.1**<br>***S4.17***
S4.16. 1	ماں نے زچگی (لیبر) یاڈیلیوری کی علامات کے علاج کے لئے کہاں سے مدد حاصل کی؟ □<br>**ایک سے زیادہ جوابات**	□**1- اسپتال**<br>□**2- گورنمنٹ کلینک**<br>□**3- پرائیویٹ کلینک**<br>□**4- علاقے کی نرس یا دائ**<br>□ **5. دیگر (وضاحت کریں)**<br>□**99. معلوم نہیں**	
S.4.8..1 7	سوال SQ3.8 میں دیکھیں کہ بچہ کی کہاں پیدائش ہوئ؟	1- اسپتال میں<br>2- کوئ اور مرکز میں<br>3- مرکز جاتے ہوئے<br>4 - گھر پر<br>5- دیگر ----------------------<br>99- معلوم نہیں	□ 1-3 = Health provider<br>______________
S4.18	کل ملا کر کتنے صحت مہیا کرنے والے افراد یا مراکز سے ماں نے مدد مانگی یا حاصل کی؟		صحت مہیا کرنے والے افراد یا مراکز __ __

بچوں کی اموات پر تحقیق کا سوالنامہ

فارم نمبر ☐☐☐☐-☐☐☐-☐☐

کچی آبادی نمبر　گھر نمبر　بچہ نمبر

S4.19	کیا کوئی خاص علامت تھی، جس کی وجہ سے ماں پہلے صحت کے مرکز کے پاس گئی؟	1- جی ہاں☐<br>2- جی نہیں☐ → S4.21<br>99- معلوم نہیں☐ → S4.21	☐
S4.20	وہ کونسی علامات تھیں؟	1- ☐جھٹکا یا مرگھی<br>2- ☐ بلڈ پریشر کا بڑہنا<br>3☐خون کی کمی<br>4- ☐ سر میں شدید درد<br>5- ☐نظر کا دہندہلا ہونا<br>6- ☐بہت زیادہ کمزوری محسوس کرنا<br>7- ☐ پیٹ میں شدید درد<br>8- ☐سانس میں تکلیف<br>9☐ چہرے کا سوجھنا.	10- ☐ زچگی سے پہلے رحم سے خون آنا..<br>11- زچگی اور ڈیلیوری کے دوران شدید خون انا<br>12☐.بخار<br>13- ☐ رحم سے بدبودار رطوبت کا خارج ہونا<br>14- 9 مہینے سے پہل ھمل کا ختم ہونا<br>15- زچگی سے 6 گھنٹے پہلے پانی کا خارج . ☐ہونا<br>16- زچگی کا 12 گھنٹے سے زیادہ چلنا<br>17- دیگر.(سوال S4.1میں دی گئ علامات) ________(وضاحت کریں)
S4.21	زچگی اور ڈیلیوری کی علامات ظاہر ہونے کے کتنے عرصے بعد اس بات کا فیصلہ کیا گیا کہ اس صحت کے مرکز پر جایا جائے؟		دن __ __<br>(DK = 99)<br>گھنٹے __ __<br>(DK = 99)<br>منٹ __ __<br>(DK = 99)

زچگی اور ڈیلیوری کے سوالات		پہلا صحت کا مرکز	آخری صحت کا مرکز
ماں نے زچگی (لیبر) یا ڈیلیوری کی علامات کے علاج یا ڈیلیوری کے لئے کس سے یا کہاں سے مدد حاصل کی؟	1- گورنمنٹ اسپتال<br>2- پرایوٹ اسپتال(این جی او)<br>3- گورنمنٹ کلینک<br>4- پرائیویٹ کلینک<br>5- گورنمنٹ ڈاکٹر<br>6- پرایوٹ ڈاکٹر (این جی او)<br>7- علاقے کی نرس یا دائ<br>8- دیگر (وضاحت کریں)<br>9- پرائویٹ ڈاکٹر (عطا ئ)<br>10- پرائویٹ ڈاکٹر (؟عطا ئ؟)<br>99- معلوم نہیں	**S4.22**<br>☐☐<br>________<br>مرکز کا نام	**S4.32**<br>☐☐<br>________<br>مرکز کا نام
مدد حاصل کرنے کا ارادہ کرنے (یا کسی دوسرے مرکز یا ڈاکٹر کی طرف ریفرل ملنے) کے کتنے عرصے بعد ماں اس مرکز پر گئی؟		**S4.23**<br>دن __ __<br>(DK = 99)<br>گھنٹے __ __<br>(DK = 99)<br>منٹ __ __<br>(DK = 99)	**S4.33**<br>دن __ __<br>(DK = 99)<br>گھنٹے __ __<br>(DK = 99)<br>منٹ __ __<br>(DK = 99)
کیا اس مرکز پر پہنچنے میں یا مرکز کے اندر (سہولت حاصل کرنے کیلیئے) ماں کو کوئی خرچہ کرنا پڑا؟	1- جی ہاں<br>2- جی نہیں→ S4.25<br>99- معلوم نہیں→ S4.25	**S4.24**<br>☐	**S4.34**<br>☐

بچوں کی اموات پر تحقیق کا سوالنامہ

فارم نمبر ☐☐☐☐-☐☐☐-☐☐

کچی آبادی نمبر　　گھر نمبر　　بچہ نمبر

ماں نے اس خرچہ کیلی ئے کہاں سے پیسوں کا بندوبست کیا؟<br>ایک سے زیادہ جوابات	1. ماں کے پاس تھے<br>2. کسی سے مانگے<br>3. اپنی چیزیں بیچیں<br>4. رشتہ داروں نے مدد کی<br>5. علاقے والوں نے مدد کی<br>6. حکومتی اسکیم<br>7. دیگر<br>99. معلوم نہیں	**S4.24.1**<br>1. ☐<br>2. ☐<br>3. ☐<br>4. ☐<br>5. ☐<br>6. ☐<br>7. ☐<br>99. ☐	**S4.34.1**<br>1. ☐<br>2. ☐<br>3. ☐<br>4. ☐<br>5. ☐<br>6. ☐<br>7. ☐<br>99. ☐
سواری کا کونسا زریعہ اختیار کیا؟<br>ایک سے زیادہ جوابات	1. پیدل گئ<br>2. رکشہ<br>3. بس<br>4. ٹیکسی<br>5. ایمبولنس<br>6. دیگر<br>7. سواری کا بندوبست نہیں ہو سکا<br>99. معلوم نہیں	**S4.25**<br>1. ☐ → *S4.26.1*<br>2. ☐<br>3. ☐<br>4. ☐<br>5. ☐<br>6. ☐<br>7. ☐ → *S4.26.1*<br>99. ☐	**S4.35**<br>1. ☐ → *S4.36.1*<br>2. ☐<br>3. ☐<br>4. ☐<br>5. ☐<br>6. ☐<br>7. ☐ → *S4.36.1*<br>99. ☐
سفر میں کتنا خرچہ آیا؟		**S4.26**<br>روپے __ __ __ __<br>*(DK = 9999)*	**S4.36**<br>روپے __ __ __ __<br>*(DK = 9999)*
کیا ماں اس مرکز پر بچے کی پیدائش سے پہلے پہنچ گی؟	1- ہاں بچے کی پیدائش سے پہلے پہنچ گی<br>2- نہیں، رستے میں بچہ پیدا ہوا	**S4.26.1**<br>☐	**S4.36.1**<br>☐
اس مرکز پر پہنچنے میں ماں کو کتنا وقت لگ؟		**S4.27**<br>گھنٹے __ __<br>*(DK = 99)*<br>منٹ __ __<br>*(DK = 99)*	**S4.37**<br>گھنٹے __ __<br>*(DK = 99)*<br>منٹ __ __<br>*(DK = 99)*
اس مرکز نے آپ کی اور بچہ کی کس طرح مدد کری؟<br>ایک سے زیادہ جوابات	1. بچہ کو آکسیجن دی<br>2. اپکو منہ سے اینٹیبایوٹک دی<br>3. اپکو منہ سے اینٹی ملیریل دوا دی<br>4. اپکو منہ سے بلد پریشر کی دوا دی<br>5. کوئ اور دوا منہ سے دی<br>6. خون روکنے کیلئے دوا دی<br>7. مرگھی روکنے کیلئے دوا دی<br>8. زچگی کو اور نمایاں کرنے کیلئے دوا دی<br>9. زچگی کو ختم کرنے کیلئے دوا دی<br>10. بچے کے پھیپھڑوں کیلئے دوا دی<br>11. ماں کے کولہے یا بازو میں ٹیکا لگایا<br>12. ماں کو ڈرپ لگائ<br>13. ماں کو خون چڑہایا<br>14. ماں کو دکان سے دوا خریدنے کو کہا<br>15. بچہ دانی کی باہر سے مالش کی<br>16. ڈیلیوری کیلیئے اپریشن کیا<br>17. کوئ اور آپریشن کیا *(specify)*<br>18. اسپتال میں داخل کیا<br>19. دیگر *(specify)*<br>20. کچھ نہیں کیا<br>99. Don't know	**S4.28**<br>1. ☐<br>2. ☐<br>3. ☐<br>4. ☐<br>5. ☐ ____<br>6. ☐<br>7. ☐<br>8. ☐<br>9. ☐<br>10. ☐<br>11. ☐<br>12. ☐<br>13. ☐<br>14. ☐<br>15. ☐<br>16. ☐<br>17. ☐ ____<br>18. ☐ stayed ☐☐days<br>19. ☐ ____<br>20. ☐ → *S4.30*<br>99. ☐ → *S4.30*	**S4.38**<br>1. ☐<br>2. ☐<br>3. ☐<br>4. ☐<br>5. ☐ ____<br>6. ☐<br>7. ☐<br>8. ☐<br>9. ☐<br>10. ☐<br>11. ☐<br>12. ☐<br>13. ☐<br>14. ☐<br>15. ☐<br>16. ☐<br>17. ☐ ____<br>18. ☐ stayed ☐☐days<br>19. ☐ ____<br>20. ☐ → *S4.40*<br>99. ☐ → *S4.40*
ماں نے ان سب سہولیات کے حصول کیلی ئے کل کتنا خرچہ کیا؟		**S4.29**<br>روپے __ __ __ __ __<br>*(DK = 99999)*	**S4.39**<br>روپے __ __ __ __ __<br>*(DK = 99999)*

بچوں کی اموات پر تحقیق کا سوالنامہ

فارم نمبر | | | | | - | | | | - | | |

کچی آبادی نمبر — گھر نمبر — بچہ نمبر

کیا پہلے مرکز نے ماں کو دوسرے مرکز کی طرف ریفر کیا؟	1- جی ہاں<br>2- جی نہیں<br>99- معلوم نہیں	**S4.30**<br>☐ *2 or 9 →S4.30.2*	**S4.40**<br>☐ *2 or 9 → S4.40.2*
آپکو دوسرے مرکز کیوں ریفر کیا گیا؟<br>ایک سے زیادہ جوابات	1. مرکز میں علاج کی مہارت نہیں رکھتا تھا............. . . . .<br>2. مرکز میں علاج کی سہولیات موجود نہیں تھیں.........<br>3. مرکز میں مشینیں نہیں تھیں.............<br>4. مرکز میں چیزیں نہیں تھیں.............<br>99. معلوم نہیں..................................	**S4.30.1**<br>1. ☐<br>2. ☐<br>3. ☐<br>4. ☐<br>99. ☐	**S4.40.1**<br>1. ☐<br>2. ☐<br>3. ☐<br>4. ☐<br>99. ☐
کیا بچہ اس مرکز میں پیدا ہوا؟		**S4.30.2**<br>☐	**S4.40.2**<br>☐
اگر ماں اس مرکز میں نہیں جاسکی، تو کیا اس کی کوئی وجوہات تھیں جن کی وجہ سے وہ نہیں جاسکی؟	1- جی ہاں<br>2- جی نہیں<br>99- معلوم نہیں	**S4.31**<br>☐	**S4.41**<br>☐
وہ کونسی وجوہات تھیں؟<br>ایک سے زیادہ جوابات	1. خیال تھا کہ اس کو اب علاج کی ضرورت نہیں. . . . . .<br>**2.** ساتھ جانے کیلیے کوئ نہیں تھا.... . .. .<br>**3.** جانے کا وقت نہیں ملا. . . . .<br>**4.** کسی اور نے منع کردیا.. .. . .<br>**5.** مرکز کا فی دور تھا....... . . . .<br>**6.** کوئ سواری نہیں تھی ....<br>**7.** پیسے نہیں تھا. . . . .<br>**8.** علاج سے مطمئن نہیں تھی.... . . . .<br>**9.** روایتی علاج کی ضرورت تھی. . . . .<br>**10.** بیماری میں سفر کے قابل نہیں تھی ... .<br>**11.** . . خیال تھا کہ چایے علاج کرائے یا نہین، بچہ نے نہیں بچنا....... . . . .<br>**12** کافی رات ہو چکی تھی .. . .<br>**13.** جانے سے پہلے ہی بچہ پیدا ہو چکا تھا......... . . . .<br>**14.** دیگر *(specify)* ....<br>**99.** معلوم نہیں	**S4.31.1**<br>1. ☐<br>2. ☐<br>3. ☐<br>4. ☐<br>5. ☐<br>6. ☐<br>7. ☐<br>8. ☐<br>9. ☐<br>10. ☐<br>11. ☐<br>12. ☐<br>13. ☐<br>14. ☐ ________<br>99. ☐	**S4.41.1**<br>1. ☐<br>2. ☐<br>3. ☐<br>4. ☐<br>5. ☐<br>6. ☐<br>7. ☐<br>8. ☐<br>9. ☐<br>10. ☐<br>11. ☐<br>12. ☐<br>13. ☐<br>14. ☐ ________<br>99. ☐

بچوں کی اموات پر تحقیق کا سوالنامہ

فارم نمبر □□□□-□□□-□□

کچی آبادی نمبر — گھر نمبر — بچہ نمبر

**Module 5a: نومولود بچہ کی ابتدائی نگہداشت**			
S5a.1	بچے کی ناف کاٹنے کیلئے کس چیز کا استعمال کیا گیا؟	1. نیا بلیڈ □<br>2. پرانا بلیڈ □<br>3. قینچی □<br>4. دیگر *(specify)* □.......................................<br>99. **معلوم نہیں** □	________________
S5a.2	ناف کو باندھنے کیلئے کس چیز کا استعمال کیا گیا؟	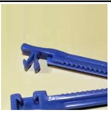<br>1. صاف ستھرے دھاگہ□<br>2. گندا دھاگہ□<br>3. Cord clamp(کورڈ کلیمپ) □<br>4. دیگر *(specify)* □.......................................<br>99. **معلوم نہیں**□	________________
S5a.3	کیا بچہ پیدا ہونے کے بعد، اسکی ناف پر کوئی چیز لگائی گئی تھی؟	1. ہاں□<br>2. نہیں □→ ***V3.1***<br>99. **معلوم نہیں**□ → ***V3.1***	
S5a.3.1	وہ کیا چیز تھی؟	1. الکوحول یا اینٹی سیپٹک □<br>**2. اینٹی بایوٹک کریم یا پوڈر** □<br>3. سرسوں کا تیل یا گھی □<br>4. مٹی یا گوبر □<br>5. دیگر *(specify)* □...................................<br>99. **معلوم نہیں**□	________________
V3.1	پیدائش کے وقت، بچے کے جسم پر کوئی نیل یا چوٹ کے نشانات تھے؟	□**1. جی ہاں**<br>□**2. جی نہیں**<br>□**99. معلوم نہیں**	
V3.2	کیا پیدائش کے وقت بچے کے جسمانی اعصاء میں کوئی نقص تھا؟<br>**(فقط ایک جواب)**	□**1. جی ہاں**<br>□→ ***V3.4*** **2. جی نہیں**<br>□→ ***V3.4*** **99. معلوم نہیں**	
V3.3	اگر "ہاں"، تو وہ کونسے نقص تھے؟<br>**(ایک سے زیادہ جوابات)**	□1۔ سر بہت چھوٹا تھا؟<br>□2۔ سر بہت بڑا تھا؟<br>□3۔ پیٹھ یا ریڑھ کی ہڑی میں کوئی نقص تھا<br>□4۔ کوئی اور نقص (وضاحت کریں)	________________
V3.4	پیدائش کے بعد، کیا بچے نے فوراً سانس لینا شروع کر دی تھی؟	□**1. جی ہاں**<br>□→ ***V3.6*** **2. جی نہیں**<br>□**99. معلوم نہیں**	
V3.5	کیا بچہ کو سانس لینے میں دشواری ہوئی؟	□**1. جی ہاں**<br>□**2. جی نہیں**<br>□**99. معلوم نہیں**	
V3.6	سانس کی دشواری کیلیئے آپ نے کچھ کیا؟	□**1. جی ہاں**<br>□**2. جی نہیں**<br>□**99. معلوم نہیں**	
V3.7	کیا بچہ پیدا ہونے کے فوراً رویا؟	□→ ***V3.9*** **1. جی ہاں**<br>□**2. جی نہیں**<br>□**99. معلوم نہیں**	
V3.8	بچہ پیدا ہونے کے کتنے عرصے بعد رویا؟	1. 5 منٹ کے اندر□<br>**2. 6-30 منٹ کے اندر**□<br>3. 30 منٹ کے بعد□<br>4. کبھی نہیں □ → ***S5a.4***<br>99. **معلوم نہیں** □	

بچوں کی اموات پر تحقیق کا سوالنامہ

فارم نمبر ☐☐☐☐-☐☐☐☐-☐☐

کچی آبادی نمبر — گھر نمبر — بچہ نمبر

V3.9	کیا بچہ روتے روتے چپ ہو گیا؟	1. جی ہاں ☐<br>2. جی نہیں ☐ → S5a.4<br>99. معلوم نہیں ☐ → S5a.4	
V3.10	وفات سے کتنے عرصے پہلے بچے نے رونا روک دیا؟	1. ایک دن سے کم عرصہ ☐<br>2. ایک دن یا اس سے زیادہ ☐<br>99. معلوم نہیں ☐	
S5a.4	پیدائش کے کتنے عرصے بعد بچہ کو غسل دیا گیا؟	1. ایک گھنٹے کے اندر<br>2. ایک گھنٹے اور 23 گھنٹوں کے دوران<br>3. 24 اور 72 گھنٹوں کے دوران (1-3 days)<br>4. 72 گھنٹوں سے زیادہ (3 days)<br>5. غسل نہیں دیا<br>99. معلوم نہیں	
S5a.5	پیدائش کے بعد کیا بچے کو گرم رکھنے کی کوئی کوشش کی گئی؟	1. جی ہاں ☐<br>2. جی نہیں ☐ → S5a.6<br>99. معلوم نہیں ☐ → S5a.6	
S5a.5.1	وہ کوشش کیا تھی؟<br>پیدائش کے کتنے عرصے بعد کیا گیا؟	1. پونچھا اور سکھایا.................................<br>2. کمبل اڑھایا...................... ....<br>3. ماں نے اپنے سینے سے لگا کر رکھا<br>4. انکیوبیٹر ....<br>5. دیگر	Done — پیدائش کے کتنے عرصے بعد: <1hr, <6, 6-24, >24, DK<br>1. ☐...... 1.☐ 2.☐ 3.☐ 4.☐ 9.☐<br>2. ☐...... 1.☐ 2.☐ 3.☐ 4.☐ 8.☐<br>3. ☐...... 1.☐ 2.☐ 3.☐ 4.☐ 9.☐<br>4. ☐...... 1.☐ 2.☐ 3.☐ 4.☐ 9.☐<br>5. ☐...... 1.☐ 2.☐ 3.☐ 4.☐ 9.☐<br>________________________
S5a.6	کیا ماں نے بچے کو کبھی دودھ پلایا؟	1. جی ہاں ☐<br>2. جی نہیں ☐ → S5a.7<br>99. معلوم نہیں ☐ → S5a.7	
S5a.6.1	پیدائش کے کتنے عرصے بعد بچے کو ماں کا دودھ ملا؟		___ ___ دن (DK = 99)<br>یا<br>___ ___ گھنٹے (DK = 99)
S5a.6.2	جب بچے کی بیماری شروع ہوئی، کیا اس وقت بچہ ماں کے دودھ پر تھا؟	1. جی ہاں ☐<br>2. جی نہیں ☐<br>99. معلوم نہیں ☐	
S5a.7	جب بچہ بیمار ہوا، اس وقت اس کی غزا میں کیا شامل تھا؟<br>[Multiple answers allowed.]	1. پاؤڈر دودھ ☐<br>2. گائے کا یا بکری کا دودھ ☐<br>3. جوس ☐<br>4. ORS ☐<br>5. وٹامن کا شربت ☐<br>6. دلیا یا چاول ☐<br>7. کچھ نہیں، صرف ماں کا دودھ ☐<br>99. معلوم نہیں ☐	
V3.11	کیا بچہ پہلے دن چوسنے کی صلاحیت رکھتا تھا؟	1. جی ہاں ☐ → V3.13<br>2. جی نہیں ☐<br>99. معلوم نہیں ☐	
V3.12	کیا بچہ کبھی کسی چیز کو نارمل طریقے سے چوس سکا؟	1. جی ہاں ☐<br>2. جی نہیں ☐ → V3.17<br>99. معلوم نہیں ☐ → V3.17	
V3.13	کیا بچے نے نارمل طریقے سے چوسنا ایک دم چھوڑ دیا؟	1. جی ہاں ☐<br>2. جی نہیں ☐ → V3.17<br>99. معلوم نہیں ☐ → V3.17	
V3.14	پیدائش کے کتنے عرصے بعد، بچے نے نارمل طریقے سے چوسنا چھوڑ دیا؟		___ ___ دن<br>(DK = 99)

بچوں کی اموات پر تحقیق کا سوالنامہ

فارم نمبر [ ][ ][ ][ ] - [ ][ ][ ] - [ ][ ]

کچی آبادی نمبر | گھر نمبر | بچہ نمبر

V3.15	وفات سے کتنے عرصے پہلے بچے نے نارمل طریقے سے چوسنا چھوڑ دیا؟	□1- ایک دن سے کم عرصہ<br>□ 2- ایک دن یا اس سے زیادہ<br>□99- معلوم نہیں	
V3.16	جب بچے نے چوسنا چھوڑا، کیا اس وقت بچہ منہ کھولنے کے قابل تھا؟	□1- جی ہاں<br>□2- جی نہیں<br>□99- معلوم نہیں	
V3.17	کیا بیماری، (جس کی وجہ سے بچہ کی وفات ہوئی) کے دوران بچہ کو سانس کی تکلیف تھی؟	□1- جی ہاں<br>□2- جی نہیں → *V3.20*<br>□99- معلوم نہیں → *V3.20*	
V3.18	بچہ کتنے دن کا تھا جب اس کو یہ سانس کی تکلیف شروع ہوئی؟		___ ___ دن<br>*(DK = 99)*
V3.19	کتنے دن بچہ کو یہ سانس کی تکلیف رہی؟		___ ___ دن<br>*(DK = 99)*
V3.20	کیا بیماری، (جس کی وجہ سے بچہ کی وفات ہوئی) کے دوران بچہ کو تیز سانس چلی؟	□1- جی ہاں → *V3.21*<br>□2- جی نہیں → *V3.23*<br>□99- معلوم نہیں → *V3.23*	
V3.21	بچہ کتنے دن کا تھا جب اس کو تیز سانس چلنا شروع ہوئی؟		___ ___ دن<br>*(DK = 99)*
V3.22	کتنے دن بچے کو تیز سانس چلی؟		___ ___ دن<br>*(DK = 99)*
V3.23	کیا بیماری، (جس کی وجہ سے بچہ کی وفات ہوئی) کے دوران بچہ کی پسلیاں چلی؟	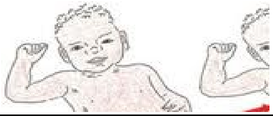	
V3.24	کیا بیماری، (جس کی وجہ سے بچہ کی وفات ہوئی) کے دوران بچہ کی سانس میں آواز تھی؟	□1- جی ہاں<br>□2- جی نہیں<br>□99- معلوم نہیں	
V3.25	کیا بیماری، (جس کی وجہ سے بچہ کی وفات ہوئی) کے دوران بچہ کو مرگی یا جھٹکے کی تکلیف ہوئی؟	□1- جی ہاں<br>□2- جی نہیں<br>□99- معلوم نہیں	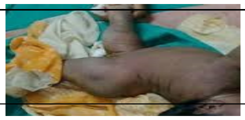
V3.26	کیا بیماری، (جس کی وجہ سے بچہ کی وفات ہوئی) کے دوران بچہ کو بخار تھا؟	□1- جی ہاں<br>□2- جی نہیں → *V3.29*<br>□99- معلوم نہیں → *V3.29*	
V3.27	کس عمر میں بخار شروع ہوا؟		___ ___ دن<br>*(DK = 99)*
V3.28	بخار کتنے عرصے چلا؟		___ ___ دن<br>*(DK = 99)*
V3.29	کیا بیماری، (جس کی وجہ سے بچہ کی وفات ہوئی) کے دوران بچہ کی جلد ٹھنڈی پڑی؟	□1- جی ہاں → *V3.30*<br>□2- جی نہیں → *V3.32*<br>□99- معلوم نہیں → *V3.32*	
V3.30	بچہ کتنے دن کا تھا جب اس کی جلد ٹھنڈی پڑی؟		___ ___ دن<br>*(DK = 99)*
V3.31	کتنے دن بچے کی جلد ٹھنڈی پڑی رہی؟		___ ___ دن<br>*(DK = 99)*
V3.32	کیا بیماری، (جس کی وجہ سے بچہ کی وفات ہوئی) کے دوران بچہ کبھی سست پڑا؟	□1- جی ہاں<br>□2- جی نہیں<br>□99- معلوم نہیں	

بچوں کی اموات پر تحقیق کا سوالنامہ

فارم نمبر | □□□□-□□□-□□

کچی آبادی نمبر | گھر نمبر | بچہ نمبر

V3.33	کیا بیماری، (جس کی وجہ سے بچہ کی وفات ہوئی) کے دوران بچہ کبھی بیہوش ہوا؟	□1. جی ہاں<br>□2. جی نہیں<br>□99. معلوم نہیں	
V3.34	کیا بیماری، (جس کی وجہ سے بچہ کی وفات ہوئی) کے دوران بچے کے سر کا گڑھا کبھی ابھرا ہوا رہا؟	□1. جی ہاں<br>□2. جی نہیں<br>□99. معلوم نہیں	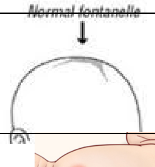
V3.35	کیا بیماری، (جس کی وجہ سے بچہ کی وفات ہوئی) کے دوران بچہ کی ناف سے کبھی پیپ آئی؟	□1. جی ہاں<br>□2. جی نہیں<br>□99. معلوم نہیں	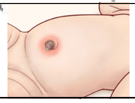
V3.36	کیا بیماری، (جس کی وجہ سے بچہ کی وفات ہوئی) کے دوران بچہ کی ناف کبھی لال پڑی؟	□1. جی ہاں → *V3.37*<br>□2. جی نہیں → *V3.38*<br>□99. معلوم نہیں → *V3.38*	
V3.37	کیا بیماری، (جس کی وجہ سے بچہ کی وفات ہوئی) کے دوران بچہ کی ناف کی لالی بڑھ کر کبھی پیٹ تک آئی؟	□1. جی ہاں<br>□2. جی نہیں<br>□99. معلوم نہیں	
V3.38	کیا بیماری، (جس کی وجہ سے بچہ کی وفات ہوئی) کے دوران بچہ کی جلد پر پیپ بھرے پھوڑے تھے؟	□1. جی ہاں<br>□2. جی نہیں<br>□99. معلوم نہیں	
V3.39	کیا بیماری، (جس کی وجہ سے بچہ کی وفات ہوئی) کے دوران بچہ کی جلد پر چھالے تھے؟	□1. جی ہاں<br>□2. جی نہیں<br>□99. معلوم نہیں	
V3.40	کیا بیماری، (جس کی وجہ سے بچہ کی وفات ہوئی) کے دوران بچہ کی جلد لال اور سوجھی ہوئی تھی؟	□1. جی ہاں<br>□2. جی نہیں<br>□99. معلوم نہیں	
V3.41	کیا بیماری، (جس کی وجہ سے بچہ کی وفات ہوئی) کے دوران بچہ کی جلد کہیں سے کالی پڑی؟	□1. جی ہاں<br>□2. جی نہیں<br>□99. معلوم نہیں	
V3.42	کیا بیماری، (جس کی وجہ سے بچہ کی وفات ہوئی) کے دوران بچہ کے جسم کے کسی حصے سے خون بہا؟	□1. جی ہاں → *V3.43*<br>□2. جی نہیں → *V3.44*<br>□99. معلوم نہیں → *V3.44*	
V3.43	وہ کونسی جگہ تھی جہاں سے خون بہا؟	________________<br>________________	
V3.44	کیا بیماری، (جس کی وجہ سے بچہ کی وفات ہوئی) کے دوران بچہ کو پتلے جلاب یا دست آئے؟	□1. جی ہاں<br>□2. جی نہیں → *V3.46*<br>□99. معلوم نہیں → *V3.46*	
V3.45	جس دن سب سے زیادہ پتلے جلاب ہوئے، اس دن کل کتنی بار بچہ کو پتلے جلاب آئے؟		پتلے جلاب __ __<br>*(DK = 99)*
V3.46	کیا بیماری، (جس کی وجہ سے بچہ کی وفات ہوئی) کے دوران بچہ کو الٹی ہوئی؟	□1. جی ہاں<br>□2. جی نہیں<br>□99. معلوم نہیں	
V3.47	کیا بیماری، (جس کی وجہ سے بچہ کی وفات ہوئی) کے دوران بچہ کی جلد پیلی پڑی؟	□1. جی ہاں<br>□2. جی نہیں<br>□99. معلوم نہیں	
V3.48	کیا بیماری، (جس کی وجہ سے بچہ کی وفات ہوئی) کے دوران بچہ کی آنکھیں پیلی پڑیں؟	□1. جی ہاں<br>□2. جی نہیں<br>□99. معلوم نہیں	
V3.49	کیا بچہ صحت مند تھا اور فوراً وفات پا گیا؟	□1. جی ہاں<br>□2. جی نہیں<br>□99. معلوم نہیں	
**S5a.8**	1. اگر بچہ مرکز میں پیدا ہوا → ***S5a.8.1***	2. اگر بچہ مرکز میں نہیں پیدا ہوا → ***S5a.10***	**99.** معلوم نہیں → ***S5a.1***
S5a.8.1	کیا بچہ پیدا کئے گئے مرکز سے زندہ باہر آیا، یا پھر اس کی وفات مرکز ہی میں ہوئی؟	1. ہاں، زندہ باہر آیا → ***S5a.8.2***<br>2. مرکز میں وفات پائی → ***S6.1***<br>9. معلوم نہیں → ***S6.1***	□

بچوں کی اموات پر تحقیق کا سوالنامہ

فارم نمبر ☐☐☐☐-☐☐☐-☐☐

کچی آبادی نمبر گھر نمبر بچہ نمبر

S5a.8.2	پیدا ہونے کے کتنے عرصے بعد، بچہ مرکز سے باہر لے جایا گیا؟		دن ___ ___<br>(DK = 99)<br>یا<br>گھنٹے ___ ___<br>(DK = 99)
S5a.8.3	کیا اسپتال سے چھٹی سے پہلے، بچہ کا مکمل معائنہ ہوا؟	☐1- جی ہاں<br>☐2- جی نہیں<br>☐99- معلوم نہیں	
S5a.9	کیا چھٹی کے وقت، مرکز نے ماں کو مکمل ہدایات مہیا کیں؟	☐1- جی ہاں → S5a.9.1<br>☐2- جی نہیں → S5a.10<br>☐99- معلوم نہیں → S5a.10	
S5a.9.1	وہ ہدایات کن باتوں کے بارے میں تھیں؟	1. ماں کا دودھ ☐<br>2. حفا ظتی ٹیکے ☐<br>3. پیدائش کے بعد مرکز میں حاضری ☐<br>4. نومولود بیماریون کی خطرے کی علامات ☐<br>5. دیگر (specify) ☐<br>99. معلوم نہیں ☐	1. ☐<br>2. ☐<br>3. ☐<br>4. ☐<br>5. ☐ ____________<br>9. ☐
S5a.10	کیا بیماری، (جس کی وجہ سے بچہ کی وفات ہوئی) سے پہلے بچے کو کبھی ان میں سے کسی شخص کو دکھایا گیا؟<br><br>اگر بیماری سے پہلے دکھایا گیا، تو کتنی بار دکھایا گیا؟<br><br>بچے کی کتنی عمر تھی (دنوں میں) جب بچے کو بیماری سب سے پہلے بیماری سے پہلے دکھایا گیا؟	1- گھر میں یا باہر علاقے کی نرس کو دکھایا<br>2- ڈاکٹر یا نرس کہ (مرکز میں)<br>3- کسی کو نہیں دکھایا گیا<br>99- معلوم نہیں	**دکھایا گیا** / **کتنی بار** / **پہلا وزٹ**<br>1. ☐... .....___ ___<br>2. ☐... .....___ ___<br>3. ☐<br>9. ☐<br>___ ___ دن<br>(<1 = 00; DK = 99)
S5a.11	کیا بیماری، (جس کی وجہ سے بچہ کی وفات ہوئی) سے پہلے بچے کو ان میں سے کوئی بیماری تھی؟<br><br>اگر ان میں سے کوئی بیماری تھی، تو کیا اس کا علاج کروایا؟	1. قبل از وقت ....<br>a. کیا بچہ کو خصوصی عذا مہیا کی گئ؟?....<br>b. کیا بچہ کو "کینگرو کیر" مییا کی گئ<br>2. (پیدائشی) جسمانی ترتیبی<br>a. سر، گردن یا پیٹھ میں ....<br>b. منہ کے تالو میں ....<br>c. دل..<br>d. ہاتھ یا پاؤں میں.............................<br><br>3. دیگر	**میں مبتلا ہوا** (Yes No DK) / **علاج کروایا** (Yes No DK)<br>1. ☐ 2. ☐ 9. ☐<br>............................ / 1. ☐ 2. ☐ 9. ☐<br>............................ / 1. ☐ 2. ☐ 9. ☐<br>1. ☐ 2. ☐ 9. ☐ / 1. ☐ 2. ☐ 9. ☐<br>1. ☐ 2. ☐ 9. ☐ / 1. ☐ 2. ☐ 9. ☐<br>1. ☐ 2. ☐ 9. ☐ / 1. ☐ 2. ☐ 9. ☐<br>1. ☐ 2. ☐ 9. ☐ / 1. ☐ 2. ☐ 9. ☐<br>1. ☐ 2. ☐ 9. ☐ / 1. ☐ 2. ☐ 9. ☐<br>____________________

فارم نمبر | کچی آبادی نمبر | گھر نمبر | بچہ نمبر

بچوں کی اموات پر تحقیق کا سوالنامہ

SA Module 5b: بڑے بچوں (شیر خوار سے بڑے) کی بیماریوں سے بچاؤ کی معلومات

پڑھیئے: اب میں آپ سے مرحوم بچے کی بیماری (جسکی وجہ سے موت واقع ہوئ) سے پہلے آپکی طرف سے اختیار کئے گئے حفاظتی اقدامات کے بارے میں کچھ بنیادی معلومات حاصل کرنا چاہتا ہوں۔

S5b.1	آپ (ماں) کہاں کھانا پکاتی ہیں؟	1. گھر کے اندر<br>2. گھر کے باہر<br>3. گھر کے باہر (کمرے میں)<br>99. معلوم نہیں	☐
S5b.2	کیا ماں کھانا پکاتے وقت مرحوم بچے کو اپنے ساتھ رکھتی تھی؟	☐1. جی ہاں<br>☐2. جی نہیں<br>☐99. معلوم نہیں	☐
S5b.3	اس خطرناک بیماری میں مبتلا ہونے سے پہلے کیا مرحوم بچہ مچھر دانی کے نیچے سوتا تھا؟	☐1. جی ہاں<br>☐2. جی نہیں<br>☐ 3. جی ہاں، کبھی کبھار<br>☐99. معلوم نہیں	☐
S5b.4	کیا ماں (یا دودھ پلانے والی دائی) نے کبھی مرحوم بچہ کو دودھ پلایا؟	→ S5b.5<br>→ S5b.5	☐
S5b.4.1	جب یہ خطرناک بیماری شروع ہوی تو کیا مرحوم بچہ ماں یا دائی کا دودھ پیتا تھا؟	Yes ☐1. جی ہاں<br>No ☐2. جی نہیں → S5b.5<br>9. Don't know ☐99. معلوم نہیں → S5b.5	☐
S5b.4.2	جب بچے نے ماں یا دائی کا دودھ پینا شروع کیا تو اس وقت بچے کی کتنی عمر تھی؟		مہینے ___ ___<br>(<1 = 00; DK = 99)
S5b.5	جب یہ خطرناک بیماری سروع ہوی، تو اس وقت کیا مرحوم بچہ کو ماں کے دودھ کے علاوہ کوئی اور چیز دی جاتی تھی؟	1. پاؤڈر کا دودھ<br>2. گائے/ بکری کا دودھ<br>3. جوس / پانی<br>4. او-آر – ایس<br>5. وٹامن کے قطرے → S5b.6<br>6. چاول، گیہوں، گندم (نشاستہ دار اشیاء)<br>7. کچھ نہیں، صرف ماں کا دودھ<br>99. معلوم نہیں → S5b.6	
S5b.5.1	یہ بیماری شرح ہونے سے پہلے، بچہ عام طور پر (ایک دن میں) کتنی بار نشاستہ دار اشیاء (پانی یا دودھ کے علاوہ) کھاتا تھا؟		دفعا ___ ___<br>(DK = 99)
S5b.5.2	ان میں سے کونسی اشیاء بچہ اکثر کھایا کرتا تھا؟	دلیا/گیہوں/چاول پھلیاں<br>دودھ/دھی<br>گوشت<br>انڈہ<br>وٹامن A والی اشیاء<br>سبزی/پھل	

اب میں آپ سے بچے کے حفاظتی ٹیکوں کے کورس کے بارے میں معلومات حاصل کرنا چاہتا ہوں:

S5b.7	کیا آپکے پاس حفاظتی ٹیکوں کا کارڈ موجود ہے؟ اگر 'ہاں'، تو کیا مجھے دکھا سکتے ہیں؟	☐1. کارڈ ہے (اور دکھا بھی دیا)<br>☐2. کارڈ ہے (مگر دکھا نہیں سکا) → S5b.8<br>☐ 99. کارڈ نہیں ہے → S5b.8	☐

بچوں کی اموات پر تحقیق کا سوالنامہ

فارم نمبر | | | | | - | | | | - | | |

کچی آبادی نمبر — گھر نمبر — بچہ نمبر

	کارڈ پر موجود تاریخ کا اندراج یہاں فارم پر کریں:<br>اگر کارڈ پر تاریخ مقجود نہیں ہے تہ 99999999 لکہیں	BCG ....<br>POLIO 0 (given at birth)..<br>POLIO 1 ....<br>POLIO 2 ....<br>POLIO 3 ....<br>DPT 1 ....<br>DPT 2 ....<br>DPT 3 ....<br>MEASLES ....<br>HEPATITIS B1 ....<br>HEPATITIS B2 ....<br>HEPATITIS B3	دن / مہینا / سال	BCG<br>P0<br>P1<br>P2<br>P3<br>DPT1<br>DPT2<br>DPT2<br>MSL<br>HEP1<br>HEP2<br>HEP3
S5b.7.1	کیا بچہ کو ایسی ویکسین (ٹیکا) بھی لگوایا گو اس کارڈ میں موجود نہیں ہے؟	□1. جی ھاں<br>□2. جی نھیں<br>□99. معلوم نھیں		
S5b.8	کیا بچے کو عام ٹیکے کے علاوہ کوئی اور ٹیکا لگوایا؟	□1. جی ھاں<br>□2. جی نھیں<br>□99. معلوم نھیں	□ 2 or 9 → S5b.10	
.1	کیا بی سی جی کا ٹیکا لگوایا گیا؟	□1. جی ھاں<br>□2. جی نھیں<br>□99. معلوم نھیں	□	
.2	کیا پولیو کے قطرے بچہ کو پلائے گئے؟	□1. جی ھاں<br>□2. جی نھیں<br>□99. معلوم نھیں	□ 2 or 9 → S5b.8.5	
.3	پولیو کا ٹیکا کب لگا؟	1. پیدائش کے ٹھیک بعد<br>2. کچھ دیر بعد<br>99. معلوم نہیں	□	
.4	پولیو کیا ٹیکا کتنی دفعا لگوایا جیا؟		___ ___ Times<br>*(DK = 99)*	
.5	کیا ڈی پی ٹی کا انجکشن لگوایا گیا؟	□1. جی ھاں<br>□2. جی نھیں<br>□99. معلوم نھیں	□ 2 or 9 → S5b.8.7	
.6	کتنی دفعا؟		___ ___ دفعا<br>*(DK = 99)*	
.7	ایم ایم آر کا ٹیکا لگا؟	□1. جی ھاں<br>□2. جی نھیں<br>□99. معلوم نھیں	□	

بچوں کی اموات پر تحقیق کا سوالنامہ

فارم نمبر | | | | | - | | | | - | | |

کچی آبادی نمبر   گھر نمبر   بچہ نمبر

-8	ہیپاٹائٹس بی کا ٹیکا لگوایا؟	□1. جی ہاں<br>□2. جی نہیں<br>□99. معلوم نہیں	
-9	کتنی دفعا یہ ٹیکا لگویا تھا؟		________ دفعا
S5b.9	کوئی اور ٹیکا لگوایا؟	□1. جی ہاں<br>□2. جی نہیں<br>□99. معلوم نہیں	
S5b.9.1	یہ کیمپین کس تاریخ کو ہوئی؟		
S5b.10	اس خطرناک بیماری سے 6 مہینے پہلے، کیا بچے کو وٹامن-اے دیا گیا تھا؟ (قطرے، کیپسول، شربت وغیرہ)<br>(صرف ایک جواب)	1۔ ہاں (ایک بار)<br>2۔ ہاں (2 بار یا زیادہ)<br>3۔ نہیں<br>99۔ معلوم نہیں	□
S5b.11	یہ خطرناک بیماری شروع ہونے سے پہلے کیا بچہ کو ان میں سے کسی بیماری؟ کا سامنا کرنا پڑا؟<br>(ایک سے زیادہ جوابات)	1- قد یا وزن میں کمی ہوئ<br>2- پیدائیشی جسمانی نقص<br>- سر، گردن یا پیٹھ پر<br>- منہ یا تال پر<br>- دل میں<br>- بانھوں یا پیروں میں<br>3- دمہ (سانس کی پیدائشی تکلیف)<br>4- دل کی بیماری<br>5- ٹی بی<br>6- مرگھی (یا جھٹکے کی بیماری)<br>7-ایڈز<br>8- دیگر (آگے وضاحت کریں)<br><br>(specify other)...................................	مبتلا ہوا؟<br>ہاں نہیں معلوم نہیں<br>1. □ 2. □ 9. □<br>1. □ 2. □ 9. □<br>1. □ 2. □ 9. □<br>1. □ 2. □ 9. □<br>1. □ 2. □ 9. □<br>1. □ 2. □ 9. □<br>1. □ 2. □ 9. □<br>1. □ 2. □ 9. □<br>1. □ 2. □ 9. □<br>1. □ 2. □ 9. □<br>1. □ 2. □ 9. □<br><br>مبتلا ہوا؟<br>ہاں نہیں معلوم نہیں<br>1. □ 2. □ 9. □<br>1. □ 2. □ 9. □<br>1. □ 2. □ 9. □<br>1. □ 2. □ 9. □<br>1. □ 2. □ 9. □<br>1. □ 2. □ 9. □<br>1. □ 2. □ 9. □<br>1. □ 2. □ 9. □<br>1. □ 2. □ 9. □<br>1. □ 2. □ 9. □<br>1. □ 2. □ 9. □<br><br>________________________

VA Section 4 بڑے بچوں کی اموات کی بنیادی معلومات

اب میں مرحوم بچے کی اس خطرناک بیماری کے بارے میں کچھ سوالات حاصل کرنا چاہتا ہوں۔

V4.1	اس خطرناک بیماری کے دوران کیا بچے کو بخار تھا؟<br>(صرف ایک جواب)	□1. جی ہاں<br>□2. جی نہیں<br>□99. معلوم نہیں	□ *2 or 9 → V4.6*
V4.2	اس خطرناک بیماری دوران بچے کو بخار کتنے دن رہا؟		— — دن<br>*(DK = 99)*
V4.3	کیا یہ بخار بچے کی وفات تک جاری رہا؟ (صرف ایک جواب)	□1. جی ہاں<br>□2. جی نہیں<br>□99. معلوم نہیں	□ *2 or 9 → V4.6*
V4.4	بخار کتنا تیز تھا؟ (صرف ایک جواب)	1. ہلکا سا<br>2. درمیانہ سا<br>3. تیز<br>99- معلوم نہیں	□
V4.5	کیا بخار مطواتر چلا یا کم بھی ہو تا تھا؟<br>(صرف ایک جواب)	1. مسلسل اور مطواتر چلا<br>2. غیر مطواتر<br>3. صرف رات کو<br>99. معلوم نہیں	□

فارم نمبر | | | | | - | | | | - | | |
کچی آبادی نمبر — گھر نمبر — بچہ نمبر

بچوں کی اموات پر تحقیق کا سوالنامہ

V4.6	کیا بیماری جس کی وجہ سے موت ہوئی، کے دوران بچہ کو کثرت سے دست ہوئے تھے؟	□1. جی ھاں<br>□2. جی نہیں<br>□99. معلوم نہیں	□ 2 or 9 → V4.12
V4.7	اس بیماری کے دوران، جس دن بچہ کو کثرت کے ساتھ دست ہوئے تھے، اس دن کل کتنی بار بچہ کو دست ہوئے؟		__ __ دست<br>(DK = 99)
V4.8	وفات سے کتنے دن پہلے بچہ کو کثرت سے دست آن شروع ہوئے؟		__ __ دن<br>(DK = 99)
V4.9	کیا کثرت سے دست کی شکایت، بچہ کی وفات تک جاری رہی؟	□1. جی ھاں<br>□2. جی نہیں<br>□99. معلوم نہیں	□ 1 or 9 → V4.11
V4.10	وفات سے کتنے دن پہلے دست رکے تھے؟		__ __ دن<br>(DK = 99)
V4.11	کیا ڈھیلے یا مائع پاخانہ میں خون نظر آتا تھا؟	□1. جی ھاں<br>□2. جی نہیں<br>□99. معلوم نہیں	□
V4.12	بیماری کے دوران جو موت کا باعث بنی، کیا بچے کو کھانسی ہوئی؟	□1. جی ھاں<br>□2. جی نہیں<br>□99. معلوم نہیں	□ 2 or 9 → V4.16
V4.13	کھانسی کتنے دنوں تک رہی؟<br>[24 گھنٹے سے کم="00" دن]		__ __ دن<br>(DK = 99)
V4.14	کیا کھانسی بہت شدید تھی؟	□1. جی ھاں<br>□2. جی نہیں<br>□99. معلوم نہیں	□
V4.15	کیا بچہ کھانسی کے بعد قے کرتا ہے؟	□1. جی ھاں<br>□2. جی نہیں<br>□99. معلوم نہیں	□
V4.16	بیماری کے دوران جو موت کا باعث بنی، کیا بچے کو سانس لینے میں دشواری تھی؟	□1. جی ھاں<br>□2. جی نہیں<br>□99. معلوم نہیں	□ 2 or 9 → V4.18
V4.17	سانس کی مشکل کتنے دنون تک جاری رہی؟		__ __ دن<br>(DK = 99)
V4.18	بیماری کے دوران جو موت کا باعث بنی، کیا بچے کو تیز سانس چلنے کا مسئلہ ہوا؟	□1. جی ھاں<br>□2. جی نہیں<br>□99. معلوم نہیں	□ 2 or 9 → V4.20
V4.19	تیز سانس کتنے دنوں تک چلی؟		__ __ دن<br>(DK = 99)
V4.20	بیماری کے دوران جو موت کا باعث بنی، کیا بچے کو تیز پسلیاں چلنے کا مسئلہ ہوا؟	□1. جی ھاں<br>□2. جی نہیں<br>□99. معلوم نہیں	□
V4.21	بیماری کے دوران جو موت کا باعث بنی، کیا بچے کی سانس میں آوازیں آئیں؟		
V4.22	اسٹرائڈر	□1. جی ھاں<br>□2. جی نہیں<br>□99. معلوم نہیں	□
V4.23	کراہنا	□1. جی ھاں<br>□2. جی نہیں<br>□99. معلوم نہیں	□

بچوں کی اموات پر تحقیق کا سوالنامہ

فارم نمبر [ ][ ][ ][ ]-[ ][ ][ ]-[ ][ ]

کچی آبادی نمبر — گھر نمبر — بچہ نمبر

V4.24	گھر گھراہٹ	□1. جی هاں<br>□2. جی نہیں<br>□99. معلوم نہیں	☐
V4.25	بیماری کے دوران جو موت کا باعث بنی، کیا بچے کو جھٹکے لگے؟	□1. جی هاں<br>□2. جی نہیں<br>□99. معلوم نہیں	☐
V4.26	بیماری کے دوران جو موت کا باعث بنی، کیا بچہ بے ہوش ہوا؟	□1. جی هاں<br>□2. جی نہیں<br>□99. معلوم نہیں	☐ *2 or 9 → V4.28*
V4.27	وفات سے پہلے بے ہوشی کتنے عرصے پہلے شروع ہوئی؟	1۔ چھ گھنٹے سے کم<br>2۔6-23 گھنٹوں کے درمیان<br>3۔23 گھنٹوں یا اس سے زیادہ<br>4۔ معلوم نہیں	☐
V4.28	بیماری کے دوران جو موت کا باعث بنی، کیا بچے کی گردن اکڑی تھی؟	□1. جی هاں<br>□2. جی نہیں<br>□99. معلوم نہیں	☐
V4.29	بیماری کے دوران جو موت کا باعث بنی، کیا بچے کے سَر کی ہڈیوں کے دَرمِیان جھِلّی دار حلاِ پھولی تھی؟	□1. جی هاں<br>□2. جی نہیں<br>□99. معلوم نہیں	☐
V4.30	بیماری کے دوران جو موت کا باعث بنی، کیا بچے کی جلد پر خارش ہوئی تھی؟	□1. جی هاں<br>□2. جی نہیں<br>□99. معلوم نہیں	☐ *2 or 9 → V4.35*
V4.31	خارش، جسم کے کس حصے پر تھی؟	1. چہرے پر<br>2. چھاتی یا پیٹ پر<br>3. ہاتھ/پاؤں پر<br>4. ہر جگہ پر<br>9. معلوم نہیں	☐
V4.32	جسم کے کس حصے پر خارش سے شروع ہوئی تھی؟	1. چہرے پر<br>2. چھاتی یا پیٹ پر<br>3. ہاتھ/پاؤں پر<br>4. ہر جگہ پر<br>9. معلوم نہیں	☐
V4.33	خارش کتنے دن جاری رہی؟		دن ____ ____<br>*(DK = 99)*
V4.34	کیا خارش میں پانی کے آبلے ہوئے تھے؟	□1. جی هاں<br>□2. جی نہیں<br>□99. معلوم نہیں	☐
V4.35	بیماری کے دوران جو موت کا باعث بنی، کیا بچے کے ہاتھ یا پاؤں پتلے دبلے ہوئے تھے؟	□1. جی هاں<br>□2. جی نہیں<br>□99. معلوم نہیں	☐
V4.36	بیماری کے دوران جو موت کا باعث بنی، کیا بچے کے ہاتھ یا پاؤں سوجے تھے؟	□1. جی هاں<br>□2. جی نہیں<br>□99. معلوم نہیں	☐ *2 or 9 → V4.38*
V4.37	سوجن کتنے عرصے برقرار رہی؟		دن ____ ____<br>*(DK = 99)*<br>ہفتے ___ ___<br>*(DK = 99)*
V4.38	بیماری کے دوران جو موت کا باعث بنی، کیا بچے کی جلد پھٹی تھی؟	□1. جی هاں<br>□2. جی نہیں<br>□99. معلوم نہیں	☐

بچوں کی اموات پر تحقیق کا سوالنامہ

فارم نمبر ☐☐☐☐-☐☐☐-☐☐

کچی آبادی نمبر — گھر نمبر — بچہ نمبر

V4.39	بیماری کے دوران جو موت کا باعث بنی، کیا بچے کے بالوں کا رنگ سرخ یا زرد رنگ میں تبدیل ہوا تھا؟	☐1. جی ہاں<br>☐2. جی نہیں<br>☐99. معلوم نہیں	
V4.40	بیماری کے دوران جو موت کا باعث بنی، کیا بچے کا پیٹ پھولا تھا؟	☐1. جی ہاں<br>☐2. جی نہیں<br>☐99. معلوم نہیں	
V4.41	بیماری کے دوران جو موت کا باعث بنی، کیا بچے کو خون کی کمی یا اس کی رنگت زرد پڑی؟	☐1. جی ہاں<br>☐2. جی نہیں<br>☐99. معلوم نہیں	
V4.42	بیماری کے دوران جو موت کا باعث بنی، کیا بچے کی بغلوں میں سوجن ہوئی؟	☐1. جی ہاں<br>☐2. جی نہیں<br>☐99. معلوم نہیں	
V4.43	بیماری کے دوران جو موت کا باعث بنی، کیا بچے کا منہ یا زبان اندر سے سفید ہوئی؟	☐1. جی ہاں<br>☐2. جی نہیں<br>☐99. معلوم نہیں	
V4.44	بیماری کے دوران جو موت کا باعث بنی، کیا بچہ کہ جسم کے کسی حصے سے خون نکلا؟	☐1. جی ہاں<br>☐2. جی نہیں<br>☐99. معلوم نہیں	2 or 9 → V4.46
V4.45	بچے کے جسم کے کس حصے سے خون نکلا، سامنے درج کریں۔		
V4.46	بیماری کے دوران جو موت کا باعث بنی، کیا بچہ کے جلد پر کوئ کالا نشان آیا؟	☐1. جی ہاں<br>☐2. جی نہیں<br>☐99. معلوم نہیں	
V4.47	کیا بچہ کے ساتھ کوئ حادثہ پیش آیا یا پھر کوئ چوٹ لگی؟	(see table below)	

	جی ہاں	جی نہیں	معلوم نہیں	
1. روڈ ٹریفک حادثہ /چوٹ۔	☐	☐	☐	
2۔ بچہ اونچای سے گرا تھا۔	☐	☐	☐	اگر ان 8 سوالوں میں سے جواب "جی نہیں' اور "معلوم نہیں" ہے تو، سوال S6.1 پر جائیں۔
3۔ بچہ پانی میں ڈوبا تھا	☐	☐	☐	
4۔ بچہ نے کوئ زہریلی چیز کھائ یا پی تھی	☐	☐	☐	
کیا بچہ :				
5۔ کو کسی زہریلے کیڑے یا جانور نے کاٹا تھا؟	☐	☐	☐	
6۔ آگ میں یا گرم پانی میں جھلسا تھا؟	☐	☐	☐	
7۔ کسی تشدد کا نشانہ بنا تھا؟	☐	☐	☐	
8۔ کوئ اور چوٹ لگی؟ (درج کریں)---------------------	☐	☐	☐	

(If "Yes," then specify)........................... ____________________

V4.48	کیا چوٹ بچہ نے خود اپنے آپ کو لگائ، یا کسی اور نے چوٹ پہنچائ؟	☐1. جی ہاں<br>☐2. جی نہیں<br>☐99. معلوم نہیں	
V4.49	چوٹ لگنے کے کتنے عرصے بعد تک بچہ زندہ رہا؟		گھنٹے — —<br>(DK = 99)<br>دن — —<br>(DK = 99)

بچوں کی اموات پر تحقیق کا سوالنامہ

فارم نمبر ☐☐☐☐-☐☐☐-☐☐

کچی آبادی نمبر　گھر نمبر　بچہ نمبر

SA Module 6: والدین کی (بچہ کی جانلیوہ بیماری کے دوران) صحتیابی حاصل کرنے کے لئے حاصل کی گی مدد کی معلومات

اب میں آپ سے بچہ کی جانلیوہ بیماری اور اس کے دوران آپ کی وہ تمام کوششیں جو آپ نے بچہ کو صحتیاب کرنے کیلیے کریں، کے بارے میں پوچھوں گا/گی۔ : پڑھیں

S6.1	کس نے بچہ کو سب سے پہلے بیمار پایا؟	1۔ جوابدہندہ نے<br>2۔ پڑوسی، رشتہ داریا دوست نے<br>3۔ محلے کی دائی یا نرس نے<br>4۔ ڈاکٹر یا نرس (اسپتال میں) نے<br>5۔ کسی اور نے (درج کریں)۔ ________	☐ ________

S6.2	پہلے آپ نے بتایا تھا کہ بیماری کے دوران، بچہ کو یہ علامات ظاہر ہوئی تھیں (کے آخر میں دی گی علامات کا ذکر کریں)<br>-سوال س6۔1۔ میں ذکر کردہ شخص کو سب سے پہلے یہ کیسے پتا چلا کہ بچہ بیمار ہے؟ اور کونسی علامات بچہ میں اس وقت موجود تھیں؟	**علامات، ظہور کی ترتیب میں**	**بیماری کے کس دن علامات ظاہر ہوئیں؟**
		1.	
		2.	
		3.	
		4	
		5.	
		6.	
		7.	

			نارمل	معمولی	غیر معمولی	معلوم نہیں
S6.3	-سوال س6۔1۔ میں ذکر کردہ شخص نے جب بچے کو بیمار پایا، تو اس وقت بچے کی کیا حالت تھی؟	1۔ اچھی طرح کھا، پی رہا تھا/ کم کھا، پی رہا تھا/ بالکل کھا، پی نہیں رہا تھا/ معلوم نہیں				
		2۔ بالکل ہوش میں تھا/ تھوڑا ہوش کھو چکا تھا/ بے ہوش تھا/ معلوم نہیں				
		3۔ بالکل چاقو چوبند تھا/ معمول سے کم چاقو چوبند تھا/ بالکل نہیں ہل رہا تھا/ معلوم نہیں				

S6.4	کیا آپ نے بچے کیا س مہلک بیماری کے لئے کوئی دیکھ بھال یا علاج حاصل کیا، یا تلاش کرنے کی کوشش کی؟	1۔ جی ہاں<br>2۔ نہیں (ضرورت نہیں تھی/ کوشش ہی نہیں کری)<br>3۔ نہیں (بچہ فورن ہی مر گیا)<br>4۔ معلوم نہیں	☐ 2 → S6.6<br>3 or 9 → V5.10

S6.5

ہمں ان تمام اقدامات (یا تو گھر پر یا پھر گھر سے باہر) سے آگاہ کریں جو آپ نے بچے کی خطرناک بیماری سے نمٹنے کیلئے اٹھائے۔ پہلے، پھر دوسرے اور اسطرح تمام اقدامات کا باری باری ذکر کریں۔

اس ادارے کا ذکر بھی کریں جہاں آپ بچہ / بچی کو نہیں لے جاسکے، کیونکہ بچہ راستے ہی میں دم توڑ گیا۔

(1) ہر اقدام کیلیے ٹک کریں، "دوسری مدد" اور "صحت فراہم کرنے والے ادارے"۔ (2) چار ہفتے سے کم بچوں کیلیۓ

اقدام نمبر #	(1) دوسری مدد: گھر پر مدد کری (اپنے رشتے دار/ پڑوسی/ اپنے دوست)	(1) دوسری مدد: روایتی یا غیر رسمی فراہم کنندہ۔	(1) دوسری مدد: دوا بیچنے والوں سے مدد حاصل کری	(1) صحت فراہم کرنے والے ادارے: تربیت یافتہ نرس یا مڈوائف سے مدد حاصل کی/ معلوم نہیں	(1) صحت فراہم کرنے والے ادارے: پرائیویٹ ڈاکٹر سے مدد حاصل کی	(1) صحت فراہم کرنے والے ادارے: این جی او یا گورنمنٹ کلینک سے مدد حاصل کی	(1) صحت فراہم کرنے والے ادارے: اسپتال سے مدد حاصل کی	(2) بیماری، صحت فراہم کرنے والے ادارے (جہاں پر بچہ پیدا ہوا) میں شروع ہوئی	(3) بیماری کا وہ کونسا دن تھا، جس دن یہ قدم اٹھایا۔	(4) وہ کونسی علامت تھی جس کے لےۓ یہ قدم اٹھایا؟
1.	☐	☐	☐	☐	☐	☐	☐	☐	__ __ (99 = معلوم نہیں)	
2.	☐	☐	☐	☐	☐	☐	☐		__ __ (99 = معلوم نہیں)	
3.	☐	☐	☐	☐	☐	☐	☐		__ __ (99 = معلوم نہیں)	

بچوں کی اموات پر تحقیق کا سوالنامہ

فارم نمبر ☐☐☐☐-☐☐☐-☐☐

کچی آبادی نمبر | گھر نمبر | بچہ نمبر

4.	☐	☐	☐	☐	☐	☐	☐		__ __ (99 = معلوم نہیں)	
5.	☐	☐	☐	☐	☐	☐	☐		__ __ (99 = معلوم نہیں)	
6.	☐	☐	☐	☐	☐	☐	☐		__ __ (99 = معلوم نہیں)	
7.	☐	☐	☐	☐	☐	☐	☐		__ __ (99 = معلوم نہیں)	

S6.6	اگر کوئی مدد نہیں دی گئی، تو یہ کس نے فیصلہ کیا کہ بچے کو کوئی مدد کو ضرورت نہیں ہے؟<br><br>اگر کوئی مدد دی گئی، تو یہ کس نے فیصلہ کیا کہ (ایکشن-1) میں درج کئے گئے عمل کی ضرورت ہے؟	1. بچے کی والدہ.......................................<br>2. بچے کا والد..<br>3. بچے کی خالہ / پھوپھو...<br>4. بچے کے ماموں/چچا..<br>5. بچے کی نانی.............................<br>6. بچے کے دادا..................<br>7. بچے کے نانا..................<br>8. دیگر (وضاحت کریں).......................................<br>9. معلوم نہیں	1. ☐<br>2. ☐<br>3. ☐<br>4. ☐<br>5. ☐<br>6. ☐<br>7. ☐<br>8. ☐ ____________<br>9. ☐
S6.7	اگر آپ کسی صحت کے ادارے میں بچے کو لے کر نہیں گئے، تو آپ کو کوئی مسئلہ تھا جس کی وجہ سے بچے کوئی سہولت نہیں دی گئی؟<br><br>اگر آپ بچے کو کسی صحت کے ادارے میں لے کر گئے، تو آپ کن مسائل کا سامنا کر کے، بچے کو سہولت دے سکے؟	1. جی ہاں<br>2. نہیں<br>9. معلوم نہیں	☐ 2 or 9 → Inst_2
S6.7.1	آپ کو کیا مسائل تھے؟<br>(ایک سے زیادہ جواب ہو سکتے ہیں)	1۔ میں یہ سمجھتا تھا کہ بچے کے صحت اتنی خراب نہیں ہے اور اس کو کہیں لے جانے کو ضرورت نہیں۔<br>2۔ بچے کو لے جانے لیلئے کوئی گھر پر موجود نہیں تھا۔<br>3۔ میں باہر کے کاموں میں بہت مصروف تھا۔<br>4۔ کسے اور نے اس بات کا فیصلہ کرنا تھا۔<br>5۔ سفر بیت لمبا تھا۔<br>6۔ کوئی گاڑی موجود نہیں تھی۔<br>7۔ پیسوں کا مسئلہ تھا۔<br>8۔ میں صحت کے مرکز پر موجود صہولیات پر مطمئن نہیں تھا۔<br>9۔ میں یہ سمجھتا تھا کہ روایتی علاج سے صحیح ہو جائے گا۔<br>10۔ میں یہ سمجھتا تھا کہ بچہ کی صحت سفر کیلیئے بہت ناموزوں ہے۔<br>11۔ میں یہ سمجھتا تھا کہ بچہ علاج کے باوجود نہیں بچ سکتا۔<br>12۔ بہت رات ہو چکی تھی اور کوئی گاڑی موجود نہیں تھی۔<br>13۔ دیگر (وضاحت کریں)<br>99۔ معلوم نہیں	1. ☐<br>2. ☐<br>3. ☐<br>4. ☐ ____________<br>5. ☐<br>6. ☐<br>7. ☐<br>8. ☐<br>9. ☐<br>10. ☐<br>11. ☐<br>12. ☐<br>13. ☐ ____________<br>99. ☐

اگر سوال ایس کیو4۔6۔ میں آپ نے (2۔ نہیں (ضرورت نہیں تھی / کوشش ہی نہیں کری) کو درج کیا تھا، یا ایس کیو5۔6 میں آپ نے یہ بتایا ہے کہ آپ بچے کو کسی صحت کے مرکز میں نہیں لے گئے، تو سوال نمبر ایس کیو9۔3 میں ۔

S6.8		__ __ دن<br>(DK = 99)<br><br>__ __ گھنٹے<br>(DK = 99)<br><br>__ __ منٹ<br>(DK = 99)

بچوں کی اموات پر تحقیق کا سوالنامہ

فارم نمبر ☐☐☐☐ - ☐☐☐ - ☐☐

کچی آبادی نمبر | گھر نمبر | بچہ نمبر

– بچے کی بیماری کے سوالات –

سوال	جوابات	پہلا صحت کا مرکز	آخری صحت کا مرکز
جب اس باتک کا فیصلہ کیا گیا کہ بچے کو (پہلے / آخری) مرکز صحت لے جانا ہے تو کیا بچہ:	1۔ کھانا نارمل کھارھا تھا / کم کھارھا تھا / بالکل نہیں۔ کھارھا تھا<br>2۔ ہشاش بشاش تھا / ھلکا بے ہوش تھا / بے ہوش تھا<br>3۔ ہل رہا تھا / کم ہل رھا تھا / بالکل نہیں ہل رھا تھا	**S6.9**<br>نارمل / میڈیم / ابنارمل / ابنارمل<br>1. □ 2. □ 3. □ 9. □<br>1. □ 2. □ 3. □ 9. □<br>1. □ 2. □ 3. □ 9. □	**S6.24**<br>نارمل / میڈیم / ابنارمل / ابنارمل<br>1. □ 2. □ 3. □ 9. □<br>1. □ 2. □ 3. □ 9. □<br>1. □ 2. □ 3. □ 9. □
پہلے / آخری عمرکز صحت کا نام بیان کریں	1۔ اسپتال (گورنمنٹ)<br>2۔ اسپتال (این جی او)<br>3۔ اسپتال (گورنمنٹ)<br>4۔ ہیلتھ سینٹر (گورنمنٹ)<br>5۔ ہیلتھ سینٹر (این جی او)<br>6۔ ہیلتھ پوسٹ (گورنمنٹ)<br>7۔ ہیلتھ پوسٹ (این جی او)<br>8۔ پرائیویٹ ڈاکٹر / کلینک<br>9۔ پرائیویٹ ڈاکٹر / کلینک<br>10۔ تربیت یافتہ کمیونٹی ہیلتھ ورکر، نرس، مڈوائف<br>11۔ معلوم نہیں	S6.10<br>☐☐<br>____________<br>(صحت کے مرکز کا نام)	S6.25<br>☐☐<br>____________<br>(صحت کے مرکز کا نام)
اس بات کا فیصلہ (کہ صحت حاصل کرنے کیلئے کوئی کوشش کی جائے یا ریفرل لیا جائے)، کرنے اور کوئی کوشش کرنے کے درمیان کتنا وقت لگا؟		S6.11<br>__ __ دن<br>(DK = 99)<br>__ __ گھنٹے<br>(DK = 99)<br>__ __ منٹ<br>(DK = 99)	S6.26<br>__ __ دن<br>(DK = 99)<br>__ __ گھنٹے<br>(DK = 99)<br>__ __ منٹ<br>(DK = 99)
کیا پہلے یا آخری مرکز صحت جانے میں پیسوں کا خرچہ ہوا؟	1۔ جی ہاں<br>2۔ نہیں<br>99۔ معلوم نہیں	S6.12<br>☐ ***2 or 9 → S6.13***	S6.27<br>☐ ***2 or 9 → S6.28***
ان پیسوں کا بندوبست کہاں سے کیا؟<br>(ایک سے زیادہ جواب ہو سکتے ہیں)	1۔ پیسے پہلے سے موجود تھے<br>2۔ کسی سے منگے تھے<br>3۔ اپنی چیزیں بیچنی پڑیں<br>4۔ رشتہ داروں سے مدد لی<br>5۔ محلے کے فنڈ سے حاصل کیئے<br>6۔ حلومت کی اسکیم سے حاصل کیئے<br>7۔ دیگر (وضاحت کریں)<br>8۔ معلوم نہیں	S6.12.1<br>1. ☐<br>2. ☐<br>3. ☐<br>4. ☐<br>5. ☐<br>6. ☐<br>7. ☐<br>9. ☐	S6.27.1<br>1. ☐<br>2. ☐<br>3. ☐<br>4. ☐<br>5. ☐<br>6. ☐<br>7. ☐<br>9. ☐
ٹرانسپورٹ کا کونسا ذریعہ اختیار کیا؟<br>(ایک سے زیادہ جواب ہو سکتے ہیں)	1۔ پیدل چل کر<br>2۔ رکشہ / کشتی / کارٹ<br>3۔ بس<br>4۔ ٹیکسی / آٹو / ٹریکٹر<br>5۔ ایمبولینس<br>6۔ دیگر<br>7۔ کسی ٹرانسپورٹ کا بندوبست نہیں ہو سکا<br>9۔ معلوم نہیں	S6.13<br>1. ☐ ***If only walk***<br>2. ☐ ***→ S6.14.1***<br>3. ☐<br>4. ☐<br>5. ☐<br>6. ☐<br>7. ☐ ***→ S6.14.1***<br>9. ☐	S6.28<br>1. ☐ ***If only walk***<br>2. ☐ ***→ S6.29.1***<br>3. ☐<br>4. ☐<br>5. ☐<br>6. ☐<br>7. ☐ ***→ S6.29.1***<br>9. ☐
ٹرانسپورٹ کا کتنا خرچہ آیا؟		S6.14<br>__ __ __ __ unit<br>(DK=9999)	S6.29<br>__ __ __ __ unit<br>(DK = 9999)

بچوں کی اموات پر تحقیق کا سوالنامہ

فارم نمبر | کچی آبادی نمبر ☐☐☐☐ - گھر نمبر ☐☐☐ - بچہ نمبر ☐☐

کیا بچہ وفات سے پہلے مرکز صحت پہنچ گیا؟	1۔جی ہاں، بچہ وفات سے پہلے مرکز صحت پہنچ گیا<br>2۔ نہیں، گھر سے نکلنے سے پہلے وفات پا گیا<br>3۔ نہیں، رستے میں وفات پا گیا<br>4۔ نہیں، بچہ گھر سے نہیں نکلا / واپس کر دیا گیا / مرکز صحت نہیں پہنچ سکا۔<br>9۔ معلوم نہیں	S6.14.1<br>☐ **2, 3 → S6.39**<br>**4, 9 → Inst_4**	S6.29.1<br>☐ **2-9 → S6.39**
پہلے یا آخری مرکز صحت تک پہنچنے میں کتنا وقت لگا؟		S6.15<br>گھنٹے ___ ___<br>(DK = 99)<br>منٹ ___ ___<br>(DK = 99)	S6.30<br>گھنٹے ___ ___<br>(DK = 99)<br>منٹ ___ ___<br>(DK = 99)
پہلے یا آخری مرکز صحت نے بچے کے ساتھ کیا کیا؟<br>(ایک سے زیادہ جواب ہو سکتے ہیں)	1۔ آکسیجن دی<br>2۔ماسک اور بیگ کے ذریعے آکسیجن دی<br>3۔منہ کے ذریعے پانی پلایا<br>4۔منہ کے ذریعے اینٹی بایوٹک دی<br>5۔اینٹی ملیریل منہ کے ذریعے دی<br>6۔او آر ایس دیا<br>7۔وٹامن اے دیا<br>8۔دیگر ادویات منہ کے ذریعہ دیں<br>9۔گوشت میں انجکشن کے ذریعے ادویات دیں<br>10۔خون میں انجکشن کے ذریعے ادویات یا ڈرپ دیں<br>11۔باہر سے ادویات خریدنے کو کہا<br>12۔ آپریشن کیا<br>13۔اسپتال میں داخل کیا<br>14۔دیگر (اضاحت کریں)<br>15۔کچھ نہیں کیا<br>16۔معلوم نہیں	S6.16<br>1. ☐<br>2. ☐<br>3. ☐<br>4. ☐<br>5. ☐<br>6. ☐<br>7. ☐<br>8. ☐<br>9. ☐ ________<br>10. ☐<br>11. ☐<br>12. ☐ ________<br>13. ☐ stayed days<br>14. ☐ ________<br>15. ☐ → **S6.18**<br>99. ☐ → **S6.18**	S6.31<br>1. ☐<br>2. ☐<br>3. ☐<br>4. ☐<br>5. ☐<br>6. ☐<br>7. ☐<br>8. ☐<br>9. ☐ ________<br>10. ☐<br>11. ☐<br>12. ☐ ________<br>13. ☐ stayed days<br>14. ☐ ________<br>15. ☐ → **S6.33**<br>99. ☐ → **S6.33**
آپ نے ٹوٹل کتنا خرچہ کیا (دوائی ، داخلہ، ڈاکٹر کی فیس، ٹیسٹ، کمرے کا خرچہ وغیرہ کی مد میں)		S6.17<br>___ ___ ___ ___ ___ unit<br>(DK = 99999)	S6.32<br>___ ___ ___ ___ ___ unit<br>(DK = 99999)
کیا پہلے یا آخری مرکز صحت نے بچہ کو ریفر کیا؟	1۔جی ہاں<br>2۔ نہیں<br>99۔ معلوم نہیں	S6.18<br>☐ **2 or 9 → S6.19**	S6.33<br>☐ **2 or 9 → S6.34**
بچہ کو ریفر کیوں کیا گیا؟<br>(ایک سے زیادہ جواب ہو سکتے ہیں)	1. فراہم کنندہ مسئلہ حل کرنے کی صلاحیت رکھتا نہیں تھا.................<br>2. ضروری سامانات (جیسے کہ دوائیں، آئی وائی، اوکسیجن) دستیاب نہیں تھیں ....<br>3. ضروری سازات (جیسے کہ ایکسرے مشین) دستیاب نہیں تھیں ....<br>9. پتہ نہیں............................	S6.18.1<br>1. ☐<br>2. ☐<br>3. ☐<br>9. ☐	S6.33.1<br>1. ☐<br>2. ☐<br>3. ☐<br>9. ☐
کیا بچہ پہلے / آخری مرکز صحت سے زندہ نکلا؟	1۔جی ہاں<br>2۔ نہیں<br>99۔ معلوم نہیں	S6.19<br>☐ **2 → V5.4**	S6.34<br>☐ **2 → V5.4**
کیا پہلے / آخری مرکز صحت نے آپکو کوئی ھدایات دیں؟	1۔جی ہاں<br>2۔ نہیں<br>99۔ معلوم نہیں	S6.20<br>☐ **2 or 9 → Inst_3**	S6.35<br>☐ **2 or 9 → S6.37**

بچوں کی اموات پر تحقیق کا سوالنامہ

فارم نمبر ☐☐☐☐-☐☐☐-☐☐

کچی آبادی نمبر   گھر نمبر   بچہ نمبر

What did the <FIRST/LAST HEALTH PROVIDER> suggest that <u>you</u> do?<br><br>*Prompt:* Was there anything else?<br><br>(ایک سے زیادہ جواب ہو سکتے ہیں)	1. ماں کو دودھ پلائیں.................<br>2. زیادہ پانی پلائیں...........................<br>3. پیشہ ورانہ دودھ پلائیں ....<br>4. ORS دیں ....<br>5. منہ سے اینٹی بائیوٹک دیں ....<br>6. منہ سے اینٹی ملیریل دیں...........<br>7. منہ سے وٹامن A دیں ....<br>8. واپس آئیں فالو- آپ کے لیے..............<br>9. واپس آئیں یا بگڑتے ہوئے ریفر کیا جائے...........<br>10. موجودہ ریفرل مکمل کریں................<br>11. دیگر (وضاحت کریں)..............................<br>99. پتہ نہیں	S6.20.1<br>1. ☐<br>2. ☐<br>3. ☐<br>4. ☐<br>5. ☐<br>6. ☐<br>7. ☐<br>8. ☐<br>9. ☐<br>10. ☐<br>11. ☐ ________<br>99. ☐ → ***Inst_3***	S6.35.1<br>1. ☐<br>2. ☐<br>3. ☐<br>4. ☐<br>5. ☐<br>6. ☐<br>7. ☐<br>8. ☐<br>9. ☐<br>10. ☐<br>11. ☐ ________<br>99. ☐ → ***S6.37***
کیا آپ ان ہدایات پر عمل کر سکے؟	1۔ جی ہاں<br>2۔ نہیں<br>99۔ معلوم نہیں	S6.21<br>☐ ***9 → Inst_3***	S6.36<br>☐ ***9 → S6.37***
If <u>not</u> able to follow <u>all</u> the advice, ask: Did you have any concerns or problems that kept you from following the advice?<br><br>*If <u>able</u> to follow <u>all</u> the advice, ask:* Did you have to overcome any concerns or problems to follow the advice?	1۔ جی ہاں<br>2۔ نہیں<br>99۔ معلوم نہیں	S6.21.1<br>☐ ***2 or 9 → Inst_3***	S6.36.1<br>☐ ***2 or 9 → S6.37***
کوئی مسئلہ یا فکریں تھیں؟<br>پروب: کچھ اور تھا؟<br><br>(ایک سے زیادہ جواب ہو سکتے ہیں)	1. ہدایتوں کو سمجھ نہیں آئی<br>2. روزانہ کی معمولی ذمہ داریوں سے زیادہ وقت لگے۔<br>3. کوئی اور نے فیصلہ کیا (وضاحت دیں)۔<br>4. لازمیت سے زیادہ لاگت<br>5. مسئلہ روایتی دیکھ بھال کی ضرورت ہے۔<br>6. تجویز کی گئی دیکھ بھال کی ضرورت نہیں محسوس ہوئی۔<br>7. خدمت کا بچہ نقصان پہنچا سکتا ہے۔<br>8. خیال کیا کہ بچہ دیکھ بھال کے باوجود بچہ مر جائے گا۔<br>9. اگلے پروائیڈر کے پاس جانے کے لئے وقت نہیں تھا۔<br>10. بچہ بہت جلد مر گیا۔<br>11. دیگر (وضاحت دیں)۔<br>99. معلوم نہیں۔	S6.21.2<br>1. ☐<br>2. ☐<br>3. ☐ ________<br>4. ☐<br>5. ☐<br>6. ☐<br>7. ☐<br>8. ☐<br>9. ☐<br>10. ☐<br>11. ☐ ________<br>99. ☐	S6.36.2<br>1. ☐<br>2. ☐<br>3. ☐ ________<br>4. ☐<br>5. ☐<br>6. ☐<br>7. ☐<br>8. ☐<br>9. ☐<br>10. ☐<br>11. ☐ ________<br>99. ☐
ہدایت_3: S6.5 اگر کسی دوسرے صحت مہذب کے پاس لے جایا گیا تو → کو چیک کریں → S6.23			
اگر کسی دوسرے صحت مہذب کے پاس نہیں لے جایا گیا ہے، تو سوال کریں: (<پہلا صحت مہذب>/<آخری صحت مہذب>) <کے بعد، کیا<نام>...<br>[ہر حالت کے لئے چونکہائے۔]	1۔ کھانا نارمل کھا رہا تھا/ کم کھا رہا تھا/ بالکل نہیں۔ کھا رہا تھا<br>2۔ ہشاش بشاش تھا/ ہلکا بے ہوش تھا/ بے ہوش تھا<br>3۔ ہل رہا تھا/ کم ہل رہا تھا/ بالکل نہیں ہل رہا تھا	S6.22<br>نارمل میڈیم ابنارمل ابنارمل<br>1. ☐ 2. ☐ 3. ☐ 9. ☐<br>1. ☐ 2. ☐ 3. ☐ 9. ☐<br>1. ☐ 2. ☐ 3. ☐ 9. ☐	S6.37<br>نارمل میڈیم ابنارمل ابنارمل<br>1. ☐ 2. ☐ 3. ☐ 9. ☐<br>1. ☐ 2. ☐ 3. ☐ 9. ☐<br>1. ☐ 2. ☐ 3. ☐ 9. ☐
اگر دوسرے صحت مہذب کے پاس نہیں لے جایا گیا تو پوچھیں: کیا آپ کوئی فکری یا مسئلے تھے جو آپ کو روک رہے تھے کہ آپ نے <نام> کو کسی دوسرے صحت مہذب کے پاس نہیں لے جایا؟<br>اگر دوسرے صحت مہذب کے پاس لے جایا گیا تو پوچھیں: کیا آپ کوئی فکری یا مسئلے کو حل کرنا پڑا تھا جو آپ نے <نام> کو کسی دوسرے صحت مہذب کے پاس لے جانے کے لئے؟	1۔ جی ہاں<br>2۔ نہیں<br>99۔ معلوم نہیں	S6.23<br>☐ ***2 or 9 → Inst_4***	S6.38<br>☐ ***2 or 9 → S6.39***

بچوں کی اموات پر تحقیق کا سوالنامہ

فارم نمبر ☐☐☐☐-☐☐☐-☐☐

کچی آبادی نمبر | گھر نمبر | بچہ نمبر

**کیا آپ کوئی اور پریشانی یا مسئلہ رکھتے ہیں؟**<br>پروسپٹ: کیا کچھ اور تھا؟<br>[متعدد جوابات ممکن ہیں۔]	1. سمجھا کہ اب مزید دیکھ بھال کی ضرورت نہیں ہے ....<br>2. کوئی دوسرے ساتھ جانے والا نہیں تھا ....<br>3. عادی فرائض سے زیادہ وقت لگ گیا تھا۔<br>4. کوئی دوسرا شخص (وضاحت دیں) فیصلہ کرتا تھا ....<br>5. سفر کے لیے بہت دور تھا ....<br>6. کوئی سواری دستیاب نہیں تھی ....<br>7. خرچ (سفر، صحت کی دیکھ بھال، دیگر)<br>8. موجودہ دیکھ بھال سے خوش نہیں تھے .....<br>9. مسئلہ روایتی دیکھ بھال کی ضرورت تھی ...<br>10. سمجھا کہ بچہ سفر کرنے کے لئے بہت بیمار تھا ....<br>11. سمجھا کہ بچہ دیکھ بھال کے باوجود مر جائے گا 12. شام کا وقت تھا<br>..........................<br>13. بچہ جان بحق ہو گیا تھا جانے سے پہلے ....<br>14. دوسرے (وضاحت کریں) ....<br>99. پتہ نہیں	S6.23.1<br>1. ☐<br>2. ☐<br>3. ☐<br>4. ☐ ____<br>5. ☐<br>6. ☐<br>7. ☐<br>8. ☐<br>9. ☐<br>10. ☐<br>11. ☐<br>12. ☐<br>13. ☐ → **S6.39**<br>14. ☐ ____<br>99. ☐	S6.38.1<br>1. ☐<br>2. ☐<br>3. ☐<br>4. ☐ ____<br>5. ☐<br>6. ☐<br>7. ☐<br>8. ☐<br>9. ☐<br>10. ☐<br>11. ☐<br>12. ☐<br>13. ☐<br>14. ☐ ____<br>99. ☐
		...go to S6.24 (LAST PROVIDER)	

S6.39	<NAME> کا پہلا واقعہ ہوا؟ (پہلا / آخری ہیلتھ پرووائیڈر چھوڑنا / S6.5 بیماری کو پہچاننا / آخری کارروائی) کی موت کتنے دن بعد ہوئی، جب وہ<br>[اگر S6.4 = 2 بیماری کو پہچاننا تو: "... بیماری کو پہچاننا (کوئی دیکھ بھال نہیں کی گئی) ..."]	__ __ دن<br>*(<1 = 00; DK = 99)*

صارفہ 5: صحتی ریکارڈ (نیو سیل) اور 0 سے 59 مہینے کے بچوں کی موتوں اور مردہ پیدائش کے لئے

V5.4	کیا آپ کے پاس کوئی علاج کی رپوٹ		☐ ***2 or 9 → V5.10***
V5.5	کیا میں آپکے علاج کی رپوٹ دیکھ سکتا ہوں	1۔ جی ہاں<br>2۔ نہیں	☐ ***2 → V5.10***
V5.6	قریبی ہوئے آخری 2 وزٹس کی تاریخ درج کریں۔		__ __/__ __/__ __ __ __<br>D D M M Y Y Y Y<br>*(DK = 99/99/9999)*<br><br>__ __/__ __/__ __ __ __<br>D D M M Y Y Y Y<br>*(DK = 99/99/9999)*
V5.7	ان تاریخوں پر دو وزن درج کریں۔		__ __ __ __ گرام<br>*(DK = 9999)*<br><br>__ __ __ __ گرام<br>*(DK = 9999)*
V5.8	تحریر کردہ آخری نوٹ کس تاریک کو لکھا گیا؟		__ __/__ __/__ __ __ __<br>D D M M Y Y Y Y<br>*(DK = 99/99/9999)*

بچوں کی اموات پر تحقیق کا سوالنامہ

فارم نمبر: ☐☐☐☐-☐☐☐-☐☐

کچی آبادی نمبر — گھر نمبر — بچہ نمبر

V5.9	دیگر معلومات یہاں پر درج کریں:		
	______________________________<br>______________________________<br>______________________________<br>______________________________<br>______________________________<br>______________________________<br>______________________________		
V5.10	کیا "ڈیتھ سرٹیفکیٹ" جاری ہوا؟ **(صرف ایک جواب)**	☐1. جی ھاں<br>☐2. جی نہیں<br>☐99. معلوم نہیں	☐ ***2 or 9 → S1.1***
V5.11	کیا میں "ڈیتھ سرٹیفکیٹ" دیکھ سکتا ہوں؟		☐ ***2 → S1.1***
V5.12	وفات کی بڑی (حتمی) وجہ کاپی کریں		
V5.13	وفات کی لکھی گئی وجوہات میں سے پہلی وجہ کاپی کریں		
V5.14	وفات کی لکھی گئی وجوہات میں سے پہلی وجہ کاپی کریں		
V5.15	وفات کی لکھی گئی وجوہات میں سے تیسری وجہ کاپی		
V5.16	کریں<br>وفات کی وجہ بننے والے عناصر (مثلا، سگریٹ) کاپی کریں		

SA Module 1: اب میں مرحوم بچہ کی ماں کے بارے میں سوال کرنا چاہتا ھوں

S1.2	اس وقت (یا مرتے وقت، اگر مرحوم)، بچہ کی ماں کی کتنی عمر ہے؟		__ __ سال<br>*(DK = 99)*
S1.3	ماں نے اسکول میں کتنے سال تعلیم مکمل کی؟		__ __ سال<br>*(<1 = 00; DK = 99)*
S1.4	کیا ماں:	1۔ سہاگن (خاوند زندہ) ہے؟<br>2۔ بیوہ ہے؟<br>3۔ طلاق یافتہ ہے؟<br>99۔ معلوم نہیں	☐
S1.4.1	شادی کے وقت ماں کی عمر کتنی تھی؟		__ __ سال<br>*(DK = 99)*

فارم نمبر ☐☐☐☐-☐☐☐-☐☐

کچی آبادی نمبر　گھر نمبر　بچہ نمبر

بچوں کی اموات پر تحقیق کا سوالنامہ

S1.4.2	اب تک بچے کے والد نے کتنے سال کی تعلیم مکمل کی ہے؟		سال ___ ___ (<1 = 00; DK = 99)
اب میں کچھ مزید سوالات آپ کے گھر کے بارے میں پوچھنا چاہتا ہوں-			
S1.5	بچے کی بیماری کے دوران (یا ماں کے حمل کے آخری وقت میں) اس گھر کا خرچہ کون سنبھالتا تھا؟ (صرف ایک جواب) -	1۔ بچہ کا والد<br>2۔ بچہ کی ماں<br>3۔ دیگر (وضاحت کریں)<br>99۔ معلوم نہیں	☐ 99 → S1.7
S1.6	خرچہ سنبھالنے والا / والی، اس وقت کیا کام کرتا / کرتی تھی؟	1۔ کسان<br>2۔ دکاندار<br>3۔ گھر کا نوکر / نوکرانی<br>4۔ فیکٹری میں کام<br>5۔ گاڑی کا مکینک<br>6۔ ریڑھی والا<br>7۔ جاب کرتا ہے<br>8۔ ملک سے باہر<br>9۔ دیگر<br>99۔ معلوم نہیں	☐☐<br>________________
S1.7	کیا یہ وہی گھر ہے جہاں آپ لوگ بچے کی بیماری کے دوران (یا ماں کے حمل کے آخری زمانے میں) رہا کرتے تھے؟	☐1۔ جی ہاں<br>☐2۔ جی نہیں<br>☐99۔ معلوم نہیں	☐ 1 → S1.10<br>9 → V5.17
S1.8	اس وقت آپ لوگ کہاں ٹھہرے ہوئے تھے؟	1۔ بچہ کی ماں کے گھر میں<br>2۔ اپنے گھر میں<br>3۔ کسی اور گھر میں<br>99۔ معلوم نہیں	☐<br>99 → V5.17
S1.9	اس گھر کا کیا پتہ ہے؟	________________<br>________________<br>________________<br>________________	☐☐<br>☐☐<br>☐☐☐<br>☐☐☐☐

بچوں کی اموات پر تحقیق کا سوالنامہ

فارم نمبر ☐☐☐☐-☐☐☐-☐☐

کچی آبادی نمبر — گھر نمبر — بچہ نمبر

S1.10	بچے کی بیماری کے وقت آپ لوگ اس جگہ پر کتنے عرسے سے رہتے تھے؟		___ ___ سال<br>*(<1 = 00; DK =99)*
S1.11	اس گھر سے معالج یا مرکز جانے میں عام طور پر کتنا وقت لگتا ہے؟		___ ___ گھنٹے<br>*(DK = 99)*<br>___ ___ منٹ<br>*(DK = 99)*
ہدایت_3 ← ایس 2.1.1 (اختیاری ماڈیول 2 شامل ہے) یا وی 5.17			
اب ہم آپکے علاقے کے بارے میں کچھ پوچھنا چاہتے ہیں SA Module 2:			
S2.1.1	پچھلے تین سالوں میں آپکے علاقے کے لوگوں نے آپس میں مل کر ان میں سے کتنے مساءل پر ایسا کامم کیا کہ علاقہ مکینوں کو فاءدہ ہوا ہو؟	1۔ تعلیمی نظام<br>2۔ صحت کے مسائل<br>3۔ علاقہ مکینوں کے روز گار کیلی ئے<br>4۔ بیت المال سے متعلق کام یا ادھار لین دین<br>5۔ روڈوں کا نظام<br>6۔ گاڑیوں یا سواریوں کی آمد ہر فت پر<br>7۔ پانی کی فراہمی کیلیے (گھروں کے پانی کی فراہمی)<br>8۔ نکاسیء آب<br>9۔ علاقے میں عدل و انصاف قائم کرنے کی کوشش<br>10۔ علاقے کی سیکیورٹی کیلیے<br>11۔ دیگر	Yes No DK<br>1. ☐ 2. ☐ 9. ☐<br>1. ☐ 2. ☐ 9. ☐<br>1. ☐ 2. ☐ 9. ☐<br>1. ☐ 2. ☐ 9. ☐<br>1. ☐ 2. ☐ 9. ☐<br>1. ☐ 2. ☐ 9. ☐<br>1. ☐ 2. ☐ 9. ☐<br>1. ☐ 2. ☐ 9. ☐<br>1. ☐ 2. ☐ 9. ☐<br>1. ☐ 2. ☐ 9. ☐<br>1. ☐ 2. ☐ 9. ☐<br>1. ☐ 2. ☐ 9. ☐<br>______________________
			☐
S2.2	اپنے بچے کی بیماری کے دوران کیا آپنے اپنے علاقے کے کسی بھی فرد، تنظیم یا گروپ سے مدد مانگی تھی؟	☐1. جی ھاں<br>☐2. جی نھیں<br>☐99. معلوم نھیں	☐ **2 or 9 → S2.3.1**

بچوں کی اموات پر تحقیق کا سوالنامہ

فارم نمبر ☐☐☐☐-☐☐☐-☐☐

کچی آبادی نمبر　گھر نمبر　بچہ نمبر

S2.2.1	کیا آپ نے ان لوگوں میں سے کسی سے مدد مانگی؟	1۔خاندان<br>2۔پڑوسی<br>3۔دوست/احباب<br>4۔دینی/مذہبی افراد<br>5۔علاقے کے سربراہ<br>6۔علاقے کی کسی تنظیم یا گروپ سے<br>7۔دیگر (وضاحت کریں)	Yes No DK<br>1. ☐ 2. ☐ 9. ☐<br>1. ☐ 2. ☐ 9. ☐<br>1. ☐ 2. ☐ 9. ☐<br>1. ☐ 2. ☐ 9. ☐<br>1. ☐ 2. ☐ 9. ☐<br>1. ☐ 2. ☐ 9. ☐<br>1. ☐ 2. ☐ 9. ☐<br>____________
S2.2.2	کیا یہ وہی افراد ہیں جو مدد مانگنے پر اکثر تعاون کرتے کرتے ہیں اور لوگوں کی مدد کرتے ہیں؟(صرف ایک جواب)	☐**1. جی ہاں**<br>☐**2. جی نہیں**<br>☐**99. معلوم نہیں**	
S2.3.1	علاقہ مکینوں (یا علاقے کی تنظیموں) کی طرف سے کبھی آپ کو ان باتوں کیلیئے انکار کیا گیا ہو؟	1. **تعلیمی بات پر**<br>**2. صحت کے کسی مسئلہ پر**<br>**3. روزگار کے کسی مسئلہ پر**<br>**4. ادھارکے لین دین پر**<br>**5. سواری دینے پر**<br>**6. پانی کی فرا ہمی پر**<br>**7. نکاسیء آب پر**<br>**8. عدل و انصاف کی فراہمی پر**<br>**9. سیکیورٹی مہیا کرنے پر**<br>**10. دیگر**	Yes No DK<br>1. ☐ 2. ☐ 9. ☐<br>1. ☐ 2. ☐ 9. ☐<br>1. ☐ 2. ☐ 9. ☐<br>1. ☐ 2. ☐ 9. ☐<br>1. ☐ 2. ☐ 9. ☐<br>1. ☐ 2. ☐ 9. ☐<br>1. ☐ 2. ☐ 9. ☐<br>1. ☐ 2. ☐ 9. ☐<br>1. ☐ 2. ☐ 9. ☐<br>1. ☐ 2. ☐ 9. ☐<br>1. ☐ 2. ☐ 9. ☐<br>____________

بچوں کی اموات پر تحقیق کا سوالنامہ

فارم نمبر | کچی آبادی نمبر | گھر نمبر | بچہ نمبر

جوابدہندہ کا شکریہ ادا کریں۔
جوابدہندہ سے آخر میں پوچھئے کہ اگر وہ کچھ کہنا چاہتا/چاہتی ہے تو بتائیے۔ آپ جوابدہندہ کو روکے بغیر، اس کی کہی گئی تمام مزید معلومات نیچے لکھیے۔

انٹرویو کا اختتام

اس انٹرویو میں جمع کی گئی معلومات سے متعلق اپنی رائے نیچے لکھئے۔
