# Supplementary material for: Validation of CHERG’S Verbal Autopsy-Social Autopsy (VASA) tool for ascertaining determinants and causes of under-five child mortality in Pakistan
Source: PLoS One. 2023 Dec 18;18(12):e0278149. doi: 10.1371/journal.pone.0278149 (PMC10727362; doi:10.1371/journal.pone.0278149)
Supplement: Attachment — Submitted filename: second_Review file.pdf [file pone.0278149.s005.pdf]

PLOS ONE

# Validation of CHERG’S Verbal Autopsy-Social Autopsy (VASA) tool for ascertaining determinants and causes of under-five child mortalities in Pakistan.

--Manuscript Draft--

| | |
|---|---|
| **Manuscript Number:** | PONE-D-21-23446R2 |
| **Article Type:** | Research Article |
| **Full Title:** | Validation of CHERG’S Verbal Autopsy-Social Autopsy (VASA) tool for ascertaining determinants and causes of under-five child mortalities in Pakistan. |
| **Short Title:** | Validation of CHERG’S Verbal/Social Autopsy (VASA) tool for under-five child mortalities in Pakistan. |
| **Corresponding Author:** | Muhammad Bilal Siddiqui, M.D., MPH<br>University of Malaya Faculty of Medicine<br>Kuala Lumpur, PAKISTAN |
| **Keywords:** | children; under-five; mortality; Verbal autopsy; Social Autopsy; validation; VASA |
| **Abstract:** | The major chunk (40%) global burden of under-five mortality is concentrated in places like Pakistan (8%), India (16%), Nigeria (16.5%), and the Democratic Republic of the Congo (6%), where an unknown proportion of deaths go unreported. In low-resource settings and globally, the Verbal Autopsy-Social Autopsy (VASA) tool (involve data from a well-structured questionnaire about the signs and symptoms and social circumstances of the deceased) can help estimate under-five mortalities; assign medical and social causes of deaths and determine the distribution of relevant determinants. Uncertainty about missing data in high burden countries like Pakistan requires a validated and reliable VASA tool. This is the first study to validate CHERG’s verbal autopsy/social autopsy tool globally. In Pakistan, data from such a valid and reliable tool is vital. This paper reports on the VASA tool's validation in Karachi, Pakistan. Validity and reliability of the CHERG VASA-tool were tested using face, content, discriminant validation, and reliability tests on one hundred randomly selected mothers who had recently experienced a child death event. Data were computed on SPSS (version-21) and R software. Testing revealed high I-CVI (>81.43%); high Cronbach's Alpha (0.843) ; the accuracy of between 75-100% of the discriminants classifying births to live and stillbirths. The tool showed Item-content Validity Index (ICVI) (>82.07% and 88.98% respectively) with high accuracy (92% and 97% respectively) for assigning biological and social causes of child deaths, respectively. The CHERG VASA questionnaire is relevant to the conceptual framework and valid in Pakistan. This valid tool can assign accurate medical and non-medical causes of child mortality cases occurring in Pakistan. |
| **Order of Authors:** | Muhammad Bilal Siddiqui, M.D., MPH |
| | Chiu Wan Ng |
| | Wah Yun Low |
| | Khadijah Abid |
| **Response to Reviewers:** | AUTHORS COMMENTSAUTHOR’S COMPLIANCE/COMMENTS<br>Reviewer #4: Line28,66,68: the term mortalities is not appropriate – needs to be corrected as mortality – including the title (Done, see title, please see whole document. I have amended your suggestion throughout the document)<br>Line 210: what was the educational background of the data collectors (Done. please see line 406-407)<br>Table1: the column for frequency can only depict n. denominator of 100 from each cell needs to be removed. The percent column can mention (%) in column heading & the % symbol from each cell can be removed. (Done, please see all tables)<br>All Tables: The percent column can mention (%) in column heading & the % symbol from each cell can be removed. (Done, please see all tables)<br>Table 1:please provide details about unimproved & improved facility mentioned under access to toilets (described in the table 1 at the bottom of the respective table. Also referenced).<br>Table 7: cause of death biological – sensitivity % is missing (Done, please see table |

Powered by Editorial Manager® and ProduXion Manager® from Aries Systems Corporation

| | |
|---|---|
| | no-08)<br>Methods section could include details about the characteristics of the deceased children. Age & gender, cause of death, hospitalization, institutional delivery, antenatal history, hospital records if available etc (Done in table 5)<br>Line 443:avoid duplication of word specifically (Done)<br>Line 467: it is preferable to avoid superlatives like highly important (Done)<br>Line 484-485: the term error is missing from the statement (Done, please see Line no 1351)<br>Line 490-492: the recommendation is not well suited, considering the present study objectives & hence needs to be removed. (Deleted)<br>Reviewer #5: Sample Size:<br>What was the data source for identification of mothers who had lost their Under five children at each of the 12 sites listed? Authors suggest that death registration is far from complete in Karachi city (deleted from the manuscript as the statement was confusing) yet they claim mothers were randomly included based on Proportional uota sampling. How was this done? Please clarify? (Done. clarified between lines 225-257)(deleted from the manuscript as the statement was confusing)<br>(Done. clarified between lines 225-257)<br>Line 131 135 : "These 12 locations throughout the city of Karachi were chosen based on criteria to identify geographical points throughout the entire city where residents belonging to the most common and frequently found ethnic backgrounds (Punjabi, Sindhi, Balochi, Siraiki, Pathan, Urdu speaking, Kashmiri, Hindko,and Brahvi) live within at least 2 kilometres of residential locations. This criterion, ensured that the sample is representative of the general population." Unclear? What do the authors mean by living within 2 kms of residential locations? Please rephrase. (typo error. Rewritten. Done, please see Line 225-252)<br>Line 170: Authors describe CHERG VASA tool as a uantitative tool. This is not so. It includes open ended text fields as well. (Done, please see Line 355-357)<br>Line 216-218: Authors have not elaborated details of PCVA process. Did a single physician review a particular VA and assign most probable CoD? If so it introduces a subjective bias? Did more than one physician independently review each VA form? If so what was the agreement between physicians and how were disagreements adjudicated? How were the responses made available to the physicians? It is well known that in PCVA CoD is largely assigned based on the text narrative rather than the close ended responses. What measures were taken to minimize this phenomenon? (explained between lines 412-419).<br>In the PAPI what was the completion rate for the entire questionnaire after accounting for skip patterns? (Done, please see Line no 401-403)<br>Section V5.12 - V5.16 of the CHERG VASA questionnaire record CoD listed on the death certificate. Was this information masked from the Physicians? If not it calls into question the very high values of 97% accuracy for CoD assigned based on VA and may invalidate the findings of the entire study. Please give details (Done, Line no: 410-431) |
| Additional Information: | |
| Question | Response |
| **Financial Disclosure**<br><br>Enter a financial disclosure statement that describes the sources of funding for the work included in this submission. Review the submission guidelines for detailed requirements. View published research articles from *PLOS ONE* for specific examples.<br><br>This statement is required for submission and **will appear in the published article** if the submission is accepted. Please make sure it is accurate. | The author(s) received no specific funding for this work. |

| | |
|---|---|
| **Unfunded studies**<br>Enter: *The author(s) received no specific funding for this work.*<br><br>**Funded studies**<br>Enter a statement with the following details:<br>• Initials of the authors who received each award<br>• Grant numbers awarded to each author<br>• The full name of each funder<br>• URL of each funder website<br>• Did the sponsors or funders play any role in the study design, data collection and analysis, decision to publish, or preparation of the manuscript?<br>• **NO** - Include this sentence at the end of your statement: *The funders had no role in study design, data collection and analysis, decision to publish, or preparation of the manuscript.*<br>• **YES** - Specify the role(s) played.<br><br>* typeset | |
| **Competing Interests**<br><br>Use the instructions below to enter a competing interest statement for this submission. On behalf of all authors, disclose any competing interests that could be perceived to bias this work—acknowledging all financial support and any other relevant financial or non-financial competing interests.<br><br>This statement is required for submission and **will appear in the published article** if the submission is accepted. Please make sure it is accurate and that any funding sources listed in your Funding Information later in the submission form are also declared in your Financial Disclosure statement.<br><br>View published research articles from *PLOS ONE* for specific examples. | The authors have declared that no competing interests exist. |

*Powered by Editorial Manager® and ProduXion Manager® from Aries Systems Corporation*

| | |
|---|---|
| **NO authors have competing interests**<br><br>Enter: *The authors have declared that no competing interests exist.*<br><br>**Authors with competing interests**<br><br>Enter competing interest details beginning with this statement:<br><br>*I have read the journal's policy and the authors of this manuscript have the following competing interests: [insert competing interests here]*<br><br>* typeset | |
| **Ethics Statement**<br><br>Enter an ethics statement for this submission. This statement is required if the study involved:<br><br>• Human participants<br>• Human specimens or tissue<br>• Vertebrate animals or cephalopods<br>• Vertebrate embryos or tissues<br>• Field research<br><br>Write "N/A" if the submission does not require an ethics statement.<br><br>General guidance is provided below. Consult the submission guidelines for detailed instructions. **Make sure that all information entered here is included in the Methods section of the manuscript.** | The ethical approval of this study was gained from the Medical Ethics Committee, University Malaya Medical Center (201412-847), Kuala Lumpur; and Advance Educational Institute and Research Centre (MU/ECA/48/776), Karachi, Pakistan. |

Powered by Editorial Manager® and ProduXion Manager® from Aries Systems Corporation

| | |
|---|---|
| **Format for specific study types**<br><br>**Human Subject Research (involving human participants and/or tissue)**<br>• Give the name of the institutional review board or ethics committee that approved the study<br>• Include the approval number and/or a statement indicating approval of this research<br>• Indicate the form of consent obtained (written/oral) or the reason that consent was not obtained (e.g. the data were analyzed anonymously)<br><br>**Animal Research (involving vertebrate animals, embryos or tissues)**<br>• Provide the name of the Institutional Animal Care and Use Committee (IACUC) or other relevant ethics board that reviewed the study protocol, and indicate whether they approved this research or granted a formal waiver of ethical approval<br>• Include an approval number if one was obtained<br>• If the study involved *non-human primates*, add *additional details* about animal welfare and steps taken to ameliorate suffering<br>• If anesthesia, euthanasia, or any kind of animal sacrifice is part of the study, include briefly which substances and/or methods were applied<br><br>**Field Research**<br><br>Include the following details if this study involves the collection of plant, animal, or other materials from a natural setting:<br>• Field permit number<br>• Name of the institution or relevant body that granted permission | |
| **Data Availability**<br><br>Authors are required to make all data underlying the findings described fully available, without restriction, and from the time of publication. PLOS allows rare exceptions to address legal and ethical concerns. See the PLOS Data Policy and FAQ for detailed information. | Yes - all data are fully available without restriction |

| | |
|---|---|
| A Data Availability Statement describing where the data can be found is required at submission. Your answers to this question constitute the Data Availability Statement and **will be published in the article**, if accepted.<br><br>**Important:** Stating 'data available on request from the author' is not sufficient. If your data are only available upon request, select 'No' for the first question and explain your exceptional situation in the text box.<br><br>Do the authors confirm that all data underlying the findings described in their manuscript are fully available without restriction? | |
| **Describe where the data may be found in full sentences. If you are copying our sample text, replace any instances of XXX with the appropriate details.**<br><br>• If the data are **held or will be held in a public repository**, include URLs, accession numbers or DOIs. If this information will only be available after acceptance, indicate this by ticking the box below. For example: *All XXX files are available from the XXX database (accession number(s) XXX, XXX.*).<br>• If the data are all contained **within the manuscript and/or Supporting Information files**, enter the following: *All relevant data are within the manuscript and its Supporting Information files.*<br>• If neither of these applies but you are able to provide **details of access elsewhere**, with or without limitations, please do so. For example:<br><br>*Data cannot be shared publicly because of [XXX]. Data are available from the XXX Institutional Data Access / Ethics Committee (contact via XXX) for researchers who meet the criteria for access to confidential data.*<br><br>*The data underlying the results presented in the study are available from (include the name of the third party* | The datasets used and analysed during the current study are available on the following link. https://figshare.com/s/883fe45ea7da71483acd. |

| | |
|---|---|
| *and contact information or URL).*<br>• This text is appropriate if the data are owned by a third party and authors do not have permission to share the data.<br><br>* typeset | |
| Additional data availability information: | |

Powered by Editorial Manager® and ProduXion Manager® from Aries Systems Corporation

Manuscript Click here to access/download;Manuscript;MANUSCRIPT 7 SEPT 2022.doc

# Validation of CHERG'S Verbal Autopsy-Social Autopsy (VASA) tool for ascertaining determinants and causes of under-five child mortality in Pakistan.

**Muhammad Bilal Siddiqui[1,3*], Chiu Wan Ng[1], Wah Yun Low[2], Khadijah Abid[3]**

[1]Department of Social and Preventive Medicine, Faculty of Medicine, University of Malaya, Kuala Lumpur, Malaysia.
[2]Dean's Office, Faculty of Medicine, University of Malaya, Kuala Lumpur, Malaysia.
[3]Department of Public Health, Faculty of Life Sciences. Research Villa, Shaheed Zulfiqar Ali Bhutto Institute of Science and Technology (SZABIST), Clifton, Karachi, Pakistan.

***Corresponding Author:**
**Muhammad Bilal Siddiqui**
Email: muhammad.bilal@szabist.edu.pk (MBS)
drbilals@siswa.um.edu.my

## Abstract

The majority (40%) of the world's under-five mortality burden is concentrated in nations like Nigeria (16.5%), India (16%), Pakistan (8%), and the Democratic Republic of the Congo (6%), where an undetermined number of under-five deaths go unrecorded. In low-resource settings throughout the world, the Verbal Autopsy-Social Autopsy (VASA) technique may assist assess under-five mortality estimates, assigning medical and social causes of death, and identifying relevant determinants. Uncertainty regarding missing data in high-burden nations like Pakistan necessitates a valid and reliable VASA instrument. This is the first study to validate CHERG's VASA tool globally. In Pakistan, data from such a valid and reliable tool is vital for policy. This paper reports on the VASA tool in Karachi, Pakistan. Validity and reliability of the CHERG

VASA tool were tested using face, content, discriminant validation, and reliability tests on one hundred randomly selected mothers who had recently experienced an under-five child death event. Data were computed on SPSS (version-21) and R software. Testing revealed high I-CVI (>81.43%); high Cronbach's Alpha (0.843); the accuracy of between 75-100% of the discriminants classifying births to live and stillbirths; and I-CVI (>82.07% and 88.98% respectively) with high accuracy (92% and 97% respectively) for assigning biological and social causes of child deaths, respectively. The CHERG VASA questionnaire was found relevant to the conceptual framework and valid in Pakistan. This valid tool can assign accurate medical and non-medical causes of child mortality cases occurring in Pakistan.

# Introduction

Verbal autopsy (VA) is a technique that involves structured questions focusing on the signs and symptoms the deceased case faced during the illness but before the mortality incident. VA is used to record various relevant biological determinants (BDs) linked with child mortality incidents, and based on such information, VA helps in assigning the medical cause of death (MCoD). Similarly, social autopsy (SA), a relatively new technique with a questionnaire structure similar to VA, helps record non-biological determinants (especially the health-seeking

process, and issues and barriers in the process of accessing health care during the child illness process but before the mortality event) so that the social cause of death (SCoD) of any child death can be concluded. VA has been used in low-resource settings when the cause of any child death (CoD) has not been ascribed by a health care provider(Moyer et al., 2017; Nichols et al., 2018; Siddiqui, Ng, & Low, 2016). In contrast to VA, SA is not typically used for child mortality in the majority of developing countries, especially Pakistan. Both VA and SA not only provide the MCoD and SCoD to the child mortalities respectively but also assist in updating epidemiological estimates on the mortality which have been missed to be recorded by the health system in these geographies(Gouda et al., 2017; Lisa-Marie Thomas, Lucia D’Ambruoso, 2018). Although VA has been used in nations with little resources, there is still a need to investigate a wider range of data on the societal factors that contribute to child mortality(Koffi, Mleme, et al., 2015; Siddiqui, Ng, & Low, 2016). Hence, in a more recent timeframe, a concept of running through the missed mortality case with a VA and SA tool has been attempted in some countries (Cameroon(Koffi, Libite, et al., 2015),(Koffi, Mleme, et al., 2015), Mozambique(Källander et al., 2019), Niger(H. D. Kalter et al., 2015),(H. Kalter et al., 2016),(Koffi et al., 2016), Uganda(Waiswa et al., 2010),(Källander et al., 2011),(Hildenwall et al., 2008),(Källander et al., 2008), Rwanda(Navale et al., 2017),(Kagabo et al., 2018), Malawi(Koffi, Mleme, et al., 2015), Nigeria(Koffi et al., 2017), Guinea(Schumacher R Diallo MO, Keita DR, Kalter HD, Pasha O:, 2002)) which helped to get detailed evidence of social causes of different medically assigned CoDs. However, these attempts made use of different VA and SA tools in combinations[4,6–9],(Siddiqui et al., 2018).

The Child Health Epidemiology Reference Group (CHERG) was established in 2001 with the support of WHO and UNICEF to provide technical guidance to relevant institutions and

organisations for collecting evidence on determinants and causes of child mortality and improving global child survival epidemiological estimates(Koffi, Libite, et al., 2015),(Siddiqui et al., 2017). The agency developed an integrated VASA tool based on a most holistic approach for recording different biological determinants (child-specific, maternal-specific, determinants across the CoC, and preventive measures taken before and during the pregnancy and child illness) and non-biological determinants (NBDs) identified through several models (The Three Delay Model), conceptual framework (TPtoSCF); and several recommendations for VA suggested by the World Health Organization (WHO & Department of Measurement and Health Information Systems evidence and information for policy, 2004) to record different biological and non-biological determinants affecting the under-five mortality(Siddiqui et al., 2018),(CHERG, 2018a),(Koffi et al., 2019),(H. D. Kalter et al., 2011). In addition to recording biological determinants, this tool also focuses on all the barriers which come across as hindrances throughout the health-seeking process when the child was ill(Siddiqui et al., 2017). This tool is one of the assets that can be utilized to acquire information on the relevant biological and non-biological determinants of child mortality for every missed child mortality case on a single visit to the household and has already supported several countries to gather evidence on the medical and social causes to child deaths(Bensaïd et al., 2016; H. Kalter et al., 2016; Siddiqui et al., 2018). The extensive data obtained from CHERG VASA tool based investigations provides highly elaborated evidence and information on extended determinants of child mortality which is of great public health importance(Bensaïd et al., 2016; Gouda et al., 2017; H. Kalter et al., 2016)

Similar to any data collection tool, it's always been a concern whether the data collected through VA and SA are correct, reliable, and represent the information that the researcher intends to collect. The process of validation involves testing the questionnaire on validity and reliability parameters. To obtain the best possible data, many efforts have been undertaken globally in refining the methodology of VA(Aggarwal et al., 2013; Marsha et al., 2003; Midhet, 2008; Mpimbaza et al., 2015; Murray et al., 2014; Nausheen et al., 2013; Soofi et al., 2015). In continuation of such efforts, a few validation studies have been undertaken globally as well in Pakistan(IGME, 2020) to assess the capacity of VA tools in providing precise and reliable evidence on the causes of child mortality(Aggarwal et al., 2013; Marsha et al., 2003; Nausheen et al., 2013; Soofi et al., 2015). However, no attempts have been made to validate CHERG's integrated VASA tool. Considering the current status of child mortality estimates in Pakistan(Afzal & Yusuf, 2013; IGME, 2020; Nisar & Dibley, 2014), CHERG's integrated VASA tool was validated to gather evidence on a comprehensive list of medical and social determinants and medical and social causes of under-five mortality in Pakistan, where child mortality cases may be missed to be registered under national CRVS and Health Management Information System. This is the first time a VASA integrated tool has been validated so thoroughly, concentrating on most of the approaches used to evaluate validity and reliability. We expect the validated tool would provide comprehensive data on under-five mortality in Pakistan. This paper presents the results of the validation of the CHERG VASA tool. The validated instrument will be used to conduct a future VASA child mortality survey in Pakistan's underprivileged community.

# Materials and Methods

This validation study is a component of the *Karachi VASA child mortality study*(Siddiqui, Ng, Low, et al., 2016) and is designed to objectively assess the validity and reliability estimates of the CHERG's VASA questionnaire, which was later utilized in *Karachi VASA child mortality study*.

## The sample size

Although there are no specific guidelines for the sample size of respondents required for pre-survey validation of the VASA tool(Anthoine et al., 2014), the only available guideline suggests(Hertzog, 2008) that, in general, the sample size required for any questionnaire validation should be a minimum of 20% of the total sample size of the study. Since 400 under-5 died cases were included in the Karachi VASA child mortality study, 20% of 400 cases (80 cases) were selected from the study sites to participate in the questionnaire validation study. The original sample size of 80 was increased to 100 to overcome the issue of potential data entry errors. Mothers of these 100 under-five deceased cases (n=100) were selected from 12 selected sites i.e., Cheneser goth (CG), Mujahid colony (MC), Haryana Colony (HC), Sultanabad (SD), Bilal colony (BC), Sherabad (SA), Yaseenabad (YS), Landhi slums (LS), Khuda ki Basti (KKB), Safora goth (SG), Malir slum (MS), and Sultanabad (SD), based on the number of deaths occurred in these sites during the past one year (from June 1, 2015, to May 3, 2016) before the data collection. These 12 study sites were located in five of the city's districts, into which the city was subdivided at the time of the study, namely, District Malir, District Central, District South, District West, District East, and District Korangi. Two study sites (one urban and another one urban-slum) were randomly selected from each of the six districts, making a total of 12 study sites for research purposes. The urban sites and the urban-slum sites were classified based on the

definition of a slum from UN-Habitat(UN-Habitat, 2003). To ensure that the sample was representative of the entire population, the sample (100 mothers of deceased under-five deaths, falling under strict inclusion and exclusion criteria) was drawn from all six districts of Karachi.

## Sampling methodology

The estimates of all the under-five deaths (occurred between 01st June 2015 till 03rd May 2016) from each of the study sites were identified from each site through multiple sources (household visits i.e., snowball sampling; local graveyards; local town health and union council offices). To fulfil the sample size of 100 respondents for the validation study, each identified study site was allocated a quota based on the **proportionate quota sampling technique** (proportionally of the overall sample size required for the study). The details (quota calculation) are available in Table 1.

**Table 1: SAMPLING QUOTA CALCULATION**

| S. No | STUDY SITE | SITE CODE | TOTAL IDENTIFIED DEATHS IN EACH SITE (A) | (DEATH IN EACH SITE/TOTAL DEATHS IN 12 SLUMS) X (SAMPLE SIZE) (B) | QUOTA TO BE ALLOCATED FOR EACH SITE FOR VALIDATION (C) |
|---|---|---|---|---|---|
| 1 | Cheneser goth | CG | 210 | 210/1926 | 11 |
| 2 | Mujahid colony | MC | 168 | 168/1926 | 9 |
| 3 | Hijrat colony | HC | 159 | 159/1926 | 8 |
| 4 | Haryana colony | HC | 57 | 57/1926 | 3 |
| 5 | Sultanabad | ST | 111 | 111/1926 | 6 |
| 6 | Bilal colony | BC | 189 | 189/1926 | 10 |
| 7 | Sherabad | SA | 243 | 243/1926 | 13 |
| 8 | Yaseenabad | YS | 183 | 183/1926 | 9 |
| 9 | Landhi slum | LS | 150 | 150/1926 | 8 |
| 10 | Khuda ki Basti | KKB | 126 | 126/1926 | 6 |
| 11 | Safora goth | SG | 183 | 183/1926 | 9 |
| 12 | Malir slum | MS | 147 | 147/1926 | 8 |
| | Total | | 1926 | | 100 |

## Inclusion and exclusion criteria

To reduce response bias, only women whose children under the age of five passed away during the previous year (from 01$^{st}$ June 2015 till 3$^{rd}$ May 2016) were counted in this validation study.

## Ethical clearance for Karachi VASA child mortality study

The Medical Ethics Committee, University Malaya Medical Center (UMMC), located in Kuala Lumpur, Malaysia; and Advance Educational Institute and Research Centre (AEIRC), located in Karachi Pakistan granted the ethical clearance (with ethical clearance document no: 201412-847 and ethical clearance document no: MU/ECA/48/776 respectively) for the Karachi VASA child mortality study, for which this validated questionnaire was used.

After being informed of the study's purpose and procedures, all participants were asked if they would be willing to participate and informed written consent was obtained. Those who did not wish to participate in the study were given the option to withdraw themselves from the study, and this was done with respect. All responses were treated as strictly confidential. It was ensured that, if a respondent experienced any emotional disturbance during the data collection process, the respondent would be referred to a health professional; however, no such case was encountered.

## Structure of the CHERG's VASA Questionnaire

The CHERG's VASA questionnaire(CHERG, 2018b) is a [illegible]antitative tool for collecting data on the signs and symptoms of illness (including varied BDs) and the relevant history of social

circumstances (NBDs) right from the conception of the mother until the death of the ill child including few open-ended questions i.e., the open narratives (which specifically asks for any additional notes to be mentioned by the respondents in relation to the Verbal autopsy component as well as the summary from the interviewer of the whole case). It also quantitatively captures the signs and symptoms a child encountered during the illness and the social history of the deceased child before the death of the child. The initial pre-validated CHERG VASA questionnaire consisted of VA and SA components having 14 different modules (sections) focusing on the different variables ranging from the demographic profile of the respondent, family of the deceased child, parents of the deceased child, healthcare-seeking process and socioeconomic status to variables encompassing the entire continuum of care during the pregnancy till the death of the child.

After the section on the VASA general information (which collects information on the general variables), the VA component has 06 modules altogether, while the SA component has 07 modules (table 2, columns 1&2). These VA and SA components and relevant modules have been arranged in such an order that as the interview proceeds with the respondent, the sequence of events gets unfolds (table 2, column 3). To record relevant information on relevant age groups (i.e., neonatal deaths, and post-neonatal deaths), specific skip patterns exist in the tool.

| **Table 2: Structure of CHERG's VASA questionnaire** | |
|---|---|
| **Details of different modules of VA & SA components included in the VASA tool** | **Sequence of different modules of VA & SA components in VASA tool** |

| | | | |
|---|---|---|---|
| **VASA General Information:**<br>● Deceased background,<br>● Interview details,<br>● Consent,<br>● Respondent's details,<br>● Details of others present during the interview | **VA Sections:**<br>VA-1: Background<br>VA-2: Maternal History<br>VA-3: Neonatal deaths<br>VA-4: Infants and child deaths<br>VA-5: Health records<br>VA-6 Open-ended questions (any additional information about illness and death) | **SA Sections:**<br>SA-1: Mother and her household<br>SA-2: Social capital<br>SA-3: Maternal history<br>SA-4: Care-seeking for maternal complications<br>SA-5a: Care of the newborn<br>SA-5b: Preventive care of post-neonates<br>SA-6: Care-seeking of child's fatal illness<br>SA-7: Open-ended questions (any additional information about social circumstances) | (VA-1); (SA-3); (VA-2); (SA-4); (SA-5a); (VA-3); (SA-5b); (VA-4); (SA-6); (VA-5); (SA-1); (SA-2); (VA-6) & (SA-7). |

# Translation & Back Translation

The CHERG VASA tool is freely available(CHERG, 2018b). The tool is available either in the integrated version of VASA or in different sections separately. Before validation, the questionnaire was forward translated into the local language,  Urdu, and then back-translated to English based on the **"Brislin Back-translation"** technique(Bennet & Ritchie, 1975). The process involved a professional language translator and a panel of professional bilingual subject experts (paediatricians, neonatologists, gynaecologists, obstetricians, social scientists, and psychologists). The final translated version was processed for the validation process.

# Data collection for the validation study

The respondents (mothers of deceased children) were recruited, and their written consents were obtained. A total of 100 VASA interviews were conducted. Data was recorded on both the platforms i.e., Paper-And-Pencil Interviews (PAPI)(i.e., paper-based VASA tool); and Computer Assisted Personal Interview software (CAPI)(an electronic platform format for data collection with skip patterns using CSPro software application) and were compared to check any inconsistencies. However, no inconsistencies were encountered between the PAPI and CAPI

platforms. The completion rate of the questions on both PAPI and CAPI was the same (100%), however, there were some issues by the interviewee and interviewer on the implementation of these two methods which are elaborated in a qualitative study(Siddiqui et al., 2014) The only difference between the two platforms was the ease of data collection and less time utilized for data collection by the data collectors(Siddiqui et al., 2014). Data quality was checked by a quality management team and researcher. None of the mothers refused or rejected the interview. Extensive training to the data collectors and data entry operators (with a minimum educational background of having Intermediate level (Higher Secondary School Certificate i.e., Grade-12) was given on communication skills, details of the project, questionnaire design and data entry process. Collected data were shared with a panel of subject specialists (five Paediatricians with expertise in Neonatology) trained for PCVA for reviewing the data and assigning the MCoD and SCoD (five sociologists) through VA and SA components of the integrated VASA tool. Medical CoD was assigned by the physicians (five Paediatricians with expertise in Neonatology) (through Physician Certified Verbal Autopsy-PCVA), where the PCVA trained physicians reviewed the mortality case reports (complete VASA data) including signs and symptoms, open narratives, medical history and sequence of events prior to death of deceased children and assigned the most probable MCoD. Initially the data was shared with one reviewer, who assigned a MCoD. Once received, the data was then shared with second reviewer and the level of agreement was checked. In case of any disagreement between the initial two reviewers, a third reviewer was assigned until the agreement between the two different reviewers was achieved. The MCoD assigned by the reviewers were kept confidential by the other reviewers. While conducting PCVA, reviewers were kept blinded with the MCoD assigned on the death certificates of those cases (under-five

mortalities) with a record of death certificates. Similarly, the SCoD were assigned by the social scientists through the same process after reviewing the integrated VASA tool.

# Tool's validation procedure

As illustrated in Table 3 and Fig 1, the CHERG VASA tool was evaluated on its ***validity*** and ***reliability*** through face validation, content validation, discriminant validation, and reliability analysis and the details are described in the respective sections below.

**Table 3: Summary of methodology for validation of CHERG's VASA questionnaire**

| Assessment | Method | Outcome |
|---|---|---|
| **Face Validation** | **Subjective review** of the tool by a panel of experts | The updated questionnaire which was assessed on C.V., D.V., & R.A. (below in this table) |
| **Content Validation (C.V.)** | Calculation of **I-CVI** using agreement level of 05 experts on a dichotomous scale | I-CVI ranged between 81.43% to 96.36%. |
| **Discriminant Validation (D.V.)** | Calculation using **Eigenvalue (E.V.)**, **Canonical correlation (C.C.)**, **Wilk's Lambda (W.L.),** and **Accuracy** | • E.V.=between 0.61 to 735.11<br>• C.C.= between 0.86 and 1.00<br>• W.L.=<0.001<br>• Accuracy of >75.07% |
| **Reliability Analysis (R.A.)** | Calculation using **Cronbach's Alpha (C.A.) statistics** | C.A ranged between 0.843 and 0.973 |

**C.V.**: Content validation; **D.V.**: Discriminant Validation; **R.A.:** Reliability Analysis; **I-CVI:** Item-content Validity Index; **E.V.:** Eigenvalue; **C.C.:** Canonical correlation; **W.L.:** Wilk's Lambda; **C.A.:** Cronbach's Alpha;

**Figure 1: Flow chart of the validation process**

# Results

The sample of 100 responding mothers had the following demographic characteristics (table 4).

**Table 4: Demographic characteristics of the enrolled households for validation study**

| Characteristics | n | % |
|---|---|---|
| **Mothers' characteristics** | | |
| | 100 | 100 |
| Married or living with a man | 97 | 97 |
| Widowed | 3 | 3 |
| **Mother's mean age when first married (years):** | | |
| 15-20 | 8 | 8 |
| 21-24 | 54 | 54 |
| 25-28 | 23 | 23 |

| | | |
|---|---|---|
| 29-32 | 11 | 11 |
| 33-36 | 4 | 4 |
| **Mother years of schooling (mean years of schooling):** | | |
| Didn't attend school | 12 | 12 |
| Primary (1-5 years) | 40 | 40 |
| Middle (6-8 years) | 17 | 17 |
| Secondary (9-10 years) | 26 | 26 |
| Intermediate (11-12 years) | 5 | 5 |
| >12 years | 0 | 0% |
| **Main breadwinner** | | |
| Father | 46 | 46 |
| Mother | 31 | 31 |
| Both | 23 | 23 |
| **Main breadwinner's occupation** | | |
| Farmer/agricultural works | 0 | 0 |
| Poultry or cattle raising | 0 | 0 |
| Domestic servant | 43 | 43 |
| Home-based manufacturing | 29 | 29 |
| Unskilled labourer | 10 | 10 |
| Semi-skilled labourer/service provider | 9 | 10 |
| Factory worker, blue-collar service | 8 | 8 |
| Business owner (shop) | 1 | 1 |
| Professional/technician | 0 | 0 |
| **Type of house (bricked/open-air etc.)** | | |
| Open air/Tents/bush houses | 5 | 5 |
| Asbestos sheet roofed with bricked boundary | 39 | 39 |
| Bricked roofed with bricked boundary | 56 | 56 |
| **Access to toilets** | | |
| No facility/bush /field | 2 | 2 |
| Unimproved facility* | 4 | 4 |
| Shared facility | 6 | 6 |
| Improved facility* | 88 | 88 |
| **Access to electricity** | | |
| Yes | 95 | 95 |
| No | 5 | 5 |
| **Household size** | | |
| **<5** | 24 | 24. |
| **≥5 <10** | 66 | 66 |
| **≥10<15** | 10 | 10 |
| **Use of energy for cooking** | | |
| Gas | 35 | 35 |
| Gas and Kerosine Oil | 43 | 43 |
| Kerosine Oil | 20 | 20 |
| Wood | 3 | 3 |
| **Separate place for cooking** | | |
| Yes | 85 | 85 |
| No | 15 | 15 |
| **Source of drinking water under the utilization by the households** | | |
| Pond water | 5 | 5 |
| Tap water | 48 | 48 |
| Treated water | 47 | 47 |
| **Access to boiled drinking water** | | |
| Yes | 7 | 7 |
| No | 93 | 93 |
| **Birth registration of the existing <5 children** | | |
| Yes | 3 | 3 |
| No | 388 | 97 |
| *Improved sanitation facilities are likely to ensure hygienic separation of human excreta from human contact. They include flush/pour flush (to the piped sewer system, septic tank, pit latrine), ventilated improved pit (VIP) latrine, pit latrine with slab, and composting toilet(WHO/UNICEF, 2015) | | |

# Characteristics of included deceased children

The table 5, mentions the details of some of the important characteristics of the deceased children included in the validation study.

| Table 5: Characteristics of the enrolled deceased under-five children for the validation study | | |
|---|---|---|
| | N | % |
| **Gender of the deceased children** | | |
| **Boy** | **59** | **59** |
| **Girl** | **41** | **41** |
| **Age of deceased children** | | |
| **Stillbirths** | **33** | **33** |
| **Livebirths** | **67** | **67** |
| | | |
| **Place of birth of the deceased children** | | |
| **Government hospital** | **17** | **17** |
| **Private hospital** | **41** | **41** |
| **Home** | **42** | **42** |
| | | |
| **Age at which child died** | | |
| **Born dead** | **67** | **67** |
| **Neonate** | **17** | **17** |
| **1 month-59 month** | **16** | **16** |
| **Frequency of different medical causes of under-five deaths** | | |
| **Preterm delivery (P07)** | **7** | **7** |
| **Birth asphyxia (P21)** | **4** | **4** |
| **Neonatal Pneumonia (P23)** | **2** | **2** |
| **Neonatal Tetanus (A33)** | **2** | **2** |
| **Neonatal Sepsis (P63)** | **1** | **1** |
| **Neonatal Meningitis (G03)** | **1** | **1** |
| **Neonatal Congenital malformations (Q89)** | **0** | **0** |
| **Neonatal Sudden unexplained death (R99)** | **0** | **0** |
| **Neonatal Diarrhoea (A09)** | **0** | **0** |
| **Postneonatal Diarrhoea (A09)** | **5** | **5** |
| **Postneonatal Pneumonia (J22/J18)** | **4** | **4** |
| **Postneonatal Severe Malnutrition (D64)** | **3** | **3** |
| **Postneonatal Hemorrhagic fever (A99)** | **2** | **2** |
| **Postneonatal Meningitis (E46)** | **1** | **1** |
| **Postneonatal Malaria (B54)** | **1** | **1** |
| **Mother's hospitalization** | | |
| **Yes** | | |
| **No** | | |
| **Place of birth** | | |
| **Government hospitals** | **46** | **46** |
| **Private hospitals** | **37** | **37** |
| **Home** | **17** | **17** |
| **Antenatal history** | | |
| **Attended at least antenatal visit in HCF** | | |
| **1 visit** | **6** | **6** |
| **2 visits** | **24** | **24** |
| **3 visits** | **14** | **14** |
| **4 visits** | **16** | **16** |
| **No visit** | **40** | **40** |
| **Child's hospitalization** | | |
| **Yes** | **11** | **11** |
| **No** | **89** | **89** |
| **Child's hospital records** | | |
| **Yes** | **31** | **31** |
| **No** | **69** | **69** |
| **Which health care provider was consulted by mother during ANC** | | |

| | | |
|---|---|---|
| **Did not see any HCP for ANC** | **41** | **41** |
| **Local dai** | **46** | **46** |
| **Private clinic** | **13** | **13** |
| **HCP: Healthcare professional, ANC: Antenatal care** | | |

## (a) Face validity assessment

For assessing the face validity of the tool, the questionnaire was reviewed and critically evaluated by a panel of 6 experts—a researcher and 5 expert(s) in the relevant field of interest (e.g., paediatrician, gynaecologist, obstetrician, social scientist, and psychologist(Lebet, Asaro, Zuppa, & Curley, 2018; Trockel et al., 2018) on the tool's format to find out, whether all the sections and items be easily readable, clear, and straightforward to understand and comprehend by the interviewees and respondents; and on the need of rephrasing, omitting, or adding any questions. The panellists found no need for any modifications in terms of rephrasing questions, omitting any option of any item, adding an option of any item, etc. The team identified that the items of the tool were phrased in such a way that the respondent would need to think before responding, thereby minimizing the chances of response bias. The translated questionnaire which went for face validation was then named **"VASA Questionnaire-version-1".** This version of the questionnaire was utilized for content validation as well as to collect data for undertaking discriminant validation.

## (b) Content Validity assessment

It involved a panel of five subject specialists (paediatrician, gynaecologists, obstetrician, social scientist, and psychologist) who gave their ratings on different parameters (especially on the comprehensiveness of the tool and the relativity of the items on the tool with the background

conceptual framework of the topic). Each member from a panel was requested to rate every item on the VASA Questionnaire-version-1 on a 5-point Likert scale based on the relevancy to the conceptual framework (1=not relevant, 2=somewhat relevant, 3=relevant, 4=quite relevant, 5=highly relevant). The ratings were calculated using **Item-content Validity Index (I-CVI)**. For each item, the I-CVI was computed as the number of experts who rated an item on the top two boxes (i.e., quiet relevant or highly relevant), divided by the total number of experts. The level of agreement between experts should be as high as possible to make the I-CVI acceptable. The data on content validation is presented in Table 6 under different themes/constructs. These themes/constructs were clustered either as pre-birth themes/constructs or post-birth themes/constructs, sections 1 through 8 cover pre-birth constructs, and sections 9 through 16 cover during and post-birth constructs. The average rating of each section was more than 4 (on a scale of 1 to 5), and the I-CVI is more than 80% for all the sections of the VASA questionnaire. Relatively lower I-CVI was observed for *"symptoms before pregnancy"* (81.43%) and *"Birth History of Child"* (81.82%) sections.

**Table 6: Average expert rating (from five homologous panels of five different professional expert groups) and overall Item-content Validity Index (I-CVI) by sections of CHERG's VASA questionnaire**

| **VASA questionnaire themes** | **Content Validity (C.V.) (average rating)** | | | | | **Average I-CV (%)I** |
|---|---|---|---|---|---|---|
| | **Rater 1 (n=5)** | **Rater 2 (n=5)** | **Rater 3 (n=5)** | **Rater 4 (n=5)** | **Rater 5 (n=5)** | |
| **Content validation (C.V.) of pre-birth themes/constructs* of VASA questionnaire** | | | | | | |
| Demographic and social constructs | 4.56 | 4.44 | 4.47 | 4.53 | 4.53 | 92.94 |
| Symptoms before pregnancy | 4.43 | 4.21 | 4.14 | 4.21 | 4.29 | 81.43 |
| Symptoms during initial two trimesters | 4.45 | 4.24 | 4.27 | 4.24 | 4.36 | 82.42 |
| Symptoms during last trimesters | 4.38 | 4.38 | 4.38 | 4.38 | 4.38 | 87.50 |
| Care Seeking behaviour for symptoms of last trimester | 4.64 | 4.45 | 4.50 | 4.45 | 4.50 | 96.36 |
| Symptoms during labor and care-seeking behaviour | 4.50 | 4.30 | 4.40 | 4.40 | 4.50 | 88.00 |
| Treatment of labor symptoms and post-delivery | 4.34 | 4.16 | 4.19 | 4.22 | 4.28 | 83.75 |
| Knowledge Attitude and Behavior on maternal symptoms | 4.53 | 4.39 | 4.47 | 4.43 | 4.49 | 88.98 |
| **Content validation (C.V.) of during & post-birth themes/constructs* of VASA questionnaire** | | | | | | |
| Birth History of Child | 4.41 | 4.27 | 4.27 | 4.23 | 4.36 | 81.82 |
| Physical Malformations Of Child's Body | 4.57 | 4.43 | 4.36 | 4.43 | 4.50 | 88.57 |
| Child Illness Details | 4.31 | 4.17 | 4.14 | 4.17 | 4.21 | 82.07 |

| | | | | | | |
|---|---|---|---|---|---|---|
| Health Seeking Behavior Of Child Illness | 4.63 | 4.50 | 4.56 | 4.56 | 4.56 | 95.00 |
| Health care professionals (HCP) involved during Child Illness | 4.46 | 4.21 | 4.36 | 4.32 | 4.36 | 86.43 |
| Services Cost Burden and Delays | 4.50 | 4.21 | 4.29 | 4.29 | 4.43 | 91.43 |
| Preventive Measures Between Child Birth and Illness | 4.68 | 4.55 | 4.64 | 4.55 | 4.68 | 92.73 |
| Community Efforts during child illness | 4.42 | 4.21 | 4.26 | 4.26 | 4.32 | 85.26 |
| Cause of death-Medical | 4.31 | 4.17 | 4.14 | 4.17 | 4.21 | 82.07 |
| Cause of death-Social | 4.53 | 4.39 | 4.47 | 4.43 | 4.49 | 88.98 |
| *Based on a thorough review of the questionnaire, the content of the VASA questionnaire can be divided into two clusters, sections 1 through 8 covers pre-birth constructs and sections 9 through 16 covers during and post-birth constructs. **C.V.**: Content validation; **I-CVI:** Item-content Validity Index; | | | | | | |

## (c) Discriminant validity (construct validity) assessment

It shows the distinctiveness of different constructs within the questionnaire and helps to identify whether the items on the questionnaire that are not supposed to be related to each other are unrelated or not. It is one of the types of construct validity. The **"VASA Questionnaire-version-1"** (after the face validation exercise) of the tool was utilized for data collection from the respondents who fall within the inclusion and exclusion criteria. All the mothers identified with a recent history of child mortality, and none of the mothers refused or rejected the interview. Collected data were shared with verbal autopsy reviewers (physicians). The reviewers used their clinical reviewing expertise (a process called *"Physicians certified VA technique-PCVA"*) in assigning the medical cause of death. The complete data (including the CoD assigned by the physicians) were computed on SPSS (version 21) and R for the validation process. For pre-birth constructs, *"birth status of the child"* and for during and post-birth constructs *"diseases or conditions directly leading to death"* are the decisive elements with potential discriminating power. Clear clusters and grouping in response patterns on VASA elements were found for the pre-birth constructs. Therefore, valid response differences between the live birth group and stillbirth group (on the elements of each section) were used for the discriminant validity of the pre-birth constructs of the tool. Similarly, valid response differences among different diseases or

conditions directly leading to death were used for the discriminant validity of during or post-birth constructs of the tool.

For pre-birth constructs of the questionnaire, discriminant analysis was performed using birth status as a dependent variable, which was defined in such a way that birth status was coded as "1" (one) in case of stillbirth and "0" (zero) in case of live birth, and elements of each section as discriminating or classification variables. The **goodness of fit summary of discriminant analysis models**, such as eigenvalue (E.V.) of canonical discriminant function, canonical correlation (C.C.), and Wilks' Lambda (W.L.) statistics was used for the discriminant validity measures of the constructs. To analyze the discriminant validity of post-birth constructs of the VASA questionnaire, **multinomial logistic regression** was performed using "disease or condition directly leading to death" as dependent variables and elements of each section of the questionnaire as explanatory variables. Likelihood-Ratio (LR) test and Pseudo R-Square measures (Cox and Snell, Nagelkerke, and McFadden) were used as discriminant validity measures of the constructs.

Out of 100 cases, we had 33 stillbirths and 67 live birth cases. For the analysis, unequal prior probabilities of stillbirth and live birth were assumed and assigned prior probabilities as per the group size in the data. The deaths included in the study were both, i.e. home-based and institutional (formal and informal health care facilities). The Backward variable elimination method was used to compute the discriminant analysis model. It is vital to comment here at this point that the variables which are not included in the final model do not necessarily mean they are not important, but it means these variable(s) do not provide any additional statistical evidence for the classification of live and stillbirth.

The goodness of fit summary of discriminant analysis models of each of the pre-birth constructs of the VASA questionnaire is presented in Table 7. The **eigenvalue (E.V.)** of canonical discriminant function is *the ratio of the between-groups sum of squares to within groups sum of squares*. A high value of eigenvalue indicates that the canonical function explains sufficiently higher variation in data. Similarly, the **canonical correlation (C.C.)** coefficient shows the predictive ability of the canonical discriminant function. The value of C.C. ranges from 0 to 1, with a value closer to 1 showing the strong predictive ability of canonical discriminant function. The third goodness of fit statistics i.e., **Wilks' Lambda** (ranging from 0 to 1), was calculated to be the proportion of the total variance in the discriminant scores not explained by differences among the groups. A small value of W.L. and statistically significant (p-value of <0.05) shows a good fit of the discriminant model.

The goodness of fit summary of multinomial logistic regression models of each of during & post-birth constructs of the VASA questionnaire is presented in Table 7. Statistically significant **Likelihood-Ratio (LR) test** result infers statistically significant improvement over the null model. **Pseudo R-Square measures (Cox and Snell, Nagelkerke, and McFadden)** range from 0 to 1, with a value closer to "1" (one) indicating a good model fit.

**Table 7: Discriminant validation of CHERG's VASA questionnaire**

**The goodness of fit summary of discriminant analysis models for 'pre-birth constructs'**

| VASA questionnaire constructs and sections | Eigenvalue | Canonical Correlation | Wilks' Lambda | | |
|---|---|---|---|---|---|
| Sections | | | Wilks' Lambda | Chi-square | p-value |
| Demographic and social constructs | 37.44 | 0.987 | 0.026 | 339.37 | <0.001 |
| Symptoms before pregnancy | 2.76 | 0.86 | 0.27 | 127.74 | <0.001 |
| Symptoms during initial two trimesters | 2.76 | 0.86 | 0.27 | 127.74 | <0.001 |
| Symptoms during last trimester | 91.13 | 0.871 | 0.241 | 422.91 | <0.001 |
| Care Seeking behavior for symptoms of last trimester | 1700.51 | 0.615 | 0.621 | 278.97 | <0.001 |
| Symptoms during labor and care seeking behavior | 120.88 | 0.92 | 0.154 | 446.68 | <0.001 |
| Treatment of labor symptoms and post delivery | 1042.16 | 0.993 | 0.013 | 646.35 | <0.001 |
| Knowledge Attitude and Behavior on maternal symptoms | 1475.99 | 0.952 | 0.093 | 682.34 | <0.001 |

| The Goodness of fit summary of multinomial logistic regression models for 'during and post-birth constructs' | | | | |
|---|---|---|---|---|
| **Sections** | **LR Tests (p-value)** | **Cox and Snell** | **Nagelkerke** | **McFadden** |
| Birth of Child | <0.001 | 0.968 | 0.996 | 0.963 |
| Physical Malformations of Child's Body | <0.001 | 0.968 | 0.996 | 0.963 |
| Child Illness Details | <0.001 | 0.968 | 0.996 | 0.963 |
| Health Seeking Behavior of Child Illness | <0.001 | 0.968 | 0.996 | 0.963 |
| Health care professionals (HCP) involved during Child Illness | <0.001 | 0.968 | 0.996 | 0.963 |
| Services Cost Burden and Delays | <0.001 | 0.956 | 0.983 | 0.87 |
| Preventive Measures between childbirth and illness | <0.001 | 0.968 | 0.996 | 0.963 |
| Community Efforts during child illness | <0.001 | 0.956 | 0.983 | 0.87 |
| Cause of death-Biological | <0.001 | 0.956 | 0.963 | 0.963 |
| Cause of death-Social | <0.001 | 0.964 | 0.985 | 0.89 |
| **C.V.**: Content validation; **D.V.**: Discriminant Validation; **R.A.:** Reliability Analysis; **I-CVI:** Item-content Validity Index; **E.V.:** Eigenvalue; **C.C.:** Canonical correlation; **W.L.:** Wilk's Lambda; **C.A.:** Cronbach's Alpha; | | | | |

A high eigenvalue of canonical discriminant function (>1), a high value of canonical correlation (>0.8), and a statistically significant Wilks' Lambda, with a p-value of <0.001, validate the good fit of discriminant models for pre-birth constructs of VASA questionnaire. A high value of Pseudo R-Square measures (>0.8) and a statistically significant LR test, with a p-value <0.001, validate the good fit of multinomial logistic regression models for during & post-birth constructs of the VASA questionnaire. Hence, underlying constructs of the VASA questionnaire, in all sections (Table 7), were found to be distinctive and well-established to address the conceptual framework. More than 85.07% accuracy of the discriminant functions and the multinomial logistic regression model was observed in all sections of the VASA questionnaire.

## (d) Reliability assessment

Reliability analysis measures the tool's capacity to provide a consistent result. Use of **Cronbach's Alpha** statistics was utilized to measure the reliability of constructs. Due to the dominantly dichotomous nature of data, the calculation of Cronbach's Alpha based on the Pearson correlations matrix would be incorrect or potentially very biased. Therefore, "hetcor"

function of “polycor” package of R was used to compute the appropriate heterogeneous correlation matrix. The ‘hetcor’ function is capable of calculating Pearson correlations (for numeric data), polyserial correlations (for numeric and ordinal data), and polychoric correlations (for ordered or non-ordered factors). Reliability for the different sections of the VASA questionnaire is presented in Table 7. The universal rule of thumb is that, if the value of **Cronbach's alpha** (C.A.) is 0.70 and more, the reliability is good, if the value of C.A. is between 0.80 and 0.90 the reliability is better, and if the value is 0.90 and beyond, the reliability is best. In our study, the Cronbach's Alpha values are above 0.90 across all the constructs of pre-birth, during & post-birth constructs of the VASA questionnaire (except “Demographic and social constructs” and “Care Seeking behaviour for symptoms of last trimester” which are 0.843 and 0.876 respectively)(Table 8).

Classification of sensitivity, specificity, and accuracy of the models developed based on the elements of different sections of the VASA questionnaire are presented in Table 8 below. 100% specificity, 100% sensitivity and 100% accuracy of the discriminant functions in classifying births to live and stillbirths were observed in all sections, except, symptoms before pregnancy and symptoms during the initial two-trimester sections of the VASA questionnaire. For which 100%, 72.73% and 91% of specificity, sensitivity, and accuracy were observed, and for these two sections lowest content validity was observed (see Table 8). Cronbach's Alpha values were found higher than 0.80, indicating the reliability and internal consistency of the underlying constructs.

**Table 8: Reliability analysis of CHERG’s VASA questionnaire**

| VASA questionnaire constructs and section | Specificity (%) | Sensitivity (%) | Accuracy (%) | Cronbach's Alpha |
|---|---|---|---|---|
| **Reliability accuracy of discriminant analysis models for pre-birth constructs** | | | | |
| Demographic and social constructs | 100.00 | 100.00 | 100.00 | 0.843 |

| | | | | |
|---|---|---|---|---|
| Symptoms before pregnancy | 100.00 | 72.73 | 91.00 | 0.909 |
| Symptoms during initial two trimesters | 100.00 | 72.73 | 91.00 | 0.944 |
| Symptoms during last trimester | 88.00 | 100.00 | 92.00 | 0.894 |
| Care Seeking behaviour for symptoms of last trimester | 76.00 | 100.00 | 85.00 | 0.876 |
| Symptoms during labor and care-seeking behaviour | 100.00 | 100.00 | 100.00 | 0.954 |
| Treatment of labor symptoms and post delivery | 100.00 | 100.00 | 100.00 | 0.933 |
| Knowledge Attitude and Behavior on maternal symptoms | 100.00 | 100.00 | 100.00 | 0.940 |
| **Reliability and accuracy of multinomial logistic regression models for during and post-birth constructs** | | | | |
| Birth of Child | 100.00 | 100.00 | 100.00 | 0.907 |
| Physical Malformations of Child's Body | 100.00 | 24.20 | 75.00 | 0.947 |
| Child Illness Details | 100.00 | 100.00 | 100.00 | 0.948 |
| Health Seeking Behavior of Child Illness | 100.00 | 100.00 | 100.00 | 0.945 |
| Health care professionals (HCP) involved during Child Illness | 100.00 | 100.00 | 100.00 | 0.928 |
| Services Cost Burden and Delays | 100.00 | 100.00 | 100.00 | 0.95 |
| Preventive Measures between childbirth and illness | 100.00 | 100.00 | 100.00 | 0.973 |
| Community Efforts during child illness | 100.00 | 100.00 | 100.00 | 0.942 |
| Cause of death-Biological | 100.00 | 100.00 | 100.00 | 0.953 |
| Cause of death-Social | 100.00 | 100.00 | 75.00 | 0.973 |

# Discussion

The literature shows a lack of evidence on validation studies for integrated VASA tools for under-five years children. Globally, no research has been done on identifying the validity and reliability of CHERG's VASA questionnaire in diagnosing under-five children who died in a community-based household survey. Although the CHERG’s integrated VASA tool was developed with a sound technical methodology and is based on the integration of several models and theories including the Three Delay model, and “The Pathway to Survival Conceptual Framework” (TPtoSCF)(Siddiqui et al., 2018),(CHERG, 2018a), still its validation for developing countries like Pakistan (where the huge estimates of under-five mortality lie) was technically required. After this current study, this validated tool will ensure the availability of a validated VASA tool that may give valid and reliable data pertaining to child mortality determinants operating in Pakistan. Such a tool was highly required to overcome the data

shortage-related issues in the country, where the death registration is highly compromised(Abouzahr et al., 2014; Siddiqui et al., 2018; Siddiqui, Ng, & Low, 2016). When it comes to the reasons for having scanty data on death registration, one of the potential bottlenecks is the delayed and compromised notification of child deaths to the National Civil Registration and Vital Statistics Agency (NCRVS). Because the process of notification is time-consuming, there is no evidence of strict compliance among the general population in death notification(Abouzahr et al., 2014; Asad Mahmood Alvi, 1993). There are no financial benefits or assistance being provided to the community. The death registration process in the NCRVS is severely hampered, and this is true at all levels, from the local community to the national level. The existing data is also very limited and with questionable validity and accuracy(Abouzahr et al., 2014; Siddiqui et al., 2018; Siddiqui, Ng, & Low, 2016), this is particularly due to no implementation of standardized methods of data collection and reporting for death notification(Abouzahr et al., 2014; Zakar et al., 2018). Utilization and implementation of this validated tool in identifying mortality-related determinants and in assigning medical and social causes of child deaths, especially of those deaths which have been missed from the National Civil Registration and Vital Statistics will surely help us in overcoming data-related issues pertaining to child mortalities.

Validation studies usually encounter daunting challenges. Most of them include recall biases, information biases, small sample size estimation, etc. To overcome such challenges, the methodology involved in the validation studies should be very concrete and categoric, ensuring that the responses from the validated tool should be able to give information with acceptable accuracy. It is highly essential to undertake such relevant validation studies in developing

countries so that it will make the validated version of the VASA tool available for conducting VASA investigations.

Literature shows that there are several versions of VA tools developed by multiple agencies and research teams. Literature reflects several attempts at validation studies of different VA tools that have utilized different methodologies to overcome such challenges and identified the precise CoD with much accuracy(Kahn et al., 1999; Mpimbaza et al., 2015; Murray et al., 2014; Rodriguez et al., 1998). The most commonly used VA tool which underwent validation is the World Health Organization’s (WHO) VA tool(Aggarwal et al., 2013),(Nichols et al., 2018).

Having precisely calculated and appropriate sample size, the results of any validation study show the true validity and reliability of the tool. Compared to our VASA validation study, which included 100 deceased under-five cases, all the other VA validation studies undertook validation on a comparatively larger sample size and implemented only the VA component(Aggarwal et al., 2013; Marsha et al., 2003; Nausheen et al., 2013; Soofi et al., 2015). We feel that having a smaller sample size is one of the limitations of our validation study. However, our sample size is purely based on general recommendations from Hertzog(Hertzog, 2008) (this was purely due to the reason that there are no specific guidelines for the sample size of respondents required for pre-survey validation of the VASA tool, hence this study followed the general guidelines from Hertzog guidelines), which suggests that the minimum sample size for any validation study should be 20% of the sample size calculated for the survey the tool will be utilized for. Aggarwal et al.(Aggarwal et al., 2013) included 313 neonatal deaths; Nausheen et al.(Nausheen et al., 2013) undertook validation on 204 neonatal deaths; the sample size in Soofi et al.(Soofi et al., 2015) was 626 neonatal deaths, and Marsha et al.(Marsha et al., 2003) used 137 neonatal deaths.

Conducting interviews after a long delay subsequent to the death event may affect the quality and reliability of data. This is most likely because the respondent needs to recall the events and happenings of different events that occurred before and around the death event. It is highly recommended that VASA interviews should be undertaken somewhere between 01 months till 06 months provided the family has come out of the mourning period(Serina et al., 2016). Conducting interviews within the mourning period may cause distress and influence the respondent's disposition and ability to engage in a VASA interview(Serina et al., 2016). P Serina et al.(Serina et al., 2016), suggest that a long recall period may limit the respondent's ability in recalling and recollecting pertinent facts. Events (or symptoms) with extraordinary severity (or implications) remain in the recall for a much more extended period compared to those with mild to moderate severity(Serina et al., 2016). Likewise all the other studies(Dikid et al., 2013; Marsha et al., 2003; Nausheen et al., 2013; Soofi et al., 2015), our VASA validation study undertook interviews within six (06) weeks after the mortality event. In our sample size, none of the respondents had any difficulty recalling the events.

The capacity of any questionnaire to correctly assign the proper CoD to any mortality with a degree of precision is called accuracy. When dealing with individual-level data, the diagnostic accuracy of the VASA tool is considered to be satisfactory when the specificity and sensitivity of the questionnaire are a minimum of 90%(Soleman et al., 2006). However, at a much larger, i.e. national level, if the validation attempt of the VASA tool shows sensitivity and specificity of at least 50% and 90% respectively, the tool is usually considered to be having acceptable diagnostic accuracy(Soleman et al., 2006). This study found an accuracy of between 97% and 100% in assigning medical and social causes of child deaths respectively. In comparison, the accuracy of the WHO's VA tool validated in Aggarwals' study(Aggarwal et al., 2013) in identifying

different neonatal causes of mortalities was found to be up to between 78% to 92% (except for birth asphyxia-16%); however, the kappa statistics was moderate from 0.46 to 0.55. Similarly, in Soofi et al. (Soofi et al., 2015) the WHO VA tool showed a sensitivity of more than 83.5% for diagnosing different causes of neonatal mortality (except congenital malformation, which was 57%), while the specificity of all the major causes of neonatal deaths was found to be more than 90%. Marsha et al.(Marsha et al., 2003) found the sensitivity of the process of assigning the cause of death ranging from 39% (in diagnosing infection) to 90% (in diagnosing causes related to *too early/too small syndrome*) and specificity ranging from 67% (in diagnosing infection) and 99% (in diagnosing Neonatal Tetanus).

## Strengths of our validation study

Our study is first in its kind as it validates the CHERG's integrated VASA tool, which is based on the most holistic conceptual framework, i.e. TPtoSCF (that addresses the barriers and limitations involved in accessing health care services), that have the capacity to record information required to develop policy in preventing under-five death incidents and improving the child survival estimates in developing countries (like Pakistan) where an unknown, but a large number of child mortality events have known to be missed and their medical causes have not been assigned.

Moreover, this study is important as it attempted a detailed validation of CHERG's integrated VASA tool by incorporating rigorous methodology and utilizing almost all the core methods suggested for identifying the validity and reliability evidence of any questionnaire(Taherdoost, 2018). The validation exercise yielded high I-CVI (>81.43%), with Cronbach's Alpha reliability statistics being 0.843 for *"Demographic and social constructs"* and highest of 0.973 for

*"Preventive Measures between childbirth and illness"*; the accuracy of between 81% (for *"Care seeking behaviour for symptoms of last trimester")* and 100% of the discriminants classifying births to live and stillbirths. The tool showed an accuracy of between 97% and 100% in assigning medical and social causes of child deaths respectively.

Although different VA questionnaires have been validated in Pakistan, however, some of them are specifically designed for the neonatal population(Soofi et al., 2015), while others are for stillbirths(Nausheen et al., 2013). The CHERG's VASA integrated questionnaire encompasses three age groups and has not been validated for the Pakistani population.

An electronic CAPI (Computer Assisted Personal Interview) notebook format for data collection with skip patterns using CSPro software application has also been developed for this validated tool which was utilized for data collection during the Karachi VASA child mortality study. The primary purpose for transforming the lay paper-based form into a notebook application was to minimize the possibility of data collection and data entry errors. No data collection and data entry errors were encountered possibly due to extensive training of the field staff on the data collection and entry.

# Conclusion

Based on our results, we confirm that the CHERG VASA integrated questionnaire is valid, reliable, and relevant to the conceptual frameworks for the Pakistani Population. The validated CHERG VASA tool can be utilized for establishing medical and social causes of Under-five child deaths in Karachi, Pakistan, and is one of the assets for Pakistan's child health policy with

the potential to record relevant data and assign causes of child mortality cases occurring in Pakistan. The evidence extracted from the data and information obtained from the validated VASA tool will surely complement healthcare professionals and policymakers in improving the practice and modifying the policy for enhancing the survival of under-five children in Karachi, Pakistan.

## Authors' contribution

Conceptualization: M.B. Siddiqui

Methodology: M.B. Siddiqui

Implementation: M.B. Siddiqui

Software development: M.B. Siddiqui

Data collection: M.B. Siddiqui

Data entry: M.B. Siddiqui

Ensuring data completeness: M.B. Siddiqui

Analysis: M.B. Siddiqui

Writeup: M.B. Siddiqui

Writing– original draft: M.B. Siddiqui,

MBS, was involved in all aspects of this study, ranging from conceptualization, methodology, implementation, software development, data collection, data entry, ensuring data completeness, analysis, write up (original draft). MBS undertook complete biostatistical analyses, which was proofread by KA. CWN & WYL proofread the writeup. All authors have read and approved the final manuscript.

## Acknowledgement

We would like to acknowledge the key personnel from the relevant communities for assisting the data collection process. SAR participated in the proofreading of some sections of this manuscript. We also would like to acknowledge the efforts of Dr Muhammad Akhter Hamid (MAH) who contributed to PCVA. We would like to inform that an unreviewed preprint of this manuscript is already available on Research Square at the link (https://www.researchsquare.com/article/rs-35489/v1) and Europe PMC (https://europepmc.org/article/ppr/ppr191893) with DOI (10.21203/rs.3.rs-35489/v1).

## List of abbreviations

**C.A.:** Cronbach's Alpha

**C.C.:** Canonical correlation

**CHERG:** Child Health Epidemiology Reference Group

**CoD:** Cause of Death

**CRVS:** Civil Registration and Vital Statistics

**C.V.**: Content validation

**D.V.**: Discriminant Validation

**E.V.:** Eigenvalue

**HMIS:** Health Management Information System

**I-CVI:** Item-content Validity Index

**MCoD:** Medical Cause of Death

**PCVA:** Physicians certified VA technique

**R.A.:** Reliability Analysis

**SA:** Social Autopsy

**SCoD:** Medical Cause of Death

**SPSS:** Statistical Package for Social Sciences

**TPtoSCF:** The Pathway to Survival Conceptual Framework

**VA:** Verbal Autopsy **LR:** Likelihood-Ratio (LR) test

**VASA:** Verbal autopsy/Social Autopsy

**WHO:** World Health Organization

**W.L.:** Wilk's Lambda

# Declarations

## Consent for publication

Not applicable. (This research does not include any data from an individual person.)

## Availability of data and materials

The datasets used and analyzed during the current study are available at https://figshare.com/s/883fe45ea7da71483acd.

## Competing interests

The author(s) declare(s) that they have no competing interests.


## Funding

The current study has no funding attached.

# References

Abouzahr, C., Azimi, S. Y., Bersales, L. G. S., Chandramouli, C., Hufana, L., Khan, K., Kulkayeva, G., Marskell, J., & Sauyekenova, L. (2014). Strengthening civil registration and vital statistics in the Asia-Pacific region: Learning from country experiences. *Asia-Pacific Population Journal*, *29*(1), 39–73.

Afzal, U., & Yusuf, A. (2013). The State of Health in Pakistan: An Overview. *The Lahore Journal of Economics*, *18*, 233–247. https://doi.org/http://www.lahoreschoolofeconomics.edu.pk/EconomicsJournal/LJEIntro.aspx

Aggarwal, A. K., Kumar, P., Pandit, S., & Kumar, R. (2013). Accuracy of WHO Verbal Autopsy Tool in Determining Major Causes of Neonatal Deaths in India. *PLoS ONE*, *8*(1). https://doi.org/10.1371/journal.pone.0054865

Anthoine, E., Moret, L., Regnault, A., Sbille, V., & Hardouin, J. B. (2014). Sample size used to validate a scale: A review of publications on newly-developed patient reported outcomes measures. *Health and Quality of Life Outcomes*, *12*(1), 2. https://doi.org/10.1186/s12955-014-0176-2

Asad Mahmood Alvi, B. (1993). *National Registration System in Pakistan Director General of Registration Government of Pakistan*. https://unstats.un.org/unsd/demographic/meetings/wshops/1993_China_CRVS/docs/1993_Doc.11_Pakistan.pdf

Bennet, A. E., & Ritchie, K. (1975). *Questionnaires in medicine A guide to their design and use*. http://trove.nla.gov.au/work/11045163?selectedversion=NBD793226

Bensaïd, K., Yaroh, A. G., Kalter, H. D., Koffi, A. K., Amouzou, A., Maina, A., & Kazmi, N. (2016). Verbal/Social Autopsy in Niger 2012-2013: A new tool for a better understanding of the neonatal and child mortality situation. *Journal of Global Health*, *6*(1), 10602. https://doi.org/10.7189/jogh.06.010602

CHERG. (2018a). *Projects: CHERG Verbal and Social Autopsy Survey Tools and Information*. http://cherg.org/projects/pathways.html#socialautopsy

CHERG. (2018b). *Projects: CHERG Verbal and Social Autopsy Survey Tools and Information*. CHERG. http://cherg.org/projects/pathways.html#socialautopsy

Dikid, T., Gupta, M., Kaur, M., Goel, S., Aggarwal, A. K., & Caravotta, J. (2013). Maternal and Perinatal Death Inquiry and Response project implementation review in India. *Journal of Obstetrics and Gynecology of India*, *63*(2), 101–107. https://doi.org/10.1007/s13224-012-0264-3

Gouda, H. N., Flaxman, A. D., Brolan, C. E., Joshi, R., Riley, I. D., AbouZahr, C., Firth, S., Rampatige, R., & Lopez, A. D. (2017). New challenges for verbal autopsy: Considering the ethical and social implications of verbal autopsy methods in routine health information systems. In *Social Science and Medicine*. https://doi.org/10.1016/j.socscimed.2017.05.002

Hertzog, M. A. (2008). Considerations in determining sample size for pilot studies. *Research in Nursing and Health*, *31*(2), 180–191. https://doi.org/10.1002/nur.20247

Hildenwall, H., Tomson, G., Kaija, J., Pariyo, G., & Peterson, S. (2008). "I never had the money for blood testing" - Caretakers' experiences of care-seeking for fatal childhood fevers in rural Uganda - A mixed methods study. *BMC International Health and Human Rights*, *8*.

https://doi.org/10.1186/1472-698X-8-12
IGME, U. (2020). *Levels & Trend in Child Mortality: Estimates Developed by the Estimates developed by the UN Inter-agency Group for UN Inter-agency Group for Child Mortality Estimation Child Mortality Estimation*. UNICEF.
Kagabo, D. M., Kirk, C. M., Bakundukize, B., Hedt-Gauthier, B. L., Gupta, N., Hirschhorn, L. R., Ingabire, W. C., Rouleau, D., Nkikabahizi, F., Mugeni, C., Sayinzoga, F., & Amoroso, C. L. (2018). Care-seeking patterns among families that experienced under-five child mortality in rural Rwanda. *PLoS ONE*, *13*(1). https://doi.org/10.1371/journal.pone.0190739
Kahn, K., Tollman, S. M., Garenne, M., & Gear, J. S. S. (1999). Who dies from what? Determining cause of death in South Africa's rural north-east. *Tropical Medicine and International Health*, *4*(6), 433–441. https://doi.org/10.1046/j.1365-3156.1999.00415.x
Källander, K., Counihan, H., Cerveau, T., & Mbofana, F. (2019). Barriers on the pathway to survival for children dying from treatable illnesses in Inhambane province, Mozambique. *Journal of Global Health*, *9*(1), 1–14. https://doi.org/10.7189/JOGH.09.010809
Källander, K., Hildenwall, H., Waiswa, P., Galiwango, E., Peterson, S., & Pariyo, G. (2008). Delayed care seeking for fatal pneumonia in children aged under five years in Uganda: a case-series study .الرتجمة العربية لهذه الخالصة يف نهاية النص الكامل لهذه املقالة. *Bulletin of the World Health Organization*, *86*(5), 332–338. https://doi.org/10.2471/BLT.07.049353
Källander, K., Kadobera, D., Williams, T. N., Nielsen, R. T., Yevoo, L., Mutebi, A., Akpakli, J., Narh, C., Gyapong, M., Amu, A., & Waiswa, P. (2011). Social autopsy: INDEPTH Network experiences of utility, process, practices, and challenges in investigating causes and contributors to mortality. *Population Health Metrics*, *9*. https://doi.org/10.1186/1478-7954-9-44
Kalter, H. D., Roubanatou, A. M., Koffi, A., & Black, R. E. (2015). Direct estimates of national neonatal and child cause-specific mortality proportions in Niger by expert algorithm and physician-coded analysis of verbal autopsy interviews. *Journal of Global Health*, *5*(1), 10415. https://doi.org/10.7189/jogh.05.010415
Kalter, H. D., Salgado, R., Babille, M., Koffi, A. K., & Black, R. E. (2011). Social autopsy for maternal and child deaths: A comprehensive literature review to examine the concept and the development of the method. *Population Health Metrics*, *9*(45). https://doi.org/10.1186/1478-7954-9-45
Kalter, H., Yaroh, A. G., Maina, A., Koffi, A. K., Bensaïd, K., Amouzou, A., & Black, R. E. (2016). Verbal/social autopsy study helps explain the lack of decrease in neonatal mortality in Niger, 2007-2010. *Journal of Global Health*, *6*(1). https://doi.org/10.7189/jogh.06.010604
Koffi, A. K., Kalter, H. D., Loveth, E. N., Quinley, J., Monehin, J., & Black, R. E. (2017). Beyond causes of death: The social determinants of mortality among children aged 1-59 months in Nigeria from 2009 to 2013. *PLoS ONE*, *12*(5), e0177025. https://doi.org/10.1371/journal.pone.0177025
Koffi, A. K., Libite, P., Moluh, S., Wounang, R., & Kalter, H. D. (2015). Social autopsy study identifies determinants of neonatal mortality in Doume, Nguelemendouka and Abong–Mbang health districts, Eastern Region of Cameroon. *Journal of Global Health*, *5*(1). https://doi.org/10.7189/jogh.05.010413
Koffi, A. K., Maina, A., Yaroh, A. G., Habi, O., Bensaïd, & Kalter, H. D. (2016). Social determinants of child mortality in Niger: Results from the 2012 National Verbal and Social Autopsy Study. *Journal of Global Health*, *6*(1), 10603.

https://doi.org/10.7189/jogh.06.010603
Koffi, A. K., Mleme, T., Nsona, H., Banda, B., Amouzou, A., & Kalter, H. D. (2015). Social autopsy of neonatal mortality suggests needed improvements in maternal and neonatal interventions in Balaka and Salima districts of Malawi. *Journal of Global Health*, *5*(1), 010416. https://doi.org/10.7189/jogh.05.010416
Koffi, A. K., Perin, J., Kalter, H. D., Monehin, J., Adewemimo, A., & Black, R. E. (2019). How fast did newborns die in Nigeria from 2009-2013: a time-to-death analysis using Verbal /Social Autopsy data. *Journal of Global Health*, *9*(2), 020501. https://doi.org/10.7189/jogh.09.020501
Lisa-Marie Thomas, Lucia D'Ambruoso, D. B. (2018). Verbal autopsy in health policy and systems: a literature review. *BMJ Global Health*, *3*(2). https://doi.org/10.1136/ bmjgh-2017-000639
Marsha, D. R., Sadruddin, S., Fikree, F. F., Krishnan, C., & Darmstadt, G. L. (2003). Validation of verbal autopsy to determine the cause of 137 neonatal deaths in Karachi, Pakistan. *Paediatric and Perinatal Epidemiology*, *17*(2), 132–142. https://doi.org/10.1046/j.1365-3016.2003.00475.x
Midhet, F. (2008). Validating the verbal autopsy questionnaire for maternal mortality in pakistan. *International Journal of Health Sciences*, *2*(1), 91–96. http://www.pubmedcentral.nih.gov/articlerender.fcgi?artid=3068724&tool=pmcentrez&rendertype=abstract
Moyer, C. A., Johnson, C., Kaselitz, E., & Aborigo, R. (2017). Using social autopsy to understand maternal, newborn, and child mortality in low-resource settings: a systematic review of the literature. *Global Health Action*, *10*(1), 1413917. https://doi.org/10.1080/16549716.2017.1413917
Mpimbaza, A., Filler, S., Katureebe, A., Quick, L., Chandramohan, D., & Staedke, S. G. (2015). Verbal autopsy: Evaluation of methods to certify causes of death in Uganda. *PLoS ONE*, *10*(6), e0128801. https://doi.org/10.1371/journal.pone.0128801
Murray, C. J. L., Lozano, R., Flaxman, A. D., Serina, P., Phillips, D., Stewart, A., James, S. L., Vahdatpour, A., Atkinson, C., Freeman, M. K., Ohno, S. L., Black, R., Ali, S. M., Baqui, A. H., Dandona, L., Dantzer, E., Darmstadt, G. L., Das, V., Dhingra, U., … Lopez, A. D. (2014). Using verbal autopsy to measure causes of death: The comparative performance of existing methods. *BMC Medicine*, *12*(1), 5. https://doi.org/10.1186/1741-7015-12-5
Nausheen, S., Soofi, S. B., Sadiq, K., Habib, A., Turab, A., Memon, Z., Khan, M. I., Suhag, Z., Bhatti, Z., Ahmed, I., Bahl, R., Bhutta, S., & Bhutta, Z. A. (2013). Validation of Verbal Autopsy Tool for Ascertaining the Causes of Stillbirth. *PLoS ONE*, *8*(10), e76933. https://doi.org/10.1371/journal.pone.0076933
Navale, S., Habumugisha, L., Amoroso, C., Sayinzoga, F., Gupta, N., & Hirschhorn, L. R. (2017). Exploring Drivers of Infant Deaths in Rural Rwanda Through Verbal Social Autopsy. *Annals of Global Health*, *83*(5–6), 756–766. https://doi.org/10.1016/j.aogh.2017.10.029
Nichols, E. K., Byass, P., Chandramohan, D., Clark, S. J., Flaxman, A. D., Jakob, R., Leitao, J., Maire, N., Rao, C., Riley, I., & Setel, P. W. (2018). The WHO 2016 verbal autopsy instrument: An international standard suitable for automated analysis by InterVA, InSilicoVA, and Tariff 2.0. *PLoS Medicine*, *15*(1), e1002486. https://doi.org/10.1371/journal.pmed.1002486
Nisar, Y. Bin, & Dibley, M. J. (2014). Determinants of neonatal mortality in Pakistan: Secondary

analysis of Pakistan Demographic and Health Survey 2006-07. *BMC Public Health*, *14*(1). https://doi.org/10.1186/1471-2458-14-663

Nonyane, B. A. S., Kazmi, N., Koffi, A. K., Begum, N., Ahmed, S., Baqui, A. H., & Kalter, H. D. (2016). Factors associated with delay in care-seeking for fatal neonatal illness in the Sylhet district of Bangladesh: Results from a verbal and social autopsy study. *Journal of Global Health*, *6*(1). https://doi.org/10.7189/jogh.06.010605

Rodriguez, L., Reyes, H., Tome, P., Ridaura, C., Flores, S., & Guiscafre, H. (1998). Validation of the Verbal Autopsy Method to Ascertain Acute Respiratory Infection as Cause of Death. *Indian Journal of Pediatrics*, *65*(4), 579–584. https://doi.org/10.1007/BF02730899

Schumacher R Diallo MO, Keita DR, Kalter HD, Pasha O:, S. E. (2002). *Mortality study in Guinae: Investigating the causes of death for children under 5 The Basic Support for Institutionalizing Child Survival Project (BASICS II). Arlington, VA; 2002.* http://www.basics.org/documents/pdf/guinea_mort.pdf

Serina, P., Riley, I., Hernandez, B., Flaxman, A. D., Praveen, D., Tallo, V., Joshi, R., Sanvictores, D., Stewart, A., Mooney, M. D., Murray, C. J. L., & Lopez, A. D. (2016). What is the optimal recall period for verbal autopsies? Validation study based on repeat interviews in three populations. *Population Health Metrics*, *14*(1). https://doi.org/10.1186/s12963-016-0105-1

Siddiqui, M. B., Ng, C. W., & Low, W. Y. (2016). Social Autopsy is a Dire Need for Investigating Child Mortality in Pakistan. *International Journal of Endorsing Health Science Research (Ijehsr)*, *4*(2), 01. https://doi.org/10.29052/ijehsr.v4.i2.2016.01-04

Siddiqui, M. B., Ng, C. W., & Low, W. Y. (2017). To Identify the Non-biological Causes of Child Mortalities in Developing Countries, Social Autopsy Tools should be Based on "The Pathway to Survival Conceptual Framework." *International Journal of Endorsing Health Science Research (Ijehsr)*, *5*(1), 01. https://doi.org/10.29052/ijehsr.v5.i1.2017.01-04

Siddiqui, M. B., Ng, C. W., Low, W. Y., Noushad, S., Ahmed, S., & Syed, S. (2014). Challenges and acceptance of the use of computer-assisted personal interviewtechnology for verbal autopsy/social autopsy child mortality survey in urban slums of Karachi, Pakistan. *International Journal of Endorsing Health Science Research (Ijehsr)*, *6*(2), 30. https://doi.org/10.29052/ijehsr.v6.i2.2018.30-42

Siddiqui, M. B., Ng, C. W., Low, W. Y., Syed, S., Ahmed, S., Noushad, S., Ali, A., Fatima, K., & Mirza, M. (2016). A Verbal/Social Autopsy (VASA) Child Mortality Inquiry to Investigate Under-Five Mortality Determinants in Slums of Karachi, Pakistan:A Mix Methods Interventional Study. *International Journal of Endorsing Health Science Research (Ijehsr)*, *4*(4), 01. https://doi.org/10.29052/ijehsr.v4.i4.2016.01-11

Siddiqui, M. B., Ng, C. W., & Yun Low, W. (2018). Methodologies of Verbal autopsy and Social autopsy tools, adopted by VASA studies in exploring under-five mortality determinants. *International Journal of Endorsing Health Science Research (Ijehsr)*, *6*(3), 43. https://doi.org/10.29052/ijehsr.v6.i3.2018.43-55

Soleman, N., Chandramohan, D., & Shibuya, K. (2006). Verbal autopsy: current practices and challenges. *Bull.World Health Organ*, *84*(0042-9686 (Print)), 239–245. http://www.scopus.com/inward/record.url?eid=2-s2.0-33644795635&partnerID=40&md5=09ffcd11e7668e66416b1919445bb9d2

Soofi, S. B., Ariff, S., Khan, U., Turab, A., Khan, G. N., Habib, A., Sadiq, K., Suhag, Z., Bhatti, Z., Ahmed, I., Bhal, R., & Bhutta, Z. A. (2015). Diagnostic accuracy of WHO verbal autopsy tool for ascertaining causes of neonatal deaths in the urban setting of Pakistan: A

hospital-based prospective study. *BMC Pediatrics*, *15*(1), 1–9. https://doi.org/10.1186/s12887-015-0450-4

Taherdoost, H. (2018). Validity and Reliability of the Research Instrument; How to Test the Validation of a Questionnaire/Survey in a Research. *SSRN Electronic Journal*, *5*(3), 28–36. https://doi.org/10.2139/ssrn.3205040

UN-Habitat. (2003). Slums of the World: the face of urban poverty in the new millennium. In *UN-Habitat, Nairobi*.

Waiswa, P. K., Kallander, S., Peterson, G., Tomson, G., & Pariyo. (2010). Using the three delays model to understand why newbornbabies die in eastern Uganda. *Tropical Medicine & International Health*, *8*(8), 964–972. https://doi.org/doi:10.1111/j.1365-3156.2010.02557.x

WHO/UNICEF. (2015). *WHO/UNICEF Joint Monitoring Programme for water supply and sanitation: Sanitation tables*. Who/Unicef. http://www.wssinfo.org/data-estimates/tables/

WHO, & Department of Measurement and Health Information Systems evidence and information for policy. (2004). *WHO Technical Consultation on Verbal Autopsy Tools: Review of the literature and currently-used verbal autopsy tools*. 1–45. http://www.who.int/healthinfo/statistics/mort_verbalautopsy.pdf

Zakar, M. Z., Zakar, R., Mustafa, M., Jalil, A., & Fischer, F. (2018). Underreporting of stillbirths in Pakistan: Perspectives of the parents, community and healthcare providers. *BMC Pregnancy and Childbirth*, *18*(1). https://doi.org/10.1186/s12884-018-1924-9

# Supporting information

**S1 Fig. Flow chart of the validation process**

**S2 VASA Questionnaire**

Flowchart on validation process

Click here to access/download;Figure;Validation details 300 dpi.tif

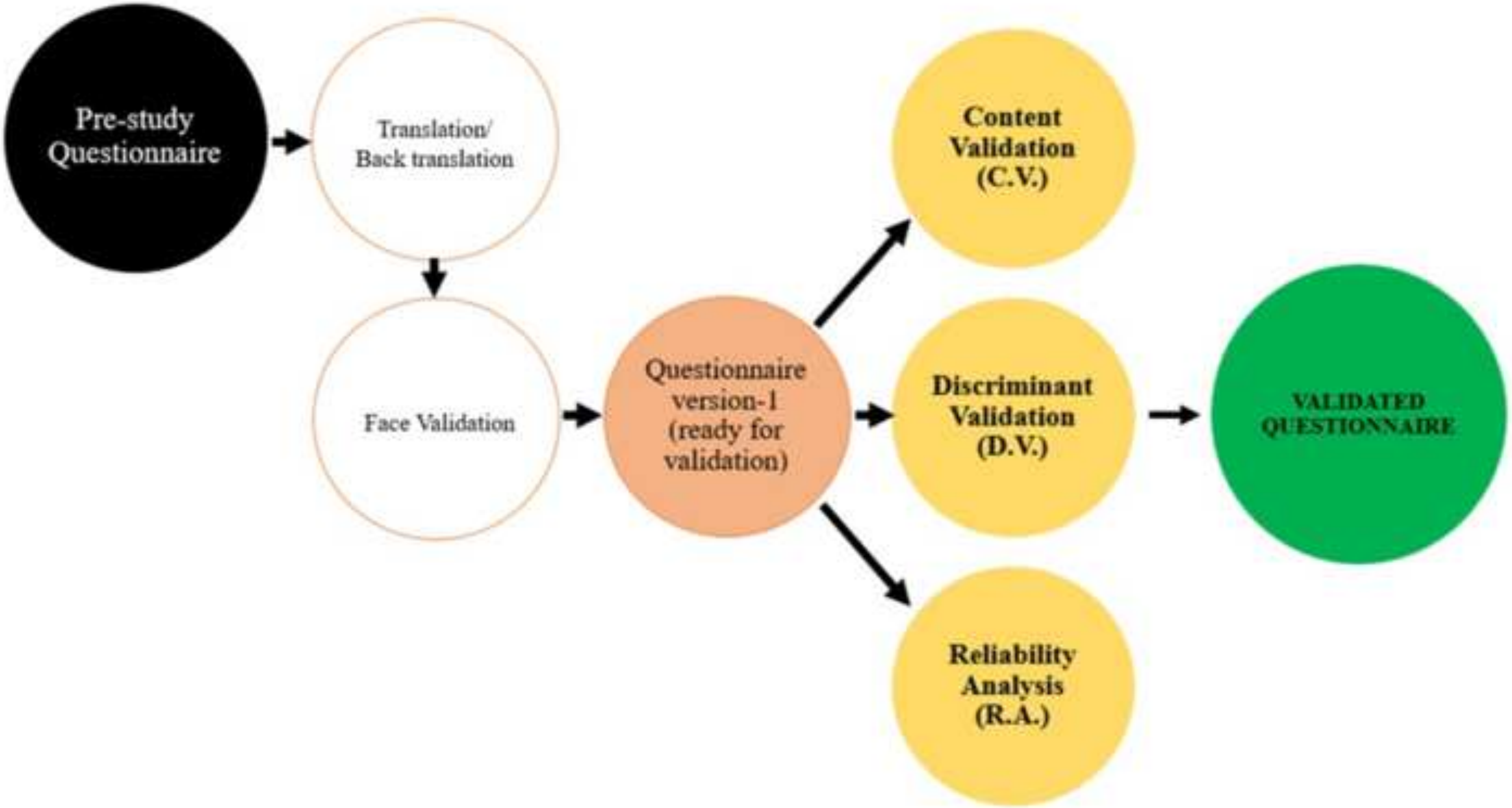

Figure 1

Click here to access/download;Figure;Validation details 300 dpi (1).tif

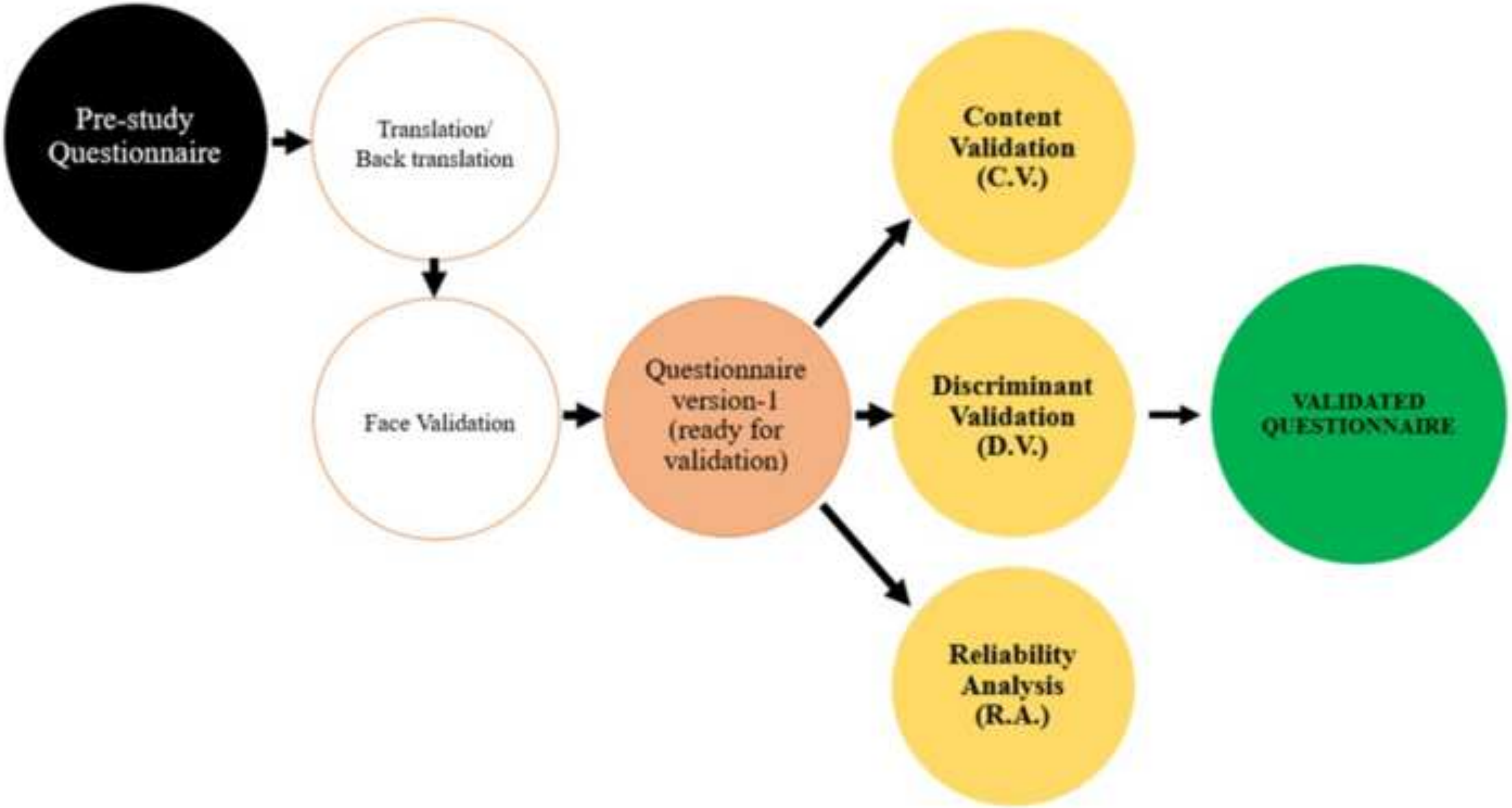

Click here to access/download
**Supporting Information**
VASA Questionnaire English.pdf

Click here to access/download
**Supporting Information**
VASA Translated Questionnaire.doc

Ethical clearance certificate-1

Click here to access/download
**Supporting Information**
UMMC.png

Ethical clearance certificate-2

Click here to access/download
**Supporting Information**
Picture2.png

# Validation of CHERG'S Verbal Autopsy-Social Autopsy (VASA) tool for ascertaining determinants and causes of under-five child mortality~~ies~~ in Pakistan.

**Muhammad Bilal Siddiqui[1,3*], Chiu Wan Ng[1], Wah Yun Low[2], Khadijah Abid[3]**

[1]Department of Social and Preventive Medicine, Faculty of Medicine, University of Malaya, Kuala Lumpur, Malaysia.
[2]Dean's Office, Faculty of Medicine, University of Malaya, Kuala Lumpur, Malaysia.
[3]Department of Public Health, Faculty of Life Sciences. Research Villa, Shaheed Zulfiqar Ali Bhutto Institute of Science and Technology (SZABIST), Clifton, Karachi, Pakistan.

***Corresponding Author:**
**Muhammad Bilal Siddiqui**
Email: muhammad.bilal@szabist.edu.pk (MBS)
drbilals@siswa.um.edu.my

**Formatted:** Font: (Default) Times New Roman

## Abstract

The majority (40%) of the world's under-five mortality burden is concentrated in nations like Nigeria (16.5%), India (16%), Pakistan (8%), and the Democratic Republic of the Congo (6%), where an undetermined number of under-five deaths go unrecorded.~~The major chunk (40%) global burden of under-five mortality is concentrated in places like Pakistan (8%), India (16%), Nigeria (16.5%), and the Democratic Republic of the Congo (6%), where an unknown proportion of deaths go unreported.~~ In low-resource settings throughout the world, the Verbal Autopsy-Social Autopsy (VASA) technique may assist assess under-five mortality estimates, assigning medical and social causes of death, and identifying relevant determinants.~~In low-resource settings and globally, the Verbal Autopsy-Social Autopsy (VASA) tool (involve data from a~~

well-structured questionnaire about the signs and symptoms and social circumstances of the deceased) can help estimate under-five mortalities; assign medical and social causes of deaths and determine the distribution of relevant determinants. Uncertainty regarding missing data in high-burden nations like Pakistan necessitates a valid and reliable VASA instrument.Uncertainty about missing data in high burden countries like Pakistan requires a validated and reliable VASA tool. This is the first study to validate CHERG's verbal autopsy/social autopsyVASA tool globally. In Pakistan, data from such a valid and reliable tool is vital for policy. This paper reports on the VASA tool's validation in Karachi, Pakistan. Validity and reliability of the CHERG VASA- tool were tested using face, content, discriminant validation, and reliability tests on one hundred randomly selected mothers who had recently experienced an under-five child a child death event. Data were computed on SPSS (version-21) and R software. Testing revealed high I-CVI (>81.43%); high Cronbach's Alpha (0.843); the accuracy of between 75-100% of the discriminants classifying births to live and stillbirths; and . The tool showed Item content Validity Index (I-CVI) (>82.07% and 88.98% respectively) with high accuracy (92% and 97% respectively) for assigning biological and social causes of child deaths, respectively. The CHERG VASA questionnaire is was found relevant to the conceptual framework and valid in Pakistan. This valid tool can assign accurate medical and non-medical causes of child mortality cases occurring in Pakistan.

# Introduction

Formatted: Font: Times New Roman

Verbal autopsy (VA) is a technique that involves structured questions focusing on the signs and symptoms the ~~children~~ deceased case faced during the illness but before the mortality incident. VA is used to record various relevant biological determinants (BDs) linked with child mortality incidents, and based on such information, VA helps in assigning~~s~~ the medical cause of death (MCoD). Similarly, social autopsy (SA), a relatively new technique with a questionnaire structure similar to VA, helps record non-biological determinants (especially the health-seeking process, and issues and barriers in the process of accessing health care during the child illness process but before the mortality event) so that the social cause of death (SCoD) of any child death can be concluded~~In a similar vein, Social autopsy (SA), a relatively new technique with a questionnaire structure similar to VA, but helps in recording non-biological determinants (especially health-seeking process, and issues and barriers in the process of accessing health care during the illness process but before the mortality event). Based on such information, SA helps in assigning social reasoning of any death (social cause of death-SCoD)~~. VA has been used in low-resource settings when the cause of any child death (CoD) has not been ascribed by a health care provider~~VA has been under practise in low resource settings where routinely the cause of death (CoD) are not assigned to these mortalities~~(Moyer et al., 2017; Nichols et al., 2018; Siddiqui, Ng, & Low, 2016). In contrast to VA, SA is not typically used for child mortality in the majority of developing countries, especially Pakistan.~~However, SA is not routinely practised in comparison to VA.~~ Both VA and SA not only provide the MCoD and SCoD to the child mortalities respectively but also assist in updating epidemiological estimates on the mortality~~ies~~

which have been missed to be recorded by the health system in these geographies(Gouda et al., 2017; Lisa-Marie Thomas, Lucia D'Ambruoso, 2018). Although VA has been used in nations with little resources, there is still a need to investigate a wider range of data on the societal factors that contribute to child mortality(Koffi, Mleme, et al., 2015; Siddiqui, Ng, & Low, 2016). Hence, ~~In~~in a more recent timeframe, a concept of running through ~~every~~ the missed mortality case with a VA and SA tool has been attempted in some countries (Cameroon(Koffi, Libite, et al., 2015),(Koffi, Mleme, et al., 2015), Mozambique(Källander et al., 2019), Niger(H. D. Kalter et al., 2015),(H. Kalter et al., 2016),(Koffi et al., 2016), Uganda(Waiswa et al., 2010),(Källander et al., 2011),(Hildenwall et al., 2008),(Källander et al., 2008), Rwanda(Navale et al., 2017),(Kagabo et al., 2018), Malawi(Koffi, Mleme, et al., 2015), Nigeria(Koffi et al., 2017), Guinea(Schumacher R Diallo MO, Keita DR, Kalter HD, Pasha O:, 2002)) which helped to get detailed evidence of social causes of different medically assigned CoDs. However, these attempts made use of different VA and SA tools in combinations[4,6–9],(Siddiqui et al., 2018).

The Child Health Epidemiology Reference Group (CHERG) was established in 2001 with the support of WHO and UNICEF to provide technical guidance to relevant institutions and organisations for collecting evidence on determinants and causes of child mortality and improving global child survival epidemiological estimates~~The Child Health Epidemiology Reference Group (CHERG) is a technical agency, established in 2001 through the support of WHO and UNICEF, in favour of providing technical guidance to the concerned relevant institutions and organizations for collecting evidence on determinants and causes of child mortality, and improving child survival epidemiological estimates at a global level~~(Koffi, Libite, et al., 2015),(Siddiqui et al., 2017). The agency developed an integrated VASA tool based ~~on a sound technical methodology~~on a most holistic approach ~~and~~ for recording different biological

Formatted

determinants (child-specific, maternal-specific, determinants across the CoC, and preventive measures taken before and during the pregnancy and child illness) and non-biological determinants (NBDs) identified through several models (The Three Delay Model), conceptual framework (TPtoSCF); and several recommendations for VA suggested by the World Health Organization (WHO & Department of Measurement and Health Information Systems evidence and information for policy, 2004) to record different biological and non-biological determinants affecting the under-five mortality~~the most holistic conceptual framework i.e., the "The Pathway to Survival Conceptual Framework" (TPtoSCF)~~(Siddiqui et al., 2018)~~,~~(CHERG, 2018a)~~. This framework has been considered so far as the most holistic framework for exploring the most relevant social determinants of mortality events~~(Koffi et al., 2019)~~,~~(H. D. Kalter et al., 2011). In addition to recording biological determinants, this tool also~~across the continuum of care ranging from the conception of mother to the death of the child due to illness. It~~ focuses on all the barriers which ~~ca~~come across as hindrances throughout the health-seeking process when the child was ill(Siddiqui et al., 2017). ~~This framework has been utilized in several countries[6,7,9,11] for exploring the most relevant social determinants of mortality events. CHERG's integrated VASA tool is one of the assets that~~ This tool, is one of the assets that can be utilized to acquire information on the relevant biological and non-biological determinants of child mortality for every missed child mortality case on a single visit to the household and has already supported several countries to gather evidence on the medical and social causes to child deaths(Bensaïd et al., 2016; H. Kalter et al., 2016; Siddiqui et al., 2018). The extensive data obtained from CHERG VASA tool based investigations provides highly elaborated evidence and information on extended determinants of child mortality which is of great public health importance(Bensaïd et al., 2016; Gouda et al., 2017; H. Kalter et al., 2016)~~.~~

**Formatted:** Font: Times New Roman, 12 pt

**Formatted:** Font: Times New Roman

Similar to any data collection tool, it's always been a concern ~~of~~ whether the data collected through VA and SA are correct, reliable, and represent~~s~~ the information that the researcher intends to collect. The process of validation involves testing the questionnaire on validity and reliability parameters. To obtain the best possible data, many efforts have been undertaken globally in refining the methodology of VA(Aggarwal et al., 2013; Marsha et al., 2003; Midhet, 2008; Mpimbaza et al., 2015; Murray et al., 2014; Nausheen et al., 2013; Soofi et al., 2015). In continuation of such efforts, a few ~~of the~~ validation studies have been undertaken globally as well in Pakistan~~, (a country with nearly 8% of the global under five mortality burden~~(IGME, 2020)~~)~~ to assess the capacity of VA tools in providing precise and reliable evidence on the causes of child mortality(Aggarwal et al., 2013; Marsha et al., 2003; Nausheen et al., 2013; Soofi et al., 2015). However, no attempts have been made to validate CHERG's integrated VASA tool. Considering the current status of child mortality estimates in Pakistan~~Keeping in view of the current status of child mortality estimates in Pakistan~~(Afzal & Yusuf, 2013; IGME, 2020; Nisar & Dibley, 2014), CHERG's integrated VASA tool was validated to gather evidence on a comprehensive list of medical and social determinants and medical and social causes of under-five mortality in Pakistan, where child mortality cases may be missed to be registered under national CRVS and Health Management Information System.~~it has been aimed to undertake a validation study of CHERG's integrated VASA tool to gather evidence on a comprehensive list of determinants: and medical and social causes of under-five mortality, in Pakistan, where there is a high chance that the child mortality cases might be missed to be registered under national CRVS and Health Management Information System.~~ This is the first time a VASA integrated tool has been validated so thoroughly, concentrating on most of the approaches used to evaluate

validity and reliability. We expect the validated tool would provide comprehensive data on under-five mortality in Pakistan.~~This validation study is unique in that it is the first time that a VASA integrated tool has been validated in such depth, focusing on the majority of the techniques used to assess the validity and reliability of the tool. We hope that the validated tool will be highly beneficial in providing extended and detailed data on the most relevant determinants and causes of under-five mortality in Pakistan.~~ T~~ In t~~his paper~~, we share~~ presents the ~~validation~~ results of the validation ~~the~~ of the CHERG VASA tool. The validated instrument will be used to conduct a future VASA child mortality survey in Pakistan's underprivileged community.~~This validated tool will be utilized to undertake a future VASA child mortality survey in the underserved population of Pakistan.~~

# Materials and Methods

**Formatted:** Font: Times New Roman

This validation study is a component of the *Karachi~~'s~~ VASA child mortality study*(Siddiqui, Ng, Low, et al., 2016) and is designed to objectively assess the validity and reliability estimates of the CHERG's VASA questionnaire, which was later utilized in *Karachi~~'s~~ VASA child mortality study*.

## The sample size

**Formatted:** Font: Times New Roman

~~Despite the fact that~~Although there are no specific guidelines for the sample size of respondents required for pre-survey validation of the VASA tool(Anthoine et al., 2014), the only available guideline suggests(Hertzog, 2008) that, in general, the sample size required for any questionnaire validation should be a minimum of 20% of the total sample size of the study. Since 400 under-5

**Formatted:** Space After: 0 pt, Line spacing: Double

died cases were included in the Karachi VASA child mortality study, 20% of 400 cases (80 cases) were selected from the study sites to participate in the questionnaire validation study. The original sample size of 80 was increased to 100 to overcome the issue of potential data entry errors.A total of 400 under-five deceased cases were included in the VASA child mortality study in Karachi; therefore, 20 percent of 400 cases, is 80 cases (in addition to the 400 cases from the Karachi VASA child mortality study) were recruited from the study sites to participate in the questionnaire validation study. The sample size of 80 cases was later inflated to 100 cases to overcome the issue of non-respondents. Mothers of these 100 under-five deceased cases (n=100) were selected at random from 12 selected sites i.e., Cheneser goth (CG), Mujahid colony (MC), Haryana Colony (HC), Sultanabad (SD), Bilal colony (BC), Sherabad (SA), Yaseenabad (YS), Landhi slums (LS), Khuda ki Basti (KKB), Safora goth (SG), Malir slum (MS), and Sultanabad (SD), based on the number of deaths occurred in these sites during the past one year (from June 1, 2015, to May 3, 2016) before the data collection. These 12 study sites were located in five of the city's districts, into which the city was subdivided at the time of the study, namely, District Malir, District Central, District South, District West, District East, and District Korangi. Two study sites (one urban and another one urban-slum) were randomly selected from each of the six districts, making a total of 12 study sites for research purposes. The urban sites and the urban-slum sites were classified based on the definition of a slum from UN-Habitat(UN-Habitat, 2003). To ensure that the sample was representative of the entire population, the sample (100 mothers of deceased under-five deaths, falling under strict inclusion and exclusion criteria) was drawn from all six districts of Karachi.

These 12 locations throughout the city of Karachi were chosen based on criteria to identify geographical points throughout the entire city where residents belonging to the most common

**Formatted:** Font: Times New Roman, 12 pt

**Formatted:** Font: Times New Roman, 12 pt

**Formatted:** Font: Times New Roman, 12 pt

**Formatted:** Font: Times New Roman, 12 pt

**Formatted:** Font: Times New Roman, 12 pt

**Formatted:** Font: Times New Roman, 12 pt

**Formatted:** Font: Times New Roman, 12 pt

**Formatted:** Font: (Default) Times New Roman, 12 pt

and frequently found ethnic backgrounds (Punjabi, Sindhi, Balochi, Siraiki, Pathan, Urdu speaking, Kashmiri, Hindko, and Brahvi) live within at least 2 kilometres of residential locations. This criterion, ensured that the sample is representative of the general population. These twelve study sites include Cheneser goth (CG), Mujahid colony (MC), Hazara colony (HzC), Sultanabad (SD), Bilal colony (BC), Shireen Jinnah colony (SJC), Yaseenabad (YS), Landhi slums (LS), Khuda ki Basti (KKB), Safora goth (SG), Malir slum (MS), and Sultanabad (SD).

## Sampling methodology

The estimates of all the under-five deaths (occurred between 01st June 2015 till 03rd May 2016) from each of the study sites were identified from each site through multiple sources (household visits i.e., snowball sampling; local graveyards; local town health and union council offices). To fulfil the sample size of 100 respondents for the validation study, each identified area/ study site was allocated a quota based on the **proportionate quota sampling technique** (proportionally of the overall sample size required for the study). The details (quota calculation) are available in Table 1.

**Table 1: SAMPLING QUOTA CALCULATION**

| S. No | STUDY SITE | SITE CODE | TOTAL IDENTIFIED DEATHS IN EACH SITE (till the end of june 2016) (A) | (DEATH IN EACH SITE/TOTAL DEATHS IN 12 SLUMS) X (SAMPLE SIZE) (B) | QUOTA TO BE ALLOCATED FOR EACH SITE FOR VALIDATION (C) |
|---|---|---|---|---|---|
| 1 | Cheneser goth | CG | 210 | 210/1926*100 | 11 |
| 2 | Mujahid colony | MC | 168 | 168/1926*100 | 9 |
| 3 | Hijrat colony | HC | 159 | 159/1926*100 | 8 |
| 4 | Haryana colonyHazara colony | HZC | 57 | 57/1926*100 | 3 |
| 5 | Sultanabad | ST | 111 | 111/1926*100 | 6 |
| 6 | Bilal colony | BC | 189 | 189/1926*100 | 10 |
| 7 | SherabadShireen Jinnah Colony | SJCSA | 243 | 243/1926*100 | 13 |
| 8 | Yaseenabad | YS | 183 | 183/1926*100 | 9 |
| 9 | Landhi slum | LS | 150 | 150/1926*100 | 8 |
| 10 | Khuda ki Basti | KKB | 126 | 126/1926*100 | 6 |



**Formatted:** Font: Times New Roman

**Formatted:** No underline

**Formatted:** Font: No underline, Font color: Black

**Formatted:** Font: Times New Roman

| | | | | | |
|---|---|---|---|---|---|
| 11 | Safora goth | SG | 183 | 183/1926*100 | 9 |
| 12 | Malir slum | MS | 147 | 147/1926*100 | 8 |
| | Total | | 1926 | | 100 |

## Inclusion and exclusion criteria

**Formatted:** Font: Times New Roman

To reduce response bias, only women whose children under the age of five passed away during the previous year (from 01st June 2015 till 3rd May 2016) were counted in this validation study.Only those mothers were included in this validation process, whose under five children died within the past six weeks (to minimize response bias) due to any illness and those who have death certificates (issued by health authority or health care practitioner/s) with the cause of death (CoD) mentioned on the death certificate.

**Formatted:** Font: Times New Roman, 12 pt

## Ethical clearance for Karachi's VASA child mortality study

**Formatted:** Font: Times New Roman

The Medical Ethics Committee, University Malaya Medical Center (UMMC), located in Kuala Lumpur, Malaysia; and Advance Educational Institute and Research Centre (AEIRC), located in Karachi Pakistan granted the ethical clearance (with ethical clearance document no: 201412-847 and ethical clearance document no: MU/ECA/48/776 respectively) for the Karachi VASA child mortality study, for which this validated questionnaire was usedThe ethical approval of the Karachi's VASA child mortality study for which this validated questionnaire will be utilized was gained from the Medical Ethics Committee, University Malaya Medical Center (Ethical clearance document no: 201412-847) located in Kuala Lumpur, Malaysia; and Advance Educational Institute and Research Centre, (Ethical clearance document no: MU/ECA/48/776) located in Karachi Pakistan.

After being informed of the study's purpose and procedures, all participants were asked if they would be willing to participate~~,~~ and informed written consent was obtained. Those who did not wish to participate in the study were given the option to withdraw themselves from the study, and this was done with respect. All responses were treated as strictly confidential. It was ensured that, i~~n the event that~~f a respondent experienced any emotional disturbance during the data collection process, the respondent would be referred to a health professional; however, no such case was ~~discovered~~encountered.~~ Because the study had no negative impact on the subjects' rights and well-being, there was no need for financial compensation or other financial assistance.~~

## Structure of the CHERG's VASA Questionnaire

**Formatted:** Font: Times New Roman

The CHERG's VASA questionnaire(CHERG, 2018b) is a quantitative tool for collecting data on the signs and symptoms of illness (including varied BDs) and the relevant history of social circumstances (NBDs) ~~events~~ right from the conception of the mother until the death of the ill child including few open-ended questions i.e., the open narratives (which specifically asks for any additional notes to be mentioned by the respondents in relation to the Verbal autopsy component as well as the summary from the interviewer of the whole case). It also quantitatively captures the signs and symptoms a child encountered during the illness and the social history of the deceased child before the death of the child. The initial pre-validated CHERG VASA questionnaire consisted of VA and SA components having 14 different modules (sections) focusing on the different variables ranging from the demographic profile of the respondent, family of the deceased child, parents of the deceased child, healthcare-seeking process and socioeconomic status to variables encompassing the entire continuum of care during the pregnancy till the death of the child.

After the section on the VASA general information (which collects information on the general variables), the VA component has 06 modules altogether, while the SA component has 07 modules (table 2~~1~~, columns 1&2). These VA and SA components and relevant modules have been arranged in such an order that as the interview proceeds with the respondent, the sequence of events gets unfolds (table 2~~1~~, column 3). To record relevant information ~~of~~ on relevant age groups (i.e., neonatal deaths, and post-neonatal deaths), specific skip patterns exist in the tool.

**Table 2: Structure of CHERG's VASA questionnaire**

| **Details of different modules of VA & SA components included in the VASA tool** | | | **Sequence of different modules of VA & SA components in VASA tool** |
|---|---|---|---|
| **VASA General Information:**<br>• Deceased background,<br>• Interview details,<br>• Consent,<br>• Respondent's details,<br>• Details of others present during the interview | **VA Sections:**<br>VA-1: Background<br>VA-2: Maternal History<br>VA-3: Neonatal deaths<br>VA-4: Infants and child deaths<br>VA-5: Health records<br>VA-6 Open-ended questions (any additional information about illness and death) | **SA Sections:**<br>SA-1: Mother and her household<br>SA-2: Social capital<br>SA-3: Maternal history<br>SA-4: Care-seeking for maternal complications<br>SA-5a: Care of the newborn<br>SA-5b: Preventive care of post-neonates<br>SA-6: Care-seeking of child's fatal illness<br>SA-7: Open-ended questions (any additional information about social circumstances) | (VA-1); (SA-3); (VA-2); (SA-4); (SA-5a); (VA-3); (SA-5b); (VA-4); (SA-6); (VA-5); (SA-1); (SA-2); (VA-6) & (SA-7). |

## Translation & Back Translation

Formatted: Font: Times New Roman

The CHERG VASA tool is freely available(CHERG, 2018b). The tool is available either in the integrated version of VASA ~~or~~ or in different sections separately. Before validation, the questionnaire was forward translated into the local language, Urdu, and then back-translated to English based on the **"Brislin Back-translation"** technique(Bennet & Ritchie, 1975). The process involved a professional language translator and a panel of professional bilingual subject

experts (paediatricians, neonatologists, gynaecologists, obstetricians, social scientists, and psychologists). The final translated version was processed for the validation process.

## Data collection for the validation study

Formatted: Font: Times New Roman

~~A total of 100 interviews were conducted.~~ The respondents (mothers of deceased children) were recruited, and their written consents ~~were~~ were obtained. A total of 100 VASA interviews were conducted. Data was recorded on both the platforms i.e., Paper-And-Pencil Interviews (PAPI)(i.e., ~~paper~~ paper-based VASA tool); and Computer Assisted Personal Interview software (CAPI)(an electronic platform format for data collection with skip patterns using CSPro software application) and ~~. The data collected on both the platforms~~ were ~~initially~~ compared to check any inconsistencies. ~~, h~~However, no inconsistencies were encountered between the PAPI and CAPI platforms. The completion rate of the questions on both PAPI and CAPI was the same (100%), however, there were some issues by the interviewee and interviewer on the implementation of these two methods which are elaborated in a qualitative study(Siddiqui et al., 2014) The only difference between the two platforms was the ease of data collection and less time utilized for data collection by the data collectors(Siddiqui et al., 2014). Data quality was checked by a quality management team and researcher. None of the mothers refused or rejected the interview. Extensive training to the data collectors and data entry operators (with a minimum educational background of having Intermediate level (Higher Secondary School Certificate i.e., Grade-12) was given on communication skills, details of the project, questionnaire design and data entry process. Collected data were shared with a panel of subject specialists (five Paediatricians with expertise in Neonatology~~a team of five paediatricians, five neonatologists, five gynaecologists, five obstetricians, and five social scientists~~) trained for PCVA for reviewing the data and

Formatted: Font: Times New Roman, 12 pt

assigning the MCoD and SCoD (five sociologists) through VA and SA components of the integrated VASA tool. Medical CoD was assigned by the physicians (five Paediatricians with expertise in Neonatologyfive paediatricians and five neonatologists) (through Physician Certified Verbal Autopsy-PCVA), where the PCVA trained physicians reviewed the mortality case reports (complete VASA data) including signs and symptoms, open narratives, medical history and sequence of events prior to death -of deceased children from the VA component of the VASA integrated tool and assigned the most probable MCoD. Initially the data was shared with one reviewer, who assigned a MCoD. Once received, the data was then shared with second reviewer and the level of agreement was checked. In case of any disagreement between the initial two reviewers, a third reviewer was assigned until the agreement between the two different reviewers was achieved. The MCoD assigned by the reviewers were kept confidential by the other reviewers. While conducting PCVA, reviewers were kept blinded with the MCoD assigned on the death certificates of those cases (under-five mortalities) with a record of death certificates. Similarly, the SCoD were assigned by the social scientists through the same process after reviewing the SA component of the integrated VASA tool. The analysis was computed on SPSS (version 21) and R software.

**Formatted:** Font: Times New Roman, 12 pt

**Formatted:** Font: Times New Roman, 12 pt

## Tool's validation procedure

**Formatted:** Font: Times New Roman

As illustrated in Table 32 and Fig 1, the CHERG VASA tool was evaluated on its ***validity*** and ***reliability*** through face validation, content validation, discriminant validation, and reliability analysis and the details are described in the respective sections below.

| Table 3: Summary of methodology for validation of CHERG's VASA questionnaire | | |
|---|---|---|
| **Assessment** | **Method** | **Outcome** |
| **Face Validation** | **Subjective review** of the tool by a panel of experts | The updated questionnaire which was assessed on C.V., D.V., & R.A. (below in this table) |
| **Content Validation (C.V.)** | Calculation of **I-CVI** using agreement level of 05 experts on a dichotomous scale | I-CVI ranged between 81.43% to 96.36%. |
| **Discriminant Validation (D.V.)** | Calculation using **Eigenvalue (E.V.)**, **Canonical correlation (C.C.)**, **Wilk's Lambda (W.L.),** and **Accuracy** | ● E.V.=between 0.61 to 735.11<br>● C.C.= between 0.86 and 1.00<br>● W.L.=<0.001<br>● Accuracy of >75.07% |
| **Reliability Analysis (R.A.)** | Calculation using **Cronbach's Alpha (C.A.) statistics** | C.A ranged between 0.843 and 0.973 |
| **C.V.:** Content validation; **D.V.:** Discriminant Validation; **R.A.:** Reliability Analysis; **I-CVI:** Item-content Validity Index; **E.V.:** Eigenvalue; **C.C.:** Canonical correlation; **W.L.:** Wilk's Lambda; **C.A.:** Cronbach's Alpha; | | |

**Figure 1: Flow chart of the validation process**

# Results

The sample of 100 responding mothers had the following demographic characteristics (table 4-).

| Table 4: Demographic characteristics of the enrolled households for validation study | | |
|---|---|---|
| **Characteristics** | **Frequency (n)** | **Percent%** |
| **Mothers' characteristics** | | |
| | 100 | 100% |
| Married or living with a man | 979/100 | 979% |
| Widowed | 31/100 | 31% |
| | **Mother's mean age when first married (years):** | |
| 15-20 | 82/100 | 2%8 |
| 21-24 | 5431/100 | 31%54 |
| 25-28 | 41/10023 | 41%23 |
| 29-32 | 22/10011 | 22%11 |
| 33-3633-36 | 4/1004 | 4% |
| | **Mother years of schooling (mean years of schooling):** | |
| Didn't attend school | 8/10012 | 8%12 |
| Primary (1-5 years) | 45/10040 | 45.0%40 |
| Middle (6-8 years) | 25/10017 | 25%17 |
| Secondary (9-10 years) | 19/10026 | 219.0%6 |
| Intermediate (11-12 years) | 3/1005 | 3%5 |
| >12 years | 0/100 | 0% |
| **Main breadwinner** | | |
| Father | 4638/100 | 4638% |
| Mother | 42/10031 | 42%31 |
| Both | 20/10023 | 20%23 |
| **Main breadwinner's occupation** | | |
| Farmer/agricultural works | 0/100 | 0% |
| Poultry or cattle raising | 0/100 | 0% |
| Domestic servant | 4334/100 | 34%43 |
| Home-based manufacturing | 22/10029 | 22%29 |
| Unskilled labourer | 17/10010 | 17%10 |
| Semi-skilled labourer/service provider | 15/1009 | 15%10 |
| Factory worker, blue-collar service | 10/1008 | 10%8 |
| Business owner (shop) | 2/1001 | 2%1 |
| Professional/technician | 0/100 | 0% |

**Formatted:** Font: Times New Roman

**Formatted:** Left, Tab stops: 1.13", Centered + 1.45", Left

| | | |
|---|---|---|
| **Wealth quintiles of the households*** | | |
| Lowest | 33/100 | 33% |
| Second | 39/100 | 39% |
| Middle | 21/100 | 21% |
| Third | 5/100 | 5% |
| Fourth | 2/100 | 2% |
| **Type of house (bricked/open-air etc.)** | | |
| Open air/Tents/bush houses | 5/100 | 5% |
| Asbestos sheet roofed with bricked boundary | 39/100 | 39% |
| Bricked roofed with bricked boundary | 56/100 | 56% |
| **Access to toilets** | | |
| No facility/bush /field | 2/100 | 2% |
| Unimproved facility* | 4/100 | 4% |
| Shared facility | 6/100 | 6% |
| Improved facility* | 88/100 | 88% |
| **Access to electricity** | | |
| Yes | 95/100 | 95% |
| No | 5/100 | 5% |
| **Household size** | | |
| **<5** | 24/100 | 24.0% |
| **≥5 <10** | 66/100 | 66.0% |
| **≥10<15** | 10/100 | 10.0% |
| **Use of energy for cooking** | | |
| Gas | 35/100 | 35% |
| Gas and Kerosine Oil | 43/100 | 43% |
| Kerosine Oil | 20/100 | 20% |
| Wood | 3/100 | 3% |
| **Separate place for cooking** | | |
| Yes | 85/100 | 85% |
| No | 15/100 | 15% |
| **Source of drinking water under the utilization by the households** | | |
| Pond water | 5/100 | 5% |
| Tap water | 48/100 | 48% |
| Treated water | 47/100 | 47% |
| **Access to boiled drinking water** | | |
| Yes | 7/100 | 7.0 |
| No | 93/100 | 93.0 |
| **Birth registration of the existing <5 children** | | |
| Yes | 3/100 | 3% |
| No | 388/100 | 97% |
| *Improved sanitation facilities are likely to ensure hygienic separation of human excreta from human contact. They include flush/pour flush (to the piped sewer system, septic tank, pit latrine), ventilated improved pit (VIP) latrine, pit latrine with slab, and composting toilet(WHO/UNICEF, 2015) | | |

## Characteristics of included deceased children

The table 5, mentions the details of some of the important characteristics of the deceased children included in the validation study.

| Table 5: Characteristics of the enrolled deceased under-five children for the validation study | | |
|---|---|---|
| | **N** | **%** |
| **Gender of the deceased children** | | |
| **Boy** | **59** | **59** |
| **Girl** | **41** | **41** |
| **Age of deceased children** | | |
| **Stillbirths** | **33** | **33** |
| **Livebirths** | **67** | **67** |

**Formatted:** Font: (Default) Times New Roman, 8 pt
**Formatted:** Justified, Indent: First line: 0"
**Formatted:** Font: (Default) Times New Roman, 8 pt
**Formatted:** Font: 12 pt, Not Bold
**Formatted:** Font: 12 pt, Not Bold
**Formatted:** Font: Bold
**Formatted Table**
**Formatted:** Font: Bold
**Formatted:** Font: Bold
**Formatted:** Font: Bold
**Formatted:** Font: Bold
**Formatted:** Left

| | | |
|---|---|---|
| Place of birth of the deceased children | | |
| Government hospital | 17 | 17 |
| Private hospital | 41 | 41 |
| Home | 42 | 42 |
| | | |
| Age at which child died | | |
| Born dead | 67 | 67 |
| Neonate | 17 | 17 |
| 1 month-59 month | 16 | 16 |
| Frequency of different medical causes of under-five deaths | | |
| Preterm delivery (P07) | 7 | 7 |
| Birth asphyxia (P21) | 4 | 4 |
| Neonatal Pneumonia (P23) | 2 | 2 |
| Neonatal Tetanus (A33) | 2 | 2 |
| Neonatal Sepsis (P63) | 1 | 1 |
| Neonatal Meningitis (G03) | 1 | 1 |
| Neonatal Congenital malformations (Q89) | 0 | 0 |
| Neonatal Sudden unexplained death (R99) | 0 | 0 |
| Neonatal Diarrhoea (A09) | 0 | 0 |
| Postneonatal Diarrhoea (A09) | 5 | 5 |
| Postneonatal Pneumonia (J22/J18) | 4 | 4 |
| Postneonatal Severe Malnutrition (D64) | 3 | 3 |
| Postneonatal Hemorrhagic fever (A99) | 2 | 2 |
| Postneonatal Meningitis (E46) | 1 | 1 |
| Postneonatal Malaria (B54) | 1 | 1 |
| Mother's hospitalization | | |
| Yes | | |
| No | | |
| Place of birth | | |
| Government hospitals | 46 | 46 |
| Private hospitals | 37 | 37 |
| Home | 17 | 17 |
| Antenatal history | | |
| Attended at least antenatal visit in HCF | | |
| 1 visit | 6 | 6 |
| 2 visits | 24 | 24 |
| 3 visits | 14 | 14 |
| 4 visits | 16 | 16 |
| No visit | 40 | 40 |
| Child's hospitalization | | |
| Yes | 11 | 11 |
| No | 89 | 89 |
| Child's hospital records | | |
| Yes | 31 | 31 |
| No | 69 | 69 |
| Which health care provider was consulted by mother during ANC | | |
| Did not see any HCP for ANC | 41 | 41 |
| Local dai | 46 | 46 |
| Private clinic | 13 | 13 |
| HCP: Healthcare professional, ANC: Antenatal care | | |

## (a) Face validity assessment

~~Here~~For assessing the face validity of the tool, the questionnaire was reviewed and critically evaluated by a ~~the same~~ panel of 6 experts—a researcher and 5 expert(s) in the relevant field of interest

(e.g., paediatrician, gynaecologist, obstetrician, social scientist, and psychologist(Lebet, Asaro, Zuppa, & Curley, 2018; Trockel et al., 2018) (utilized during the translation exercise) on the tool's format to find out, whether all the sections and items be easily readable, clear, and straightforward to understand and comprehend by the interviewees and respondents; and on the need of rephrasing, omitting, or adding any questions. The item was phrased in such a way that the respondent would need to think before responding, thereby minimizing the chances of response bias. The panellists found no need for any modifications in terms of rephrasing of questions, omitting any option of any item, adding an option of any item, etc. The team identified that the items of the tool were phrased in such a way that the respondent would need to think before responding, thereby minimizing the chances of response bias. The translated questionnaire which went for face validation was then named **"VASA Questionnaire-version-1".** This version of the questionnaire was utilized for the content validation as well as to collect data for undertaking discriminant validation.

Formatted: Font: Times New Roman

## (b) Content Validity assessment

Formatted: Font: Times New Roman

It involved a panel of five subject specialists (paediatrician, gynaecologists, obstetrician, social scientist, and psychologist) who gave their ratings on different parameters (especially on the comprehensiveness of the tool and the relativity of the items on the tool with the background conceptual framework of the topic), five subjective reviews, each with five homogenous experts from each professional expert (five paediatricians, five neonatologists, five gynaecologists, five obstetricians, five social scientists, and five psychologists). Each member from a panel was requested to rate every item on the VASA Questionnaire-version-1 on a 5-point Likert scale based on the relevancy to the conceptual framework (1=not relevant, 2=somewhat relevant,

Formatted: Font: Times New Roman, 12 pt

Formatted: Font: Times New Roman

3=relevant, 4=quite relevant, 5=highly relevant). The ratings were calculated using **Item-content Validity Index (I-CVI)**. For each item, the I-CVI was computed as the number of experts who rated an item on the top two boxes (i.e., quiet relevant or highly relevant), divided by the total number of experts. The level of agreement between experts should be as high as possible to make the I-CVI acceptable. The data on content validation is presented in Table 65 under different themes/constructs. These themes/constructs were clustered either as pre-birth themes/constructs and or post-birth themes/constructs, sections 1 through 8 cover pre-birth constructs, and sections 9 through 16 cover during and post-birth constructs. The average rating of each section was more than 4 (on a scale of 1 to 5), and the I-CVI is more than 80% for all the sections of the VASA questionnaire. Relatively lower I-CVI was observed for *"symptoms before pregnancy"* (81.43%) and *"Birth History of Child"* (81.82%) sections.

**Table 65: Average expert rating (from five homologous panels of five different professional expert groups) and overall Item-content Validity Index (I-CVI) by sections of CHERG's VASA questionnaire**

| VASA questionnaire themes | Content Validity (C.V.) (average rating) | | | | | Average I-CV (%)I |
|---|---|---|---|---|---|---|
| | Rater 1 (n=5) | Rater 2 (n=5) | Rater 3 (n=5) | Rater 4 (n=5) | Rater 5 (n=5) | |
| **Content validation (C.V.) of pre-birth themes/constructs* of VASA questionnaire** | | | | | | |
| Demographic and social constructs | 4.56 | 4.44 | 4.47 | 4.53 | 4.53 | 92.94% |
| Symptoms before pregnancy | 4.43 | 4.21 | 4.14 | 4.21 | 4.29 | 81.43% |
| Symptoms during initial two trimesters | 4.45 | 4.24 | 4.27 | 4.24 | 4.36 | 82.42% |
| Symptoms during last trimesters | 4.38 | 4.38 | 4.38 | 4.38 | 4.38 | 87.50% |
| Care Seeking behaviour for symptoms of last trimester | 4.64 | 4.45 | 4.50 | 4.45 | 4.50 | 96.36% |
| Symptoms during labor and care-seeking behaviour | 4.50 | 4.30 | 4.40 | 4.40 | 4.50 | 88.00% |
| Treatment of labor symptoms and post-delivery | 4.34 | 4.16 | 4.19 | 4.22 | 4.28 | 83.75% |
| Knowledge Attitude and Behavior on maternal symptoms | 4.53 | 4.39 | 4.47 | 4.43 | 4.49 | 88.98% |
| **Content validation (C.V.) of during & post-birth themes/constructs* of VASA questionnaire** | | | | | | |
| Birth History of Child | 4.41 | 4.27 | 4.27 | 4.23 | 4.36 | 81.82% |
| Physical Malformations Of Child's Body | 4.57 | 4.43 | 4.36 | 4.43 | 4.50 | 88.57% |
| Child Illness Details | 4.31 | 4.17 | 4.14 | 4.17 | 4.21 | 82.07% |
| Health Seeking Behavior Of Child Illness | 4.63 | 4.50 | 4.56 | 4.56 | 4.56 | 95.00% |
| Health care professionals (HCP) involved during Child Illness | 4.46 | 4.21 | 4.36 | 4.32 | 4.36 | 86.43% |
| Services Cost Burden and Delays | 4.50 | 4.21 | 4.29 | 4.29 | 4.43 | 91.43% |
| Preventive Measures Between Child Birth and Illness | 4.68 | 4.55 | 4.64 | 4.55 | 4.68 | 92.73% |
| Community Efforts during child illness | 4.42 | 4.21 | 4.26 | 4.26 | 4.32 | 85.26% |
| Cause of death-Medical | 4.31 | 4.17 | 4.14 | 4.17 | 4.21 | 82.07% |
| Cause of death-Social | 4.53 | 4.39 | 4.47 | 4.43 | 4.49 | 88.98% |

*Based on a thorough review of the questionnaire, the content of the VASA questionnaire can be divided into two clusters, sections 1 through 8 covers pre-birth constructs and sections 9 through 16 covers during and post-birth constructs. **C.V.**: Content validation; **I-CVI:** Item-content Validity Index;

## (c) Discriminant validity (construct validity) assessment

**Formatted:** Font: Times New Roman

It shows the distinctiveness of different constructs within the questionnaire and helps to identify whether the items on the questionnaire that are not supposed to be related to each other are ~~actually~~ unrelated or not. It is one of the types of construct validity. The **"VASA Questionnaire-version-1"** (after the face validation exercise) of the tool was utilized for data collection from the respondents who fall within the inclusion and exclusion criteria. All the mothers ~~of the~~ identified with ~~the~~ a recent history of child mortality, and none of the mothers refused or rejected the interview. Collected data were shared with verbal autopsy reviewers (physicians). The reviewers used their clinical reviewing expertise (a process called *"Physicians certified VA technique-PCVA"*) in assigning the medical cause of death~~s~~. The complete data (including the CoD assigned by the physicians) were computed on SPSS (version 21) and R for the validation process. For pre-birth constructs, *"birth status of the child"* and for during and post-birth constructs *"diseases or conditions directly leading to death"* are the decisive elements with potential discriminating power. Clear clusters and grouping in response patterns on VASA elements were found for the pre-birth constructs. Therefore, valid response differences between the live birth group and stillbirth group (on the elements of each section) were used for the discriminant validity of the pre-birth constructs of the tool. Similarly, valid response differences among different diseases or conditions directly leading to death were used for the discriminant validity of during or post-birth constructs of the tool.

For pre-birth constructs of the questionnaire, discriminant analysis was performed using birth status as a dependent variable, which was defined in such a way that birth status was coded as

“1” (one) in case of stillbirth and “0” (zero) in case of live birth, and elements of each section as discriminating or classification variables. The **goodness of fit summary of discriminant analysis models**, such as eigenvalue (E.V.) of canonical discriminant function, canonical correlation (C.C.), and Wilks’ Lambda (W.L.) statistics was used for the discriminant validity measures of the constructs. To analyze the discriminant validity of post-birth constructs of the VASA questionnaire, **multinomial logistic regression** was performed using “disease or condition directly leading to death” as dependent variables and elements of each section of the questionnaire as explanatory variables. Likelihood-Ratio (LR) test and Pseudo R-Square measures (Cox and Snell, Nagelkerke, and McFadden) were used as discriminant validity measures of the constructs.

Out of 100 cases, we had 33 stillbirths and 67 live birth cases. For the analysis, unequal prior probabilities of stillbirth and live birth were assumed and assigned prior probabilities as per the group size in the data. The deaths included in the study were both, i.e. ~~the home~~ home-based and institutional (formal and informal health care facilities). The Backward variable elimination method was used to compute the discriminant analysis model. It is vital to comment here at this point that the variables which are not included in the final model do not necessarily mean they are not important, but it means these variable(s) do not provide any additional statistical evidence ~~towards~~ for the classification of live and stillbirth.

The goodness of fit summary of discriminant analysis models of each of the pre-birth constructs of the VASA questionnaire is presented in Table 7~~6~~. The **eigenvalue (E.V.)** of canonical discriminant function is *the ratio of the between-groups sum of squares to within groups sum of squares*. A high value of eigenvalue indicates that the canonical function explains sufficiently higher variation in data. Similarly, the **canonical correlation (C.C.)** coefficient shows the

predictive ability of the canonical discriminant function. The value of C.C. ranges from 0 to 1, with a value closer to 1 showing the strong predictive ability of canonical discriminant function. The third goodness of fit statistics i.e., **Wilks’ Lambda** (ranging from 0 to 1), was calculated to be the proportion of the total variance in the discriminant scores not explained by differences among the groups. A small value of W.L. and statistically significant (p-value of <0.05) shows ~~the~~ a good fit of the discriminant model.

The goodness of fit summary of multinomial logistic regression models of each of during & post-birth constructs of the VASA questionnaire is presented in Table ~~6~~7. Statistically significant **Likelihood-Ratio (LR) test** result infers statistically significant improvement over the null model. **Pseudo R-Square measures (Cox and Snell, Nagelkerke, and McFadden)** ~~ranging~~ range from 0 to 1, with a value closer to “1” (one) ~~indicate~~ indicating a good model fit.

**Table 7~~6~~: Discriminant validation of CHERG’s VASA questionnaire**

| The goodness of fit summary of discriminant analysis models for ‘pre-birth constructs' | | | | | |
|---|---|---|---|---|---|
| VASA questionnaire constructs and sections | Eigenvalue | Canonical Correlation | Wilks' Lambda | | |
| Sections | | | Wilks' Lambda | Chi-square | p-value |
| Demographic and social constructs | 37.44 | 0.987 | 0.026 | 339.37 | <0.001 |
| Symptoms before pregnancy | 2.76 | 0.86 | 0.27 | 127.74 | <0.001 |
| Symptoms during initial two trimesters | 2.76 | 0.86 | 0.27 | 127.74 | <0.001 |
| Symptoms during last trimester | 91.13 | 0.871 | 0.241 | 422.91 | <0.001 |
| Care Seeking behavior for symptoms of last trimester | 1700.51 | 0.615 | 0.621 | 278.97 | <0.001 |
| Symptoms during labor and care seeking behavior | 120.88 | 0.92 | 0.154 | 446.68 | <0.001 |
| Treatment of labor symptoms and post delivery | 1042.16 | 0.993 | 0.013 | 646.35 | <0.001 |
| Knowledge Attitude and Behavior on maternal symptoms | 1475.99 | 0.952 | 0.093 | 682.34 | <0.001 |
| **The Goodness of fit summary of multinomial logistic regression models for ‘during and post-birth constructs'** | | | | | |
| **Sections** | **LR Tests (p-value)** | | **Cox and Snell** | **Nagelkerke** | **McFadden** |
| Birth of Child | <0.001 | | 0.968 | 0.996 | 0.963 |
| Physical Malformations of Child's Body | <0.001 | | 0.968 | 0.996 | 0.963 |
| Child Illness Details | <0.001 | | 0.968 | 0.996 | 0.963 |
| Health Seeking Behavior of Child Illness | <0.001 | | 0.968 | 0.996 | 0.963 |
| Health care professionals (HCP) involved during Child Illness | <0.001 | | 0.968 | 0.996 | 0.963 |
| Services Cost Burden and Delays | <0.001 | | 0.956 | 0.983 | 0.87 |
| Preventive Measures between childbirth and illness | <0.001 | | 0.968 | 0.996 | 0.963 |
| Community Efforts during child illness | <0.001 | | 0.956 | 0.983 | 0.87 |

| | | | | |
|---|---|---|---|---|
| Cause of death-Biological | <0.001 | 0.956 | 0.963 | 0.963 |
| Cause of death-Social | <0.001 | 0.964 | 0.985 | 0.89 |
| **C.V.**: Content validation; **D.V.**: Discriminant Validation; **R.A.:** Reliability Analysis; **I-CVI:** Item-content Validity Index; **E.V.:** Eigenvalue; **C.C.:** Canonical correlation; **W.L.:** Wilk's Lambda; **C.A.:** Cronbach's Alpha; | | | | |

A high eigenvalue of canonical discriminant function (>1), a high value of canonical correlation (>0.8), and a statistically significant Wilks' Lambda, with a p-value of <0.001, validate the good fit of discriminant models for pre-birth constructs of VASA questionnaire. A high value of Pseudo R-Square measures (>0.8) and a statistically significant LR test, with a p-value <0.001, validate the good fit of multinomial logistic regression models for during & post-birth constructs of the VASA questionnaire. Hence, underlying constructs of the VASA questionnaire, in all sections (Table 7~~6~~), were~~are~~ found to be distinctive and well-established to address the conceptual framework. More than 85.07% accuracy of the discriminant functions and the multinomial logistic regression model was observed in all sections of the VASA questionnaire.

## (d) Reliability assessment

**Formatted:** Font: Times New Roman

Reliability analysis measures the tool's capacity to provide a consistent result. Use of **Cronbach's Alpha** statistics was utilized to measure the reliability of constructs. Due to the dominantly dichotomous nature of data, the calculation of Cronbach's Alpha based on the Pearson correlations matrix would be incorrect or potentially very biased. Therefore, "hetcor" function of "polycor" package of R was used to compute the appropriate heterogeneous correlation matrix. The 'hetcor' function is capable of calculating Pearson correlations (for numeric data), polyserial correlations (for numeric and ordinal data), and polychoric correlations (for ordered or non-ordered factors). Reliability for the different sections of the VASA

questionnaire is presented in Table 7. The universal rule of thumb is that, if the value of **Cronbach's alpha** (C.A.) is 0.70 and more, the reliability is good, if the value of C.A. is between 0.80 and 0.90 the reliability is better, and if the value is 0.90 and beyond, the reliability is best. In our study, the Cronbach's Alpha values are above 0.90 across all the constructs of pre-birth, during & post-birth constructs of the VASA questionnaire (except “Demographic and social constructs” and “Care Seeking behaviour for symptoms of last trimester” which are 0.843 and 0.876 respectively)(Table 8~~7~~).

Classification of sensitivity, specificity, and accuracy of the models developed based on the elements of different sections of the VASA questionnaire are presented in Table 8~~7~~ below. 100% specificity, 100% sensitivity and 100% accuracy of the discriminant functions in ~~the~~ classifying births to live and stillbirths were observed in all sections, except, symptoms before pregnancy and symptoms during the initial ~~two~~ two-trimester sections of the VASA questionnaire. For which 100%, 72.73% and 91% of specificity, sensitivity, and accuracy were observed, and for these two sections lowest content validity was observed (see Table ~~7~~8). Cronbach's Alpha values we~~a~~re found higher than 0.80, indicating the reliability and internal consistency of the underlying constructs.

| **Table 8~~7~~: Reliability analysis of CHERG’s VASA questionnaire** | | | | |
|---|---|---|---|---|
| **VASA questionnaire constructs and section** | **Specificity (%)** | **Sensitivity (%)** | **Accuracy (%)** | **Cronbach's Alpha** |
| **Reliability accuracy of discriminant analysis models for pre-birth constructs** | | | | |
| Demographic and social constructs | 100.00~~%~~ | 100.00~~%~~ | 100.00~~%~~ | 0.843 |
| Symptoms before pregnancy | 100.00~~%~~ | 72.73~~%~~ | 91.00~~%~~ | 0.909 |
| Symptoms during initial two trimesters | 100.00~~%~~ | 72.73~~%~~ | 91.00~~%~~ | 0.944 |
| Symptoms during last trimester | 88.00~~%~~ | 100.00~~%~~ | 92.00~~%~~ | 0.894 |
| Care Seeking behaviour for symptoms of last trimester | 76.00~~%~~ | 100.00~~%~~ | 85.00~~%~~ | 0.876 |
| Symptoms during labor and care-seeking behaviour | 100.00~~%~~ | 100.00~~%~~ | 100.00~~%~~ | 0.954 |
| Treatment of labor symptoms and post delivery | 100.00~~%~~ | 100.00~~%~~ | 100.00~~%~~ | 0.933 |
| Knowledge Attitude and Behavior on maternal symptoms | 100.00~~%~~ | 100.00~~%~~ | 100.00~~%~~ | 0.940 |
| **Reliability and accuracy of multinomial logistic regression models for during and post-birth constructs** | | | | |
| Birth of Child | 100.00~~%~~ | 100.00~~%~~ | 100.00~~%~~ | 0.907 |

| | | | | |
|---|---|---|---|---|
| Physical Malformations of Child's Body | 100.00% | 24.20% | 75.00% | 0.947 |
| Child Illness Details | 100.00% | 100.00% | 100.00% | 0.948 |
| Health Seeking Behavior of Child Illness | 100.00% | 100.00% | 100.00% | 0.945 |
| Health care professionals (HCP) involved during Child Illness | 100.00% | 100.00% | 100.00% | 0.928 |
| Services Cost Burden and Delays | 100.00% | 100.00% | 100.00% | 0.95 |
| Preventive Measures between childbirth and illness | 100.00% | 100.00% | 100.00% | 0.973 |
| Community Efforts during child illness | 100.00% | 100.00% | 100.00% | 0.942 |
| Cause of death-Biological | 100.00% | 100.00% | 100.00% | 0.953 |
| Cause of death-Social | 100.00% | 100.00% | 75.00% | 0.973 |

# Discussion

The literature shows a lack of evidence on validation studies for integrated VASA tools for under-five years children. Globally, no research has been done on identifying the validity and reliability of CHERG's VASA questionnaire in diagnosing under-five children who died in a community-based household survey. Although the CHERG's integrated VASA tool was developed with a sound technical methodology and is based on the integration of several models and theories including the Three Delay model, and "The Pathway to Survival Conceptual Framework" (TPtoSCF)(Siddiqui et al., 2018),(CHERG, 2018a), still its validation for developing countries like Pakistan (where the huge estimates of under-five mortality lie) was technically required. After this current study, this validated tool will ensure the availability of a validated VASA tool that may give valid and reliable data pertaining to child mortality determinants operating in Pakistan. Such a tool was highly required to overcome the data shortage-related issues in the country, where the death registration is highly compromised(Abouzahr et al., 2014; Siddiqui et al., 2018; Siddiqui, Ng, & Low, 2016). When it comes to the reasons for having scanty data on death registration, one of the potential bottlenecks is the delayed and compromised notification of child deaths to the National Civil Registration

**Formatted:** Font: Times New Roman

and Vital Statistics Agency (NCRVS). Because the process of notification is time-consuming, there is no evidence of strict compliance among the general population in death notification(Abouzahr et al., 2014; Asad Mahmood Alvi, 1993). There are no financial benefits or assistance being provided to the community. The death registration process in the NCRVS is severely hampered, and this is true at all levels, from the local community to the national level. The existing data is also very limited and with questionable validity and accuracy(Abouzahr et al., 2014; Siddiqui et al., 2018; Siddiqui, Ng, & Low, 2016), this is particularly due to no implementation of standardized methods of data collection and reporting for death notification(Abouzahr et al., 2014; Zakar et al., 2018). Utilization and implementation of this validated tool in identifying mortality-related determinants and in assigning medical and social causes of child deaths, especially of those deaths which have been missed from the National Civil Registration and Vital Statistics will surely help us in overcoming data-related issues pertaining to child mortalities.

Validation studies usually encounter daunting challenges. Most of them include recall biases, information biases, small sample size estimation, etc. To overcome such challenges, the methodology involved in the validation studies should be very concrete and categoric, ensuring that the responses from the validated tool should be able to give information with acceptable accuracy~~ as per the reality of the situation~~. It is highly essential to undertake such relevant validation studies in developing countries so that it will make the validated version of the VASA tool available for conducting VASA investigations.

**Formatted:** Font: (Default) Times New Roman

**Formatted:** Font: (Default) Times New Roman

## ~~Validation of the VA tool~~

Formatted: Font: Times New Roman

Literature shows that there are several versions of VA tools developed by multiple agencies and research teams. Literature reflects several attempts ~~of~~ at validation studies of different VA tools that have utilized different methodologies to overcome such challenges and identified the precise CoD with much accuracy(Kahn et al., 1999; Mpimbaza et al., 2015; Murray et al., 2014; Rodriguez et al., 1998). The most commonly used VA tool which underwent validation is the World Health Organization's (WHO) VA tool(Aggarwal et al., 2013),(Nichols et al., 2018).

## ~~Sample size~~

Formatted: Font: Times New Roman

Having precisely calculated and appropriate sample size, the results of any validation study show the true validity and reliability of the tool. Compared to our VASA validation study, which included 100 deceased under-five cases, all the other VA validation studies undertook validation on a ~~a~~ comparatively larger sample size and implemented only the VA component(Aggarwal et al., 2013; Marsha et al., 2003; Nausheen et al., 2013; Soofi et al., 2015). We feel that having a smaller sample size is one of the limitations of our validation study. However, our sample size is purely based on general recommendations from Hertzog(Hertzog, 2008) (this was purely due to the reason that there are no specific guidelines for the sample size of respondents required for pre-survey validation of the VASA tool, hence this study followed the general guidelines from Hertzog guidelines), which suggests that the minimum sample size for any validation study should be 20% of the sample size calculated for the survey the tool will be ~~utilizing~~ utilized for. Aggarwal et al.(Aggarwal et al., 2013) included 313 neonatal deaths; Nausheen et al.(Nausheen et al., 2013) undertook validation on 204 neonatal deaths; the sample size in Soofi et al.(Soofi et

al., 2015) was 626 neonatal deaths, and Marsha et al.(Marsha et al., 2003) used 137 neonatal deaths.

Formatted: Font: (Default) Times New Roman

## ~~Recalling past events~~

Formatted: Font: Times New Roman

~~(a)~~ Conducting interviews after a long delay subsequent to the death event may affect the quality and reliability of data. This is most likely because the respondent needs to recall the events and happenings of different events that occurred before and around the death event. It is highly recommended that VASA interviews should be undertaken somewhere between 01 months till 06 months provided the family has come out of the mourning period(Serina et al., 2016). Conducting interviews within the mourning period may cause distress and influence the respondent's disposition and ability to engage in a VASA interview(Serina et al., 2016). P Serina et al.(Serina et al., 2016), suggest that a long recall period may limit the respondent's ability in recalling and recollecting pertinent facts. Events (or symptoms) with extraordinary severity (or implications) remain in the recall for a much more extended period compared to those with mild to moderate severity(Serina et al., 2016). Likewise all the other studies(Dikid et al., 2013; Marsha et al., 2003; Nausheen et al., 2013; Soofi et al., 2015), our VASA validation study undertook interviews within ~~the~~ six (06) weeks after the mortality event. In our sample size, none of the respondents had any difficulty ~~in~~ recalling the events.

Formatted: No bullets or numbering

## ~~Tool's accuracy~~

Formatted: Font: Times New Roman

The capacity of any questionnaire to correctly assign the proper CoD to any mortality with a degree of precision is called accuracy. When dealing with individual-level data, the diagnostic accuracy of the VASA tool is considered to be satisfactory when the specificity and sensitivity of

the questionnaire are a minimum of 90%(Soleman et al., 2006). However, at a much larger, i.e. national level, if the validation attempt of the VASA tool shows sensitivity and specificity of at least 50% and 90% respectively, the tool is usually considered to be having acceptable diagnostic accuracy(Soleman et al., 2006). ~~Our validation study defines specificity as the ability of the VA section of CHERG's VASA questionnaire to accurately and correctly assign the medical cause of death compared to the assigned CoD on the medical certificate as well as based on the signs and symptoms. The closeness of Medical CoD between the one assigned in the death certificate and the one assigned through VASA inquiry was used to measure the validity of the tool. Our~~This study found an accuracy of between 97% and 100% in assigning medical and social causes of child deaths respectively. In comparison, the accuracy of the WHO's VA tool validated in Aggarwals' study(Aggarwal et al., 2013) in identifying different neonatal causes of mortalities was found to be up to between 78% to 92% (except for birth asphyxia-16%); however, the kappa statistics was moderate from 0.46 to 0.55. Similarly, in Soofi et al. (Soofi et al., 2015) the WHO VA tool showed ~~the~~ a sensitivity of more than 83.5% for diagnosing different causes of neonatal mortality (except congenital malformation, which was 57%), while the specificity of all the major causes of neonatal deaths was found to be more than 90%. Marsha et al.(Marsha et al., 2003) found the sensitivity of the process of assigning the cause of death ranging from 39% (in diagnosing infection) to 90% (in diagnosing causes related to *too early/too small syndrome*) and specificity ranging from 67% (in diagnosing infection) and 99% (in diagnosing Neonatal Tetanus).

## Validation of VASA tools

The literature shows a lack of evidence on validation studies for integrated VASA tools for under-five years children. Globally, no research has been done on the identifying the validity and reliability of the CHERG's VASA questionnaire in diagnosing under-five children who died in a community-based household survey. Although the CHERG's integrated VASA tool was developed with a sound technical methodology and is based on the most holistic conceptual framework i.e., the "The Pathway to Survival Conceptual Framework" (TPtoSCF)(Siddiqui et al., 2018)(CHERG, 2018a), still its validation for developing countries like Pakistan (where the huge estimates of under-five mortality lie) is technically required. Such validated tool will ensure the availability of a validated VASA tool that may give valid and reliable data pertaining to child mortality determinants specifically operating specifically in Pakistan. Such a tool is highly required to overcome the data shortage related issues in the country, where the death registration are highly compromised(Abouzahr et al., 2014; Siddiqui et al., 2018; Siddiqui, Ng, & Low, 2016). When it comes to the reasons of having scanty data on death registration, one of the potential bottlenecks is the delayed and compromised notification of child deaths to the National Civil Registration and Vital Statistics Agency (NCRVS). Because the process of notification is time-consuming, there is no evidence of strict compliance among the general population in death notification(Abouzahr et al., 2014; Asad Mahmood Alvi, 1993). There are no financial benefits or assistance being provided to the community. The death registration process in the NCRVS is severely hampered, and this is true at all levels, from the local community to the national level. The existing data is also very limited and with questionable validity and accuracy(Abouzahr et al., 2014; Siddiqui et al., 2018; Siddiqui, Ng, & Low, 2016), this is particularly due to no implementation of standardized methods of data collection and reporting for death

Formatted: Font: Times New Roman

Formatted: Font: (Default) Times New Roman

~~notification(Abouzahr et al., 2014; Zakar et al., 2018). Utilization and implementation of this validated tool in identifying mortality related determinants and in assigning medical and social causes of child deaths, especially of those deaths which have been missed from the National Civil Registration and Vital Statistics will surely help us in overcoming data related issues pertaining to child mortalities.~~

**Formatted:** Font: (Default) Times New Roman

## Strengths of our validation study

**Formatted:** Font: Times New Roman

Our study is first in its kind as it validates the CHERG's integrated VASA tool, which is based on the most holistic conceptual framework, i.e. TPtoSCF (that addresses the barriers and limitations involved in accessing health care services), that have the capacity to record information required to develop policy in preventing under-five death incidents and improving the child survival estimates in developing countries (like Pakistan) where an unknown, but a large number of child mortality events have known to be missed and their medical causes have not been assigned.

Moreover, this study is ~~highly~~ important as it attempted a detailed validation of CHERG's integrated VASA tool by incorporating rigorous methodology and utilizing almost all the core methods suggested for identifying the validity and reliability evidence of any questionnaire(Taherdoost, 2018). The validation exercise yielded high I-CVI (>81.43%), with Cronbach's Alpha reliability statistics ~~is~~ being 0.843 for *"Demographic and social constructs"* and highest of 0.973 for *"Preventive Measures between childbirth and illness"*; the accuracy of between 81% (for *"Care seeking behaviour for symptoms of last trimester")* and 100% of the discriminants classifying births to live and stillbirths. The tool showed an accuracy of between 97% and 100% in assigning medical and social causes of child deaths respectively.

Although different VA questionnaires have been validated in Pakistan, however, some of them are specifically designed for the neonatal population(Soofi et al., 2015), while others are for stillbirths(Nausheen et al., 2013). The CHERG’s VASA integrated questionnaire encompasses three age groups and has not been validated for the Pakistani population.

An electronic CAPI (Computer Assisted Personal Interview) notebook format for data collection with skip patterns using CSPro software application has also been developed for this validated tool which was utilized for data collection during the Karachi’s VASA child mortality study. The primary purpose for transforming the lay paper-based form into a notebook application was to minimize the possibility of data collection and data entry errors. No data collection and data entry errors were encountered possibly due to extensive training of the field staff on the data collection and entry.

## Recommendations

**Formatted:** Font: Times New Roman

Future research should be geared to undertake the validation of different VA and SA tools (as separate validation exercises). This should be coupled with the efforts on enhanced research on the efficacy of the SA tool and to improve its capacity and accuracy for identifying social determinants pertaining to under-five mortality. Moreover, there is a strong need to have specific recommendations for the estimated sample size explicitly required for the validation process of the VASA tool. This would help future researchers to undertake analyses based on standardized recommendations. Additionally, it would be much better to conduct a quantitative and qualitative assessment on the respondents' point of view and their feedback towards the tool. Having

~~multiple versions of VA and SA tools from different agencies and research teams, there is a strong need to have a child centric VA, SA and VASA tools, which should be utilized globally after being validated.~~

Formatted: Font: (Default) Times New Roman

Formatted: Pattern: Clear (White)

# Conclusion

Based on our results, we confirm that the CHERG VASA integrated questionnaire is valid, reliable, and relevant to the conceptual frameworks for the Pakistani Population. The validated CHERG VASA tool can be utilized for establishing medical and social causes of Under-five child deaths in Karachi, Pakistan, and is one of the assets for Pakistan's child health policy with the potential to record relevant data and assign causes of child mortality cases occurring in Pakistan. The evidence extracted from the data and information obtained from the validated VASA tool will surely complement healthcare professionals and policymakers in improving the practice and modifying the policy for enhancing the survival of under-five children ~~of~~ in Karachi, Pakistan.

# Authors' contribution

Formatted: Font: Times New Roman

Conceptualization: M.B. Siddiqui

Methodology: M.B. Siddiqui

Implementation: M.B. Siddiqui

Software development: M.B. Siddiqui

Data collection: M.B. Siddiqui

Data entry: M.B. Siddiqui

Ensuring data completeness: M.B. Siddiqui

Analysis: M.B. Siddiqui

Writeup: M.B. Siddiqui

Writing– original draft: M.B. Siddiqui,

MBS, was involved in all aspects of this study, ranging from conceptualization, methodology, implementation, software development, data collection, data entry, ensuring data completeness, analysis, write up (original draft). MBS undertook complete biostatistical analyses, which was proofread by KA. CWN & WYL proofread the writeup. All authors have read and approved the final manuscript.


## Acknowledgement

**Formatted:** Font: Times New Roman

We would like to acknowledge ~~the health department, town health offices, and key personnel such as Dr. Sameera Ali Rizvi (SAR) and~~ the key personnel~~s~~ from the relevant communities for assisting the data collection process. SAR ~~also~~ participated in the proof~~-~~reading of some sections of this manuscript. We also would like to acknowledge the efforts of Dr Muhammad Akhter Hamid (MAH) who contributed ~~in~~ to PCVA. We would like to inform ~~you~~ that an unreviewed preprint of this manuscript is already available on Research Square at the link (https://www.researchsquare.com/article/rs-35489/v1) and Europe PMC (https://europepmc.org/article/ppr/ppr191893) with DOI (10.21203/rs.3.rs-35489/v1).


## List of abbreviations

**Formatted:** Font: Times New Roman

**C.A.:** Cronbach's Alpha

**C.C.:** Canonical correlation

**CHERG:** Child Health Epidemiology Reference Group

**CoD:** Cause of Death

**CRVS:** Civil Registration and Vital Statistics

**C.V.**: Content validation

**D.V.**: Discriminant Validation

**E.V.:** Eigenvalue

**HMIS:** Health Management Information System

**I-CVI:** Item-content Validity Index

**MCoD:** Medical Cause of Death

**PCVA:** Physicians certified VA technique

**R.A.:** Reliability Analysis

**SA:** Social Autopsy

**SCoD:** Medical Cause of Death

**SPSS:** Statistical Package for Social Sciences

**TPtoSCF:** The Pathway to Survival Conceptual Framework

**VA:** Verbal Autopsy **LR:** Likelihood-Ratio (LR) test

**VASA:** Verbal autopsy/Social Autopsy

**WHO:** World Health Organization

**W.L.:** Wilk’s Lambda~~;~~

# Declarations

**Formatted:** Font: Times New Roman

## Consent for publication

**Formatted:** Font: Times New Roman

Not applicable. (This research does not include any data from an individual person.)

# Availability of data and materials

Formatted: Font: Times New Roman

The datasets used and analyzed during the current study are available at https://figshare.com/s/883fe45ea7da71483acd.

Formatted: Font: (Default) Times New Roman, 12 pt

# Competing interests

Formatted: Font: Times New Roman

The author(s) declare(s) that they have no competing interests.

# Funding

Formatted: Font: Times New Roman

The current study has no funding attached.

# References

Formatted: Font: Times New Roman

Abouzahr, C., Azimi, S. Y., Bersales, L. G. S., Chandramouli, C., Hufana, L., Khan, K., Kulkayeva, G., Marskell, J., & Sauyekenova, L. (2014). Strengthening civil registration and vital statistics in the Asia-Pacific region: Learning from country experiences. *Asia-Pacific Population Journal*, *29*(1), 39–73.

Afzal, U., & Yusuf, A. (2013). The State of Health in Pakistan: An Overview. *The Lahore Journal of Economics*, *18*, 233–247. https://doi.org/http://www.lahoreschoolofeconomics.edu.pk/EconomicsJournal/LJEIntro.aspx

Aggarwal, A. K., Kumar, P., Pandit, S., & Kumar, R. (2013). Accuracy of WHO Verbal Autopsy Tool in Determining Major Causes of Neonatal Deaths in India. *PLoS ONE*, *8*(1). https://doi.org/10.1371/journal.pone.0054865

Anthoine, E., Moret, L., Regnault, A., Sbille, V., & Hardouin, J. B. (2014). Sample size used to validate a scale: A review of publications on newly-developed patient reported outcomes measures. *Health and Quality of Life Outcomes*, *12*(1), 2. https://doi.org/10.1186/s12955-014-0176-2

Asad Mahmood Alvi, B. (1993). *National Registration System in Pakistan Director General of Registration Government of Pakistan*. https://unstats.un.org/unsd/demographic/meetings/wshops/1993_China_CRVS/docs/1993_Doc.11_Pakistan.pdf

Bennet, A. E., & Ritchie, K. (1975). *Questionnaires in medicine A guide to their design and use*. http://trove.nla.gov.au/work/11045163?selectedversion=NBD793226
Bensaïd, K., Yaroh, A. G., Kalter, H. D., Koffi, A. K., Amouzou, A., Maina, A., & Kazmi, N. (2016). Verbal/Social Autopsy in Niger 2012-2013: A new tool for a better understanding of the neonatal and child mortality situation. *Journal of Global Health*, *6*(1), 10602. https://doi.org/10.7189/jogh.06.010602
CHERG. (2018a). *Projects: CHERG Verbal and Social Autopsy Survey Tools and Information*. http://cherg.org/projects/pathways.html#socialautopsy
CHERG. (2018b). *Projects: CHERG Verbal and Social Autopsy Survey Tools and Information*. CHERG. http://cherg.org/projects/pathways.html#socialautopsy
Dikid, T., Gupta, M., Kaur, M., Goel, S., Aggarwal, A. K., & Caravotta, J. (2013). Maternal and Perinatal Death Inquiry and Response project implementation review in India. *Journal of Obstetrics and Gynecology of India*, *63*(2), 101–107. https://doi.org/10.1007/s13224-012-0264-3
Gouda, H. N., Flaxman, A. D., Brolan, C. E., Joshi, R., Riley, I. D., AbouZahr, C., Firth, S., Rampatige, R., & Lopez, A. D. (2017). New challenges for verbal autopsy: Considering the ethical and social implications of verbal autopsy methods in routine health information systems. In *Social Science and Medicine*. https://doi.org/10.1016/j.socscimed.2017.05.002
Hertzog, M. A. (2008). Considerations in determining sample size for pilot studies. *Research in Nursing and Health*, *31*(2), 180–191. https://doi.org/10.1002/nur.20247
Hildenwall, H., Tomson, G., Kaija, J., Pariyo, G., & Peterson, S. (2008). "I never had the money for blood testing" - Caretakers' experiences of care-seeking for fatal childhood fevers in rural Uganda - A mixed methods study. *BMC International Health and Human Rights*, *8*. https://doi.org/10.1186/1472-698X-8-12
IGME, U. (2020). *Levels & Trend in Child Mortality: Estimates Developed by the Estimates developed by the UN Inter-agency Group for UN Inter-agency Group for Child Mortality Estimation Child Mortality Estimation*. UNICEF.
Kagabo, D. M., Kirk, C. M., Bakundukize, B., Hedt-Gauthier, B. L., Gupta, N., Hirschhorn, L. R., Ingabire, W. C., Rouleau, D., Nkikabahizi, F., Mugeni, C., Sayinzoga, F., & Amoroso, C. L. (2018). Care-seeking patterns among families that experienced under-five child mortality in rural Rwanda. *PLoS ONE*, *13*(1). https://doi.org/10.1371/journal.pone.0190739
Kahn, K., Tollman, S. M., Garenne, M., & Gear, J. S. S. (1999). Who dies from what? Determining cause of death in South Africa's rural north-east. *Tropical Medicine and International Health*, *4*(6), 433–441. https://doi.org/10.1046/j.1365-3156.1999.00415.x
Källander, K., Counihan, H., Cerveau, T., & Mbofana, F. (2019). Barriers on the pathway to survival for children dying from treatable illnesses in Inhambane province, Mozambique. *Journal of Global Health*, *9*(1), 1–14. https://doi.org/10.7189/JOGH.09.010809
Källander, K., Hildenwall, H., Waiswa, P., Galiwango, E., Peterson, S., & Pariyo, G. (2008). Delayed care seeking for fatal pneumonia in children aged under five years in Uganda: a case-series study .الرتجمة العربية لهذه الخالصة يف نهاية النص الكامل لهذه املقالة. *Bulletin of the World Health Organization*, *86*(5), 332–338. https://doi.org/10.2471/BLT.07.049353
Källander, K., Kadobera, D., Williams, T. N., Nielsen, R. T., Yevoo, L., Mutebi, A., Akpakli, J., Narh, C., Gyapong, M., Amu, A., & Waiswa, P. (2011). Social autopsy: INDEPTH Network experiences of utility, process, practices, and challenges in investigating causes and contributors to mortality. *Population Health Metrics*, *9*. https://doi.org/10.1186/1478-7954-9-44

Kalter, H. D., Roubanatou, A. M., Koffi, A., & Black, R. E. (2015). Direct estimates of national neonatal and child cause-specific mortality proportions in Niger by expert algorithm and physician-coded analysis of verbal autopsy interviews. *Journal of Global Health*, *5*(1), 10415. https://doi.org/10.7189/jogh.05.010415

Kalter, H. D., Salgado, R., Babille, M., Koffi, A. K., & Black, R. E. (2011). Social autopsy for maternal and child deaths: A comprehensive literature review to examine the concept and the development of the method. *Population Health Metrics*, *9*(45). https://doi.org/10.1186/1478-7954-9-45

Kalter, H., Yaroh, A. G., Maina, A., Koffi, A. K., Bensaïd, K., Amouzou, A., & Black, R. E. (2016). Verbal/social autopsy study helps explain the lack of decrease in neonatal mortality in Niger, 2007-2010. *Journal of Global Health*, *6*(1). https://doi.org/10.7189/jogh.06.010604

Koffi, A. K., Kalter, H. D., Loveth, E. N., Quinley, J., Monehin, J., & Black, R. E. (2017). Beyond causes of death: The social determinants of mortality among children aged 1-59 months in Nigeria from 2009 to 2013. *PLoS ONE*, *12*(5), e0177025. https://doi.org/10.1371/journal.pone.0177025

Koffi, A. K., Libite, P., Moluh, S., Wounang, R., & Kalter, H. D. (2015). Social autopsy study identifies determinants of neonatal mortality in Doume, Nguelemendouka and Abong–Mbang health districts, Eastern Region of Cameroon. *Journal of Global Health*, *5*(1). https://doi.org/10.7189/jogh.05.010413

Koffi, A. K., Maina, A., Yaroh, A. G., Habi, O., Bensaïd, & Kalter, H. D. (2016). Social determinants of child mortality in Niger: Results from the 2012 National Verbal and Social Autopsy Study. *Journal of Global Health*, *6*(1), 10603. https://doi.org/10.7189/jogh.06.010603

Koffi, A. K., Mleme, T., Nsona, H., Banda, B., Amouzou, A., & Kalter, H. D. (2015). Social autopsy of neonatal mortality suggests needed improvements in maternal and neonatal interventions in Balaka and Salima districts of Malawi. *Journal of Global Health*, *5*(1), 010416. https://doi.org/10.7189/jogh.05.010416

Koffi, A. K., Perin, J., Kalter, H. D., Monehin, J., Adewemimo, A., & Black, R. E. (2019). How fast did newborns die in Nigeria from 2009-2013: a time-to-death analysis using Verbal /Social Autopsy data. *Journal of Global Health*, *9*(2), 020501. https://doi.org/10.7189/jogh.09.020501

Lisa-Marie Thomas, Lucia D'Ambruoso, D. B. (2018). Verbal autopsy in health policy and systems: a literature review. *BMJ Global Health*, *3*(2). https://doi.org/10.1136/ bmjgh-2017-000639

Marsha, D. R., Sadruddin, S., Fikree, F. F., Krishnan, C., & Darmstadt, G. L. (2003). Validation of verbal autopsy to determine the cause of 137 neonatal deaths in Karachi, Pakistan. *Paediatric and Perinatal Epidemiology*, *17*(2), 132–142. https://doi.org/10.1046/j.1365-3016.2003.00475.x

Midhet, F. (2008). Validating the verbal autopsy questionnaire for maternal mortality in pakistan. *International Journal of Health Sciences*, *2*(1), 91–96. http://www.pubmedcentral.nih.gov/articlerender.fcgi?artid=3068724&tool=pmcentrez&rendertype=abstract

Moyer, C. A., Johnson, C., Kaselitz, E., & Aborigo, R. (2017). Using social autopsy to understand maternal, newborn, and child mortality in low-resource settings: a systematic review of the literature. *Global Health Action*, *10*(1), 1413917.

https://doi.org/10.1080/16549716.2017.1413917

Mpimbaza, A., Filler, S., Katureebe, A., Quick, L., Chandramohan, D., & Staedke, S. G. (2015). Verbal autopsy: Evaluation of methods to certify causes of death in Uganda. *PLoS ONE*, *10*(6), e0128801. https://doi.org/10.1371/journal.pone.0128801

Murray, C. J. L., Lozano, R., Flaxman, A. D., Serina, P., Phillips, D., Stewart, A., James, S. L., Vahdatpour, A., Atkinson, C., Freeman, M. K., Ohno, S. L., Black, R., Ali, S. M., Baqui, A. H., Dandona, L., Dantzer, E., Darmstadt, G. L., Das, V., Dhingra, U., … Lopez, A. D. (2014). Using verbal autopsy to measure causes of death: The comparative performance of existing methods. *BMC Medicine*, *12*(1), 5. https://doi.org/10.1186/1741-7015-12-5

Nausheen, S., Soofi, S. B., Sadiq, K., Habib, A., Turab, A., Memon, Z., Khan, M. I., Suhag, Z., Bhatti, Z., Ahmed, I., Bahl, R., Bhutta, S., & Bhutta, Z. A. (2013). Validation of Verbal Autopsy Tool for Ascertaining the Causes of Stillbirth. *PLoS ONE*, *8*(10), e76933. https://doi.org/10.1371/journal.pone.0076933

Navale, S., Habumugisha, L., Amoroso, C., Sayinzoga, F., Gupta, N., & Hirschhorn, L. R. (2017). Exploring Drivers of Infant Deaths in Rural Rwanda Through Verbal Social Autopsy. *Annals of Global Health*, *83*(5–6), 756–766. https://doi.org/10.1016/j.aogh.2017.10.029

Nichols, E. K., Byass, P., Chandramohan, D., Clark, S. J., Flaxman, A. D., Jakob, R., Leitao, J., Maire, N., Rao, C., Riley, I., & Setel, P. W. (2018). The WHO 2016 verbal autopsy instrument: An international standard suitable for automated analysis by InterVA, InSilicoVA, and Tariff 2.0. *PLoS Medicine*, *15*(1), e1002486. https://doi.org/10.1371/journal.pmed.1002486

Nisar, Y. Bin, & Dibley, M. J. (2014). Determinants of neonatal mortality in Pakistan: Secondary analysis of Pakistan Demographic and Health Survey 2006-07. *BMC Public Health*, *14*(1). https://doi.org/10.1186/1471-2458-14-663

Nonyane, B. A. S., Kazmi, N., Koffi, A. K., Begum, N., Ahmed, S., Baqui, A. H., & Kalter, H. D. (2016). Factors associated with delay in care-seeking for fatal neonatal illness in the Sylhet district of Bangladesh: Results from a verbal and social autopsy study. *Journal of Global Health*, *6*(1). https://doi.org/10.7189/jogh.06.010605

Rodriguez, L., Reyes, H., Tome, P., Ridaura, C., Flores, S., & Guiscafre, H. (1998). Validation of the Verbal Autopsy Method to Ascertain Acute Respiratory Infection as Cause of Death. *Indian Journal of Pediatrics*, *65*(4), 579–584. https://doi.org/10.1007/BF02730899

Schumacher R  Diallo MO, Keita DR, Kalter HD, Pasha O:, S. E. (2002). *Mortality study in Guinae: Investigating the causes of death for children under 5 The Basic Support for Institutionalizing Child Survival Project (BASICS II). Arlington, VA; 2002*. http://www.basics.org/documents/pdf/guinea_mort.pdf

Serina, P., Riley, I., Hernandez, B., Flaxman, A. D., Praveen, D., Tallo, V., Joshi, R., Sanvictores, D., Stewart, A., Mooney, M. D., Murray, C. J. L., & Lopez, A. D. (2016). What is the optimal recall period for verbal autopsies? Validation study based on repeat interviews in three populations. *Population Health Metrics*, *14*(1). https://doi.org/10.1186/s12963-016-0105-1

Siddiqui, M. B., Ng, C. W., & Low, W. Y. (2016). Social Autopsy is a Dire Need for Investigating Child Mortality in Pakistan. *International Journal of Endorsing Health Science Research (Ijehsr)*, *4*(2), 01. https://doi.org/10.29052/ijehsr.v4.i2.2016.01-04

Siddiqui, M. B., Ng, C. W., & Low, W. Y. (2017). To Identify the Non-biological Causes of Child Mortalities in Developing Countries, Social Autopsy Tools should be Based on "The

Pathway to Survival Conceptual Framework." *International Journal of Endorsing Health Science Research (Ijehsr)*, *5*(1), 01. https://doi.org/10.29052/ijehsr.v5.i1.2017.01-04

Siddiqui, M. B., Ng, C. W., Low, W. Y., Noushad, S., Ahmed, S., & Syed, S. (2014). Challenges and acceptance of the use of computer-assisted personal interviewtechnology for verbal autopsy/social autopsy child mortality survey in urban slums of Karachi, Pakistan. *International Journal of Endorsing Health Science Research (Ijehsr)*, *6*(2), 30. https://doi.org/10.29052/ijehsr.v6.i2.2018.30-42

Siddiqui, M. B., Ng, C. W., Low, W. Y., Syed, S., Ahmed, S., Noushad, S., Ali, A., Fatima, K., & Mirza, M. (2016). A Verbal/Social Autopsy (VASA) Child Mortality Inquiry to Investigate Under-Five Mortality Determinants in Slums of Karachi, Pakistan:A Mix Methods Interventional Study. *International Journal of Endorsing Health Science Research (Ijehsr)*, *4*(4), 01. https://doi.org/10.29052/ijehsr.v4.i4.2016.01-11

Siddiqui, M. B., Ng, C. W., & Yun Low, W. (2018). Methodologies of Verbal autopsy and Social autopsy tools, adopted by VASA studies in exploring under-five mortality determinants. *International Journal of Endorsing Health Science Research (Ijehsr)*, *6*(3), 43. https://doi.org/10.29052/ijehsr.v6.i3.2018.43-55

Soleman, N., Chandramohan, D., & Shibuya, K. (2006). Verbal autopsy: current practices and challenges. *Bull.World Health Organ*, *84*(0042-9686 (Print)), 239–245. http://www.scopus.com/inward/record.url?eid=2-s2.0-33644795635&partnerID=40&md5=09ffcd11e7668e66416b1919445bb9d2

Soofi, S. B., Ariff, S., Khan, U., Turab, A., Khan, G. N., Habib, A., Sadiq, K., Suhag, Z., Bhatti, Z., Ahmed, I., Bhal, R., & Bhutta, Z. A. (2015). Diagnostic accuracy of WHO verbal autopsy tool for ascertaining causes of neonatal deaths in the urban setting of Pakistan: A hospital-based prospective study. *BMC Pediatrics*, *15*(1), 1–9. https://doi.org/10.1186/s12887-015-0450-4

Taherdoost, H. (2018). Validity and Reliability of the Research Instrument; How to Test the Validation of a Questionnaire/Survey in a Research. *SSRN Electronic Journal*, *5*(3), 28–36. https://doi.org/10.2139/ssrn.3205040

UN-Habitat. (2003). Slums of the World: the face of urban poverty in the new millennium. In *UN-Habitat, Nairobi*.

Waiswa, P. K., Kallander, S., Peterson, G., Tomson, G., & Pariyo. (2010). Using the three delays model to understand why newbornbabies die in eastern Uganda. *Tropical Medicine & International Health*, *8*(8), 964–972. https://doi.org/doi:10.1111/j.1365-3156.2010.02557.x

WHO/UNICEF. (2015). *WHO/UNICEF Joint Monitoring Programme for water supply and sanitation: Sanitation tables*. Who/Unicef. http://www.wssinfo.org/data-estimates/tables/

WHO, & Department of Measurement and Health Information Systems evidence and information for policy. (2004). *WHO Technical Consultation on Verbal Autopsy Tools: Review of the literature and currently-used verbal autopsy tools*. 1–45. http://www.who.int/healthinfo/statistics/mort_verbalautopsy.pdf

Zakar, M. Z., Zakar, R., Mustafa, M., Jalil, A., & Fischer, F. (2018). Underreporting of stillbirths in Pakistan: Perspectives of the parents, community and healthcare providers. *BMC Pregnancy and Childbirth*, *18*(1). https://doi.org/10.1186/s12884-018-1924-9

# Supporting information

**S1 Fig. Flow chart of the validation process**

**S2 VASA Questionnaire**

**Formatted:** Font: Times New Roman

## AUTHOR'S COMMENTS/COMPLIANCE ON REVIEWERS' COMMENTS

**Validation of CHERG'S Verbal Autopsy-Social Autopsy (VASA) tool for ascertaining determinants and causes of under-five child mortality in Pakistan.**

**Muhammad Bilal Siddiqui[1,3*], Chiu Wan Ng[1], Wah Yun Low[2], Khadijah Abid[3]**

[1]Department of Social and Preventive Medicine, Faculty of Medicine, University of Malaya, Kuala Lumpur, Malaysia.
[2]Dean's Office, Faculty of Medicine, University of Malaya, Kuala Lumpur, Malaysia.
[3]Department of Public Health, Faculty of Life Sciences. Research Villa, Shaheed Zulfiqar Ali Bhutto Institute of Science and Technology (SZABIST), Clifton, Karachi, Pakistan.

***Corresponding Author:**
**Muhammad Bilal Siddiqui**
Email: muhammad.bilal@szabist.edu.pk (MBS)
drbilals@siswa.um.edu.my

| AUTHORS COMMENTS | AUTHOR'S COMPLIANCE/COMMENTS |
|---|---|
| Reviewer #4: Line28,66,68: the term mortalities is not appropriate – needs to be corrected as mortality – including the title | (Done, see title, please see whole document. I have amended your suggestion throughout the document) |
| Line 210: what was the educational background of the data collectors | (Done. please see line 406-407) |
| Table1: the column for frequency can only depict n. denominator of 100 from each cell needs to be removed. The percent column can mention (%) in column heading & the % symbol from each cell can be removed. | (Done, please see all tables) |
| All Tables: The percent column can mention (%) in column heading & the % symbol from each cell can be removed. | (Done, please see all tables) |
| Table 1:please provide details about unimproved & improved facility mentioned under access to toilets | (described in the table 1 at the bottom of the respective table. Also referenced). |
| Table 7: cause of death biological – sensitivity % is missing | (Done, please see table no-08) |
| Methods section could include details about the characteristics of the deceased children. Age & gender, cause of death, hospitalization, institutional delivery, antenatal history, hospital records if available etc | (Done in table 5) |
| Line 443:avoid duplication of word | (Done) |

| | |
|---|---|
| specifically | |
| Line 467: it is preferable to avoid superlatives like highly important | (Done) |
| Line 484-485: the term error is missing from the statement | (Done, please see Line no 1351) |
| Line 490-492: the recommendation is not well suited, considering the present study objectives & hence needs to be removed. | (Deleted) |
| Reviewer #5: Sample Size:<br>What was the data source for identification of mothers who had lost their Under five children at each of the 12 sites listed? Authors suggest that death registration is far from complete in Karachi city (deleted from the manuscript as the statement was confusing) yet they claim mothers were randomly included based on Proportional uota sampling. How was this done? Please clarify? (Done. clarified between lines 225-257) | (deleted from the manuscript as the statement was confusing)<br><br>(Done. clarified between lines 225-257) |
| Line 131 135 : "These 12 locations throughout the city of Karachi were chosen based on criteria to identify geographical points throughout the entire city where residents belonging to the most common and frequently found ethnic backgrounds (Punjabi, Sindhi, Balochi, Siraiki, Pathan, Urdu speaking, Kashmiri, Hindko,and Brahvi) live within at least 2 kilometres of residential locations. This criterion, ensured that the sample is representative of the general population." Unclear? What do the authors mean by living within 2 kms of residential locations? Please rephrase. | (typo error. Rewritten. Done, please see Line 225-252) |
| Line 170: Authors describe CHERG VASA tool as a uantitative tool. This is not so. It includes open ended text fields as well. | (Done, please see Line 355-357) |
| Line 216-218: Authors have not elaborated details of PCVA process. Did a single physician review a particular VA and assign most probable CoD? If so it introduces a subjective bias? Did more than one physician independently review each VA form? If so what was the agreement between physicians and how were disagreements adjudicated? How were the responses made available to the physicians? It is well known that in PCVA | (explained between lines 412-419). |

| | |
|---|---|
| CoD is largely assigned based on the text narrative rather than the close ended responses. What measures were taken to minimize this phenomenon? | |
| In the PAPI what was the completion rate for the entire questionnaire after accounting for skip patterns? | (Done, please see Line no 401-403) |
| Section V5.12 - V5.16 of the CHERG VASA questionnaire record CoD listed on the death certificate. Was this information masked from the Physicians? If not it calls into question the very high values of 97% accuracy for CoD assigned based on VA and may invalidate the findings of the entire study. Please give details | (Done, Line no: 410-431) |

Data Review URL

Click here to download Data Review URL
http://figshare.com/s/883fe45ea7da71483acd

Click here to access/download
**Preprint PDF**
Researchsquare preprint validation paper vasa.pdf
