# Supplementary material for: Validation of CHERG’S Verbal Autopsy-Social Autopsy (VASA) tool for ascertaining determinants and causes of under-five child mortality in Pakistan
Source: PLoS One. 2023 Dec 18;18(12):e0278149. doi: 10.1371/journal.pone.0278149 (PMC10727362; doi:10.1371/journal.pone.0278149)
Supplement: Attachment — Submitted filename: Response to Reviewers.doc [file pone.0278149.s003.doc]

**RESPONSE TO REVIEWERS**

Note: The line numbers mentioned in this document corresponds with the line numbers in the **“Manuscript”** and not from the **“Revised Manuscript with Track Changes”**. The res of some more changes are highlighted in Yellow which were additional changes identified and modified after the further review.

| **S. No.** | **Comments** | **COMPLIANCE RESPONSE** |
| --- | --- | --- |
| **REVIEWERS-1 COMMENTS** | | |
|  | Line 40 (abstract): Criteria for classifying countries as high priority needs to be discussed. | Deleted and modified as per the suggestion. The introduction section of abstract has been modified as per the suggestion. |
|  | Line 41 (abstract): The proportion in terms of actual numbers/percentage of under-five mortalities in the countries mentioned can be included here | Deleted and modified as per the suggestion. The introduction section of abstract has been modified as per the suggestion. |
|  | Lines 42-43 (abstract): Basis for stating that “an unknown but major proportion of such mortalities…” needs to be mentioned. | Explained. The introduction section of abstract has been modified as per the suggestion. |
|  | Line 97 : Proper definition and differentiation between verbal and social autopsy needs to be highlighted. In addition, the impression given by the sentences is that they are useful primarily in low resource settings whereas the utility of verbal and social autopsies is multi-contextual. | Mentioned. Lines no 55-63. |
|  | Line 125 : Seems like a redundant statement which does not add anything important to the paper. | Deleted. Line 94. |
|  | Lines 131-132 : The authors need to explain the difference between the CHERG’s SA tool and CHERG’s integrated VASA tool. Or at least state the basis for the difference. | Deleted the mention of CHERG’s SA tool, as it caused a confusion in understanding. Lines 99-100. |
|  | Lines 138-140 : Kindly reframe the sentence as it is difficult to understand why this VASA integrated tool study is one of a kind. | Reframed. Lines 105-107. |
|  | Lines 147-149 : It is not clear whether Karachi’s VASA child mortality study and Karachi’s VASA study (VASA survey) are the same or different entities. | Reframed. Lines 147-150. Also wherever, such discrepancy of mentioning existed, was corrected as per the suggestion. |
|  | Lines 152-155 : It would be helpful if the authors explained why they went in for general guidelines rather than VASA specific guidelines while determining the sample size for this validation study. | Explained. Lines 120-127. |
|  | Line 171 : The location of the Advance Educational Institute and Research Centre needs to be clarified. Is it based in Pakistan? Or is it a Malaysian/Canadian based centre. The authors also need to explain why local ethical clearance was not needed/not sought for a study involving Pakistani citizens carried out in Pakistan. | Explained. This paper is in continuation of a Phd study conducted by the corresponding author (M. Bilal Siddiqui) who is a student in University of Malaya, hence the university’s ethical clearance was sought. Moreover, the study was conducted in Karachi, Pakistan, the local (Karachi, Pakistan’s) ethical clearance was also sought by AEIRC. Lines 153-157. |
|  | Line 176 : It would give the study better credence if all the “many more” subject specialists were mentioned fully. | Removed the phrase ‘many more’ lines 211-212, as none of the specialists other than the mentioned ones were involved. It was a typing and writing error. |
|  | Lines 179-179 : the methodology of Physician certified verbal autopsy CoD and Algorithm Certified Verbal Autopsy CoD should be expanded on. | It is again a writing error. The Algorithm Certified Verbal Autopsy CoD was not utilized. Only, the Physician certified verbal autopsy CoD was performed. Corrected and explained the Physician certified verbal autopsy CoD. Lines 212-217. |
|  | Lines 365-366 : The statements pertain only to the WHO VA tool without a comparison with the CHERG VA tool. It needs to be specified if these are separate tools. Though it has been mentioned that the CHERG tools are the result of collaboration between the WHO and UNICEF, the reasons for development of a child centric VA tool needs to be brought in. | This is a good idea for having a child centric VA tool, rather than different versions of VA tools. In the manuscript, a statement has been added that the WHO VA tool is different from the CHERG VA tool. Lines 374-378. |
|  | Lines 370-373 : As mentioned earlier, it would be helpful if the authors explained why they went in for Hertzog guidelines rather than VASA specific guidelines while determining the sample size for this validation study. | In the manuscript it was initially mentioned that there are succinct specific guidelines on sample size of respondents required for pre-survey validation exercise of VASA tool, however, it was a mistakenly written statement, and actually there are no specific guidelines for it, hence because of this the authors have followed the general guidelines from Hertzog guidelines in the manuscript. The statement has been updated now. Lines 120-123, 381-392. |
|  | Lines 415-418 : The sentence is difficult to comprehend. Kindly look into simplifying the sentence/breaking it up so that the reader can better understand what the authors are trying to convey. | Corrected. Lines 433-435. |
|  | Lines 424-426 : Some of the reasons for the compromised birth and death registration can be mentioned as well. Doing so will further stress on the need for using VASA tools. | Added. Lines 441-449 |
|  | Lines 426-427: As above, the reasons for the questionable accuracy and validity of the data can be added here. | Modified. Lines 449-452. |
|  | Lines 454-456: The authors have mentioned that a CAPI notebook format has been developed to minimize data entry errors. It is suggested that the authors elaborate on the frequency of such errors using the paper-based form and whether there was a possibility of their study also having such data entry errors. | This validation study did not encounter any errors between the CAPI and PAPI platforms, however, no inconsistencies were encountered between the PAPI and CAPI platforms. The only difference between the two platforms was ease of data collection and less time utilized for data collection by the data collectors. Lines 480-484. |
|  | Lines 461-464 : The authors mention a need to have “specific recommendations for the estimated sample size explicitly…..”. However, earlier in the article they have stated that “literature shows very succinct specific guidelines on the sample size OF RESPONDENTS required for pre-survey validation exercise of VASA tool” (lines 152-153). This is a contradiction. | The manuscript still recommends that there is a need to have “specific recommendations for the estimated sample size explicitly required for the validation process of the VASA tool. however, earlier in the manuscript, it was a mistakenly written statement that “literature shows very succinct specific guidelines on the sample size OF RESPONDENTS required for pre-survey validation exercise of VASA tool” (lines 120-123, 381-392.). Actually, there are no specific guidelines for it, hence because of this the authors have followed the general guidelines from Hertzog guidelines in the manuscript. These statements has been updated now where ever it was mentioned such in the manuscript. |
| **REVIEWERS-2 COMMENTS** | | |
|  | Line 290: The authors needs to clarify, whether the still birth / live birth mentioned in the study, where due to Institutional delivery with proper health care assistance or home delivery by dhais | Added. Lines 301-302. |
|  |  |  |
| **REVIEWERS-2 COMMENTS** | | |
|  | **Introduction**  1) It would be helpful if authors provide some statistics pertaining the number of under 5-mortality rates in few lower middle-income countries, particularly Pakistan. This is important, as it stresses and contextualizes statistically the extremely high number of these premature deaths, which builds a stronger case for the use of these tools. | The overall text in the introduction has been modified. Lines 21-23. |
|  | **Methods**  2) Which slums from Karachi were selected? Important mention the names for complete transparency as this links directly to their socio-economic class. | Mentioned as suggested. Lines 128-136. |
|  | **Methods**  3) Table 1: in VA-6, any examples of the open-ended questions which were asked? | Mentioned in the table itself. Table-2, Line 184. |
|  | **Results**  4) Results should provide more background details. How many women were approached who refused to answer the questionnaire? Reasons for refusal? (important to state this was not because they found the questionnaire tough to interpret) | Mentioned in the section of ‘***Discriminant validity (construct validity) assessment.*** Lines 268-335. |
|  | **Results**  5) Some more details regarding face validation should be added. Of the 100 respondents, did all of them understand every question and option clearly? If not, adding a table might make this more clear | the face validation was undertaken by the panel of experts and not by the respondents from the deceased family. Lines 236-243. |
|  | **Results**  6) A good chunk of results includes details regarding how content validation/discrimination etc was done and the details regarding statistical analysis. Would suggest authors to have a separate subsection of statistical methods at the end of Methods where they can describe all the statistical models used. In results, the main focus should be the outcome of these analyses which are mentioned very briefly right now. I would suggest authors to also mention in text the key points from tables, especially for Table 4. | the method adopted for calculating the I CVI index through content validation and discriminant validation is explicitly mentioned in the section of content validation. Both of these validation processes included statistical analysis using statistical analysis software. Lines (247-265)(271-322). The explanation of the analysis is already mentioned in details in separate sections of content (247-265) and discriminant (268-335) validity. As per your suggestion, if the explanation of analysis is given as a separate section after the methods section, I feel that the reader might get confused with presentation of explanation. However, if you strongly feel that it should be done, then kindly re-suggest in your review comments, so that I work on it. |
|  | **Discussion**  7) Line 426-427: Authors should comment and compare the results of VASA validity observed in their analysis with that existing in literature. | There is no prior study on the validation of VASA tool, hence the discussion did not include the comparison, however, the VA and SA results were compared with the most relevant studies. Lines 432-433. |
|  | **Discussion**  8) This study has many limitations which must be acknowledged in a separate paragraph. First, data was collected from one city only in a limited number of people. Second, did all the respondents belong to low socio-economic class? Third, since all interviews were conducted, interview bias can play a pivotal role. | The issue of generalizability was already addressed while planning the sampling methodology of this validation study. The sampling methodology was not mentioned in the manuscript, now it has been explained in detail. Lines 119-144.  There was a mixture of respondents from all the wealth quintiles. Please see Table-4. Line 233.  All the interviews were conducted from a random sample. Line 32 and line 128. |
|  | **Minor**  Line 54: “one hundred randomly selected mothers of deceased , with a recent child death event” would suggest authors to revise this line to amend the grammatical error | Corrected. Lines 32-33. |
|  | **Minor**  Line 152: Would request the authors to review why ‘OF RESPONDENTS’ is capitalized? | Typographical error. It has been corrected now. Line 120. |
