# Supplementary material for: Validation of CHERG’S Verbal Autopsy-Social Autopsy (VASA) tool for ascertaining determinants and causes of under-five child mortality in Pakistan
Source: PLoS One. 2023 Dec 18;18(12):e0278149. doi: 10.1371/journal.pone.0278149 (PMC10727362; doi:10.1371/journal.pone.0278149)
Supplement: Attachment — Submitted filename: Response to Reviewer 7 sept 22.doc [file pone.0278149.s004.doc]

**AUTHOR’S COMMENTS/COMPLIANCE ON REVIEWERS’ COMMENTS**

**Validation of CHERG’S Verbal Autopsy-Social Autopsy (VASA) tool for ascertaining determinants and causes of under-five child mortality in Pakistan.**

**Muhammad Bilal Siddiqui^1,3*^, Chiu Wan Ng^1^, Wah Yun Low^2^, Khadijah Abid^3^**

**^1^**Department of Social and Preventive Medicine, Faculty of Medicine, University of Malaya, Kuala Lumpur, Malaysia.

**^2^**Dean’s Office, Faculty of Medicine, University of Malaya, Kuala Lumpur, Malaysia.

**^3^**Department of Public Health, Faculty of Life Sciences. Research Villa, Shaheed Zulfiqar Ali Bhutto Institute of Science and Technology (SZABIST), Clifton, Karachi, Pakistan.

***Corresponding Author:**

**Muhammad Bilal Siddiqui**

Email: muhammad.bilal@szabist.edu.pk (MBS)

drbilals@siswa.um.edu.my

| **AUTHORS COMMENTS** | **AUTHOR’S COMPLIANCE/COMMENTS** |
| --- | --- |
| Reviewer #4: Line28,66,68: the term mortalities is not appropriate – needs to be corrected as mortality – including the title | (Done, see title, please see whole document. I have amended your suggestion throughout the document) |
| Line 210: what was the educational background of the data collectors | (Done. please see line 406-407) |
| Table1: the column for frequency can only depict n. denominator of 100 from each cell needs to be removed. The percent column can mention (%) in column heading & the % symbol from each cell can be removed. | (Done, please see all tables) |
| All Tables: The percent column can mention (%) in column heading & the % symbol from each cell can be removed. | (Done, please see all tables) |
| Table 1:please provide details about unimproved & improved facility mentioned under access to toilets | (described in the table 1 at the bottom of the respective table. Also referenced). |
| Table 7: cause of death biological – sensitivity % is missing | (Done, please see table no-08) |
| Methods section could include details about the characteristics of the deceased children. Age & gender, cause of death, hospitalization, institutional delivery, antenatal history, hospital records if available etc | (Done in table 5) |
| Line 443:avoid duplication of word specifically | (Done) |
| Line 467: it is preferable to avoid superlatives like highly important | (Done) |
| Line 484-485: the term error is missing from the statement | (Done, please see Line no 1351) |
| Line 490-492: the recommendation is not well suited, considering the present study objectives & hence needs to be removed. | (Deleted) |
| Reviewer #5: Sample Size: What was the data source for identification of mothers who had lost their Under five children at each of the 12 sites listed? Authors suggest that death registration is far from complete in Karachi city (deleted from the manuscript as the statement was confusing) yet they claim mothers were randomly included based on Proportional uota sampling. How was this done? Please clarify? (Done. clarified between lines 225-257) | (deleted from the manuscript as the statement was confusing)  (Done. clarified between lines 225-257) |
| Line 131 135 : "These 12 locations throughout the city of Karachi were chosen based on criteria to identify geographical points throughout the entire city where residents belonging to the most common and frequently found ethnic backgrounds (Punjabi, Sindhi, Balochi, Siraiki, Pathan, Urdu speaking, Kashmiri, Hindko,and Brahvi) live within at least 2 kilometres of residential locations. This criterion, ensured that the sample is representative of the general population." Unclear? What do the authors mean by living within 2 kms of residential locations? Please rephrase. | (typo error. Rewritten. Done, please see Line 225-252) |
| Line 170: Authors describe CHERG VASA tool as a uantitative tool. This is not so. It includes open ended text fields as well. | (Done, please see Line 355-357) |
| Line 216-218: Authors have not elaborated details of PCVA process. Did a single physician review a particular VA and assign most probable CoD? If so it introduces a subjective bias? Did more than one physician independently review each VA form? If so what was the agreement between physicians and how were disagreements adjudicated? How were the responses made available to the physicians? It is well known that in PCVA CoD is largely assigned based on the text narrative rather than the close ended responses. What measures were taken to minimize this phenomenon? | (explained between lines 412-419). |
| In the PAPI what was the completion rate for the entire questionnaire after accounting for skip patterns? | (Done, please see Line no 401-403) |
| Section V5.12 - V5.16 of the CHERG VASA questionnaire record CoD listed on the death certificate. Was this information masked from the Physicians? If not it calls into question the very high values of 97% accuracy for CoD assigned based on VA and may invalidate the findings of the entire study. Please give details | (Done, Line no: 410-431) |
