# Supplementary material for: Validation of CHERG’S Verbal Autopsy-Social Autopsy (VASA) tool for ascertaining determinants and causes of under-five child mortality in Pakistan
Source: PLoS One. 2023 Dec 18;18(12):e0278149. doi: 10.1371/journal.pone.0278149 (PMC10727362; doi:10.1371/journal.pone.0278149)
Supplement: Attachment — Submitted filename: Response to Reviewer 16 October 2022.doc [file pone.0278149.s006.doc]

**AUTHOR’S COMMENTS/COMPLIANCE ON REVIEWERS’ COMMENTS**

**Validation of CHERG’S Verbal Autopsy-Social Autopsy (VASA) tool for ascertaining determinants and causes of under-five child mortality in Pakistan.**

**Muhammad Bilal Siddiqui^1,3*^, Chiu Wan Ng^1^, Wah Yun Low^2^, Khadijah Abid^3^**

**^1^**Department of Social and Preventive Medicine, Faculty of Medicine, University of Malaya, Kuala Lumpur, Malaysia.

**^2^**Dean’s Office, Faculty of Medicine, University of Malaya, Kuala Lumpur, Malaysia.

**^3^**Department of Public Health, Faculty of Life Sciences. Research Villa, Shaheed Zulfiqar Ali Bhutto Institute of Science and Technology (SZABIST), Clifton, Karachi, Pakistan.

***Corresponding Author:**

**Muhammad Bilal Siddiqui**

Email: muhammad.bilal@szabist.edu.pk (MBS)

[drbilals@siswa.um.edu.my](mailto:drbilals@siswa.um.edu.my), [drbilals@gmail.com](mailto:drbilals@gmail.com)

| **COMMENTS** | **AUTHOR’S COMPLIANCE/COMMENTS** |
| --- | --- |
| The editor commented on the references and updating the references.  **Comment**: “Please review your reference list to ensure that it is complete and correct. If you have cited papers that have been retracted, please include the rationale for doing so in the manuscript text or remove these references and replace them with relevant current references. Any changes to the reference list should be mentioned in the rebuttal letter that accompanies your revised manuscript. If you need to cite a retracted article, indicate the article’s retracted status in the References list and also include a citation and full reference for the retraction notice.” | The reference list was updated. On line number 74 in the manuscript prior to the current revision and line no 108 in the manuscript submitted after the current R2 review comments, there were some references ^4,6–9^ was corrected as these were typing error. There errors have been deleted.  Moreover, between lines 71 and 82 in the manuscript prior to the current revision, there were some references which were mistakenly repeated. Hence these doubled references were removed, which can be verified between the line no 100-109 in the manuscript submitted after the current R2 review comments. |
| **Additional Editor Comments:** On deleting the name of one author, Mr Hassan Ahmed from the manuscript. | (An email has been sent to Editor Plos One for explaining the rationale of removing Mr Hassan Ahmed from the authors list). The editor has replied back to me on 13th October 2021 saying that the editor has contacted the author for clarification, and I may contact the editor back if I did not hear from editor in the next one week. |
| **Reviewer #5:** Authors have largely addressed issues raised by this reviewer in the earlier review. However certain suggestions have been made. Please see reviewer comments attached as notes in the uploaded reviewed manuscript. Request address the same. | 1. Table-1: Check table heading and column content: I have deleted “X (SAMPLE SIZE)” 2. Line 169 in the manuscript prior to the current R2 revision: I have replaced “counted” to “included”. See line no 339 in the manuscript after the current R2 review comments. 3. Line 186 in the manuscript prior to the current R2 revision: I have replaced “quantitative” to “semi-quantitative”. See line no 356 in the manuscript submitted after the current R2 review comments. 4. Line 203 in the manuscript prior to the current R2 revision: check grammar. The grammar of the sentence starting from Line no 201-203 (in the manuscript submitted after the current R2 review comments) has been improved. |
| **Reviewer #6:** I have few comments that can be incorporated into the paper:  1. Full form of I-CVI should be mentioned in abstract  2. Line 405-413: The author can change this part of the manuscript and add the points to introduction and should begin their study by the summary of their results.  3. Line 413-415: A brief explanation of how their study ensures availability of the tool, can be incorporated in the manuscript. | 1. Done in the abstract. See line no 54 in the manuscript submitted after the current R2 review comments. 2. Line 405-413 The contents were deleted from discussion. Such details were already available in Introduction, so to avoid duplicacy, the deleted points were not added in Introduction. The discussion was initiated with the summary of the results of this validation study. 3. Line 413-415: The line was rephrased into much better context and avoided the concept of “ensuring the availability of the tool”. |
|  | I have added my personal email address (drbilals@gmail.com), which in case if I am not accessible on the initial two email addresses, can be use for contacting me. If it is not acceptable by the journal, you have complete rights to not to include in the manuscript. |
|  | In the abstract line no 30-31 in the manuscript submitted after the current R2 review comments, the term CHERG was explained with a full form as “Child Health Epidemiology Reference Group”. I felt that it might have been missed from reviewers, hence I corrected it from my end. If it is not acceptable by the journal, you have complete rights to not to include in the manuscript. |
